# Superiority of the Triple-Acting 5-HT_6_R/5-HT_3_R Antagonist and MAO-B Reversible Inhibitor **PZ-1922** over 5-HT_6_R Antagonist Intepirdine in Alleviation of Cognitive Deficits in Rats

**DOI:** 10.1021/acs.jmedchem.3c01482

**Published:** 2023-10-05

**Authors:** Katarzyna Grychowska, Uriel López-Sánchez, Mathieu Vitalis, Geoffrey Canet, Grzegorz Satała, Agnieszka Olejarz-Maciej, Joanna Gołębiowska, Rafał Kurczab, Wojciech Pietruś, Monika Kubacka, Christophe Moreau, Maria Walczak, Klaudia Blicharz-Futera, Ophélie Bento, Xavier Bantreil, Gilles Subra, Andrzej J. Bojarski, Frédéric Lamaty, Carine Becamel, Charleine Zussy, Séverine Chaumont-Dubel, Piotr Popik, Hugues Nury, Philippe Marin, Laurent Givalois, Paweł Zajdel

**Affiliations:** † Faculty of Pharmacy, Jagiellonian University Medical College, 9 Medyczna Str., 30-688 Kraków, Poland; ‡ Univ. Grenoble Alpes, CNRS, CEA, IBS, F-38000 Grenoble, France; § Molecular Mechanisms in Neurodegenerative Dementia (MMDN) Laboratory, University of Montpellier, EPHE-PSL, INSERM U1198, 34-095 Montpellier, France; ∥ Faculty of Medicine, Laval University, CR-CHUQ, G1 V 4G2 Québec City (QC), Canada; ⊥ Maj Institute of Pharmacology, Polish Academy of Sciences, 12 Smętna Str., 31-324 Kraków, Poland; # IBMM, Université de Montpellier, CNRS, ENSCM, 34-293 Montpellier, France; ¶ Institut de Génomique Fonctionnelle, Université de Montpellier, CNRS, INSERM, 34-094 Montpellier, France; ∇ CNRS, 75-016 Paris, France

## Abstract

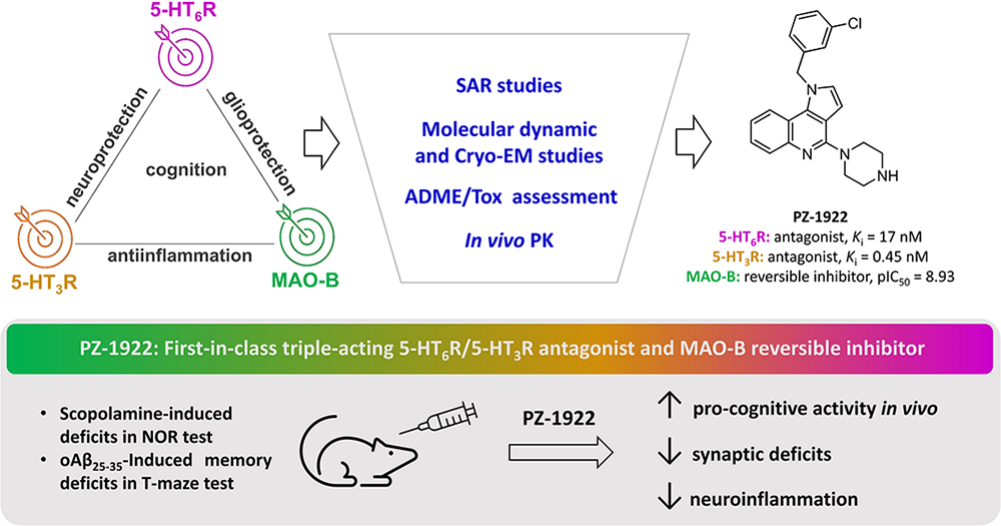

The multifactorial origin and neurochemistry of Alzheimer’s disease (AD) call for the development of multitarget treatment strategies. We report a first-in-class triple acting compound that targets serotonin type 6 and 3 receptors (5-HT-Rs) and monoamine oxidase type B (MAO-B) as an approach for treating AD. The key structural features required for MAO-B inhibition and 5-HT_6_R antagonism and interaction with 5-HT_3_R were determined using molecular dynamic simulations and cryo-electron microscopy, respectively. Bioavailable **PZ-1922** reversed scopolamine-induced cognitive deficits in the novel object recognition test. Furthermore, it displayed superior pro-cognitive properties compared to intepirdine (a 5-HT_6_R antagonist) in the AD model, which involved intracerebroventricular injection of an oligomeric solution of amyloid-β peptide (oAβ) in the T-maze test in rats. **PZ-1922**, but not intepirdine, restored levels of biomarkers characteristic of the debilitating effects of oAβ. These data support the potential of a multitarget approach involving the joint modulation of 5-HT_6_R/5-HT_3_R/MAO-B in AD.

## Introduction

Alzheimer’s disease (AD) patients suffer from progressive impairment of cognitive functions and emotional instability. At the molecular level, the development of AD is associated with the formation of amyloid-β (Aβ) plaques and *tau* protein tangles, followed by a neuroinflammation process consecutive to the activation of astrocytes and microglia. These processes are accompanied by biological changes, including synaptic loss, neuronal death, and disturbances in neurotransmission.^1^

The recent fiasco of biological therapy in the treatment of AD, together with the multifactorial origin of neurodegenerative diseases such as AD, has led to the reevaluation of the strategies for small molecules research, from the conventional single-target concept to the development of pleiotropic agents. These compounds are designed to target two or more seemingly unrelated proteins, triggering intricate neurochemical changes that aim to overcome the therapeutic burdens of available drugs. Promising examples of such multitarget drugs include ladostigil,^2^ which acts as a dual-acting inhibitor of acetylcholinesterase (AChE) and monoamine oxidase type B (MAO-B), as well as donecopride,^3^ a serotonin type 4 receptor agonist (5-HT_4_R) and AChE inhibitor. These compounds are currently undergoing preclinical or clinical evaluation as potential treatments for AD.

In this study, we conceptualized compounds capable of antagonizing 5-HT_6_R and 5-HT_3_R and inhibiting the activity of MAO-B as a novel strategy holding promise for treating AD. This is because 5-HT_6_R has a unique distribution in brain regions that are responsible for cognitive functions. In addition, preclinical data have demonstrated cognition-enhancing properties of 5-HT_6_R antagonists in rodent models of AD,^4,5^ schizophrenia,^6^ and neuropathic pain.^7^ Additionally, a proof-of-concept clinical trial has confirmed the efficacy of intepirdine (a 5-HT_6_R antagonist) in alleviating cognitive deficits in AD. Based on these findings, we considered the blockade of 5-HT_6_R to be a relevant mechanism for the development of multitarget drugs in the context of AD.^8−10^

In AD patients, there is a notable loss of cholinergic neurons and a deficiency in acetylcholine in specific brain regions. Modulating 5-HT_3_R may help mitigate these issues. By blocking presynaptic 5-HT_3_Rs, the hyperactivity of mesolimbic dopamine and excessive release of γ-aminobutyric acid (GABA) can be inhibited, thus increasing cholinergic neurotransmission in the hippocampus and cortex. Additionally, the blockade of postsynaptic 5-HT_3_R located on GABAergic interneurons can enhance glutamatergic transmission,^11−13^ thereby promoting cognitive functions. The blockade of 5-HT_3_Rs also induces anti-inflammatory and neuroprotective effects against Aβ-induced neurotoxicity.^14^

Reversible inhibition of MAO-B, a flavin adenine dinucleotide (FAD)-dependent enzyme, located in glial cells and mainly expressed in the cortical and hippocampal regions of the brain, has recently been considered a valuable therapeutic approach for AD.^15^ Preclinical studies demonstrated that reversible MAO-B inhibitors, as opposed to irreversible ones, can ameliorate cognitive deficits in transgenic mice with APP/PS1 mutations following a 4 week treatment.^16^ This positive effect could be explained by the contribution of MAO-B to GABA-ergic transmission. Irreversible MAO-B inhibitors activate diamine oxidase (DAO) to compensate for the loss of MAO-B activity. However, this mechanism can lead to GABA-mediated astrogliosis. In contrast, reversible inhibitors do not induce DAO-dependent compensatory mechanisms, thus avoiding the shortcomings associated with irreversible inhibitors.^16^

Through structure–activity studies within a group of 1*H*-pyrrolo[3,2-*c*]quinoline compounds, we identified compound **PZ-1922**, which exhibited antagonistic properties at both receptors, along with potent inhibitory activity at MAO-B. To understand the key structural motifs required for 5-HT_6_R antagonist properties and MAO-B inhibition,^17^ we conducted *in silico* analysis. Additionally, the fragments required for the interaction with 5-HT_3_R were revealed by using cryo-electron microscopy (cryo-EM) imaging. **PZ-1922** displayed favorable oral absorption and penetration into the central nervous system (CNS). *In vivo*, it effectively reversed memory deficits induced by scopolamine (SCOP) in the novel object recognition (NOR) test in rats and demonstrated the ability to prevent Aβ-induced memory decline in the T-maze test in both curative and preventive approaches. The compound also affected various biochemical parameters associated with Aβ-induced neuroinflammation, synaptic deficits, and apoptosis. These data demonstrate the potential of a multitarget approach that involves the simultaneous targeting of 5-HT_6_R, 5-HT_3_R, and MAO-B in the development of new anti-AD agents.

## Results

### Strategy Design and Summary of the Structure–Activity Relationship Studies

**Scheme 1:**
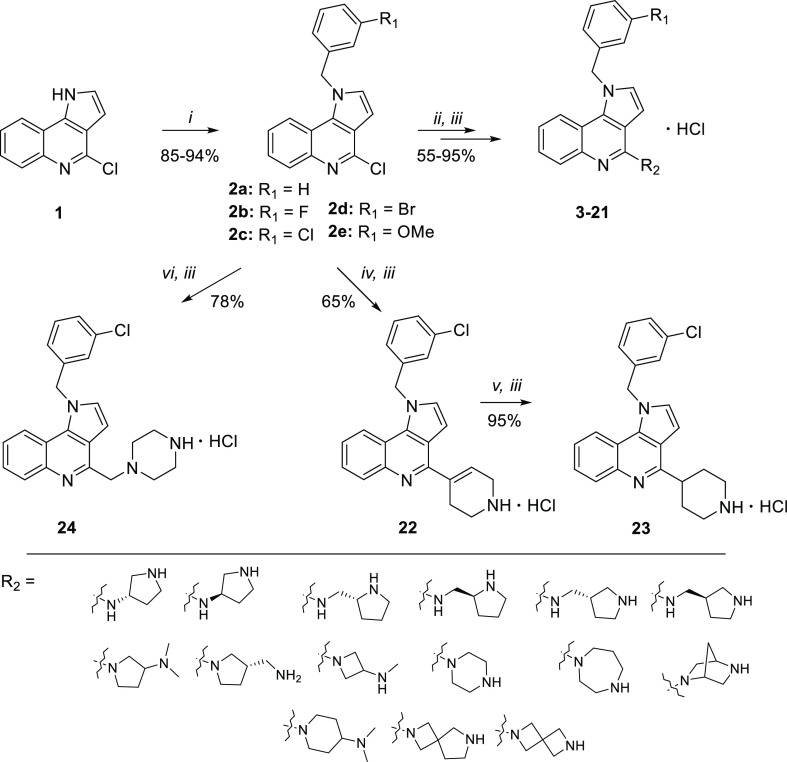
Synthetic Pathway Leading to 1*H*-Pyrrolo[3,2-*c*]quinoline Derivatives **3**–**24** (i) BTPP (Phosphazene Base P1-*t*-Bu-tris(tetramethylene), Respective Benzyl Bromide, CH_2_Cl_2_, 36 °C, 12 h, Yield 85–94% (ii) Primary Amine, *t*-BuONa, BINAP (2,2′-Bis(diphenylphosphino)-1,1′-binaphthyl), Pd_2_(dba)_3_ (Tris(dibenzylideneacetone)dipalladium(0)), Dioxane/*t*-BuOH 3/1, 90 °C, 1 h, MW or Secondary Amine, Acetonitrile, TEA, 140 °C, 7 h, MW, Yield 55–95% (iii) 1 M HCl/MeOH, rt, 12 h, Yield 95–99% (iv) 3,6-Dihydro-2*H*-pyridine-1-*N*-Boc-4-boronic Acid Pinacol Ester, Pd(dppf)Cl_2_ ([1,1′-Bis(diphenylphosphino)ferrocene]dichloropalladium(II)), K_2_CO_3_, Water, 80 °C, 6 h, MW, Yield 65% (v) 10% Pd/C, Ethanol, rt, 2 h, Yield 95% (vi) Potassium ((4-(*tert*-Butoxycarbonyl)piperazin-1-yl)methyl)trifluoroborate, Pd[(C_6_H_5_)_3_P]_4_ (Tetrakis(triphenylphosphine)palladium(0)), K_2_CO_3_, Dioxane/Water 90 °C, 5 h, Yield 78%.

Our design strategy relied on identifying the specific structural features required for 5-HT_6_R/5-HT_3_R antagonists^10^ and selective reversible MAO-B inhibitors^17^ within the 1*H*-pyrrolo[3,2-*c*]quinoline framework. To achieve this, we systematically diversified substituted benzyl fragments at the *N*^1^ position and diverse alicyclic amine moieties at position *C*^4^ of the central core. To synthesize the compounds, we adapted a function-oriented synthesis method previously developed for 4-chloro-1*H*-pyrrolo[3,2-*c*]quinoline (**1**) (Scheme 1).^17^

**Figure 1:**
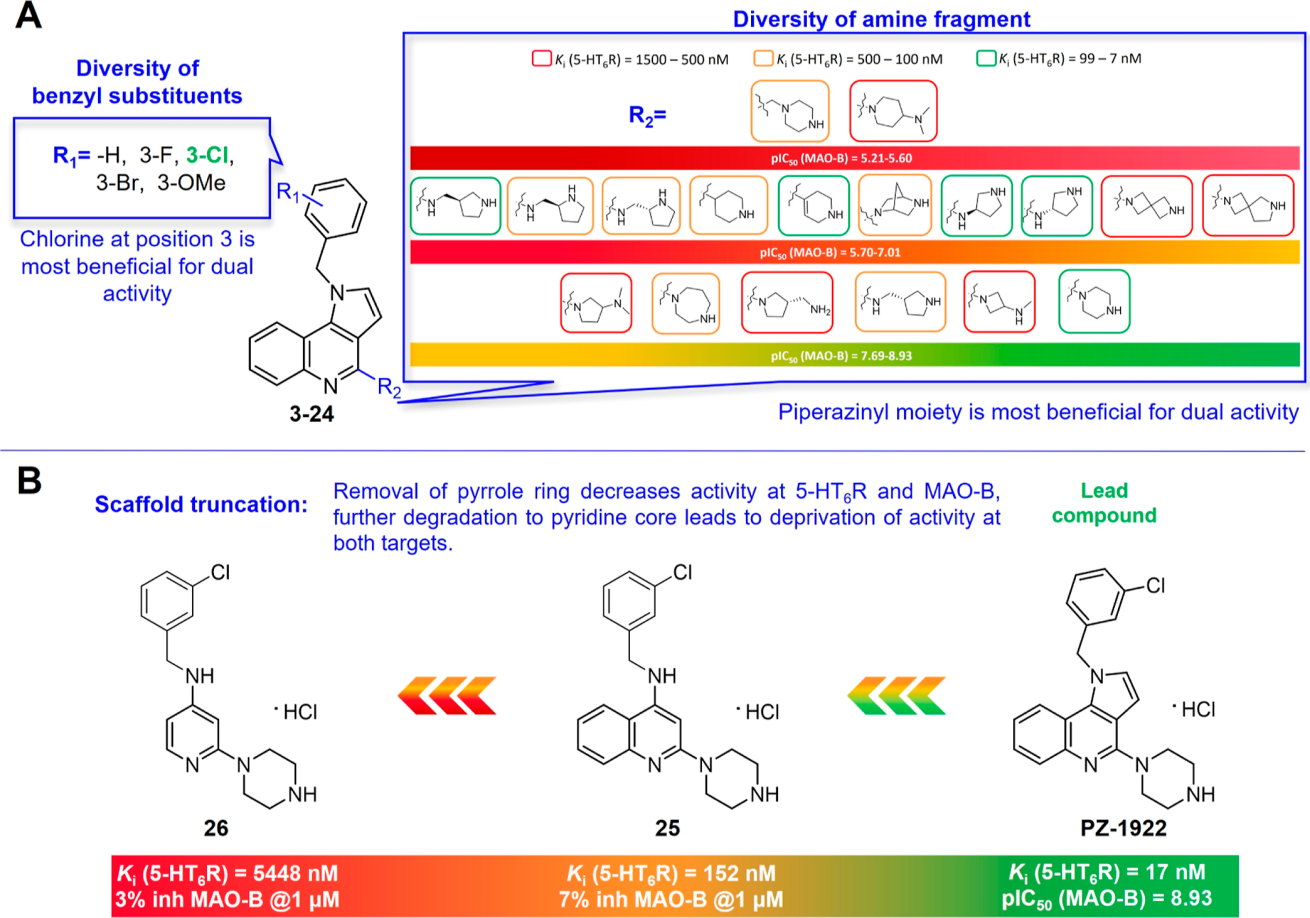
Summary of the SAR explored around 1*H*-pyrrolo[3,2-*c*]quinolines (3–24) and their molecular simplification analogues (25, 26) with a focus on inhibition of MAO-B and antagonism at 5-HT_6_R. (A) Structural diversity of benzyl substituents in the *N*^1^ position and alicyclic amine fragments in the *C*^4^ position. (B) 1*H*-Pyrrolo[3,2-*c*]quinoline as a privileged core for dual MAO-B inhibitory and 5-HT_6_R antagonistic properties.

Briefly, alkylation of compound **1** with the respective benzyl bromides in the presence of BTPP yielded benzyl derivatives **2a–2e**. Subsequent coupling with primary amines under Buchwald–Hartwig *N*-arylation conditions provided compounds **3**–**12** (Table 1), while reactions with secondary amines, carried out under prolonged microwave heating in acetonitrile in the presence of triethylamine (TEA), yielded compounds **13**–**21**.^17^ The 1,2,3,6-tetrahydropyridin-4-yl derivative **22** was synthesized by Suzuki coupling of 4-chloro-1-(3-chlorobenzyl)-1*H*-pyrrolo[3,2-*c*]quinoline **2c** with *N*-Boc-1,2,5,6-tetrahydropyridine-4-boronic acid pinacol ester, whereas its saturated congener **23** was obtained from **22** by reduction of the double bond using palladium on activated charcoal under a hydrogen atmosphere. Compound **24** was synthesized by coupling of **2c** with potassium salt of Boc-protected (piperazin-1-yl)methyltrifluoroborate in the presence of tetrakis(triphenylphosphine)-palladium and K_2_CO_3_. This allowed us to access a series of 21 novel benzyl derivatives of 1*H*-pyrrolo[3,2-*c*]quinoline (**3**–**24**) that were modified with different alicyclic amines (Table 1 and Figure 1). Treatment of the obtained compounds with a 1 M HCl solution in methanol (MeOH) removed the Boc-protecting group and converted amine derivatives into their HCl salts (**3**–**24**). Additionally, two compounds (**25**, **26**) containing structurally simplified quinoline and pyridine cores (Figure 1) were synthesized (for detailed description, see Supporting Information, Scheme S1).

**Table 1:**
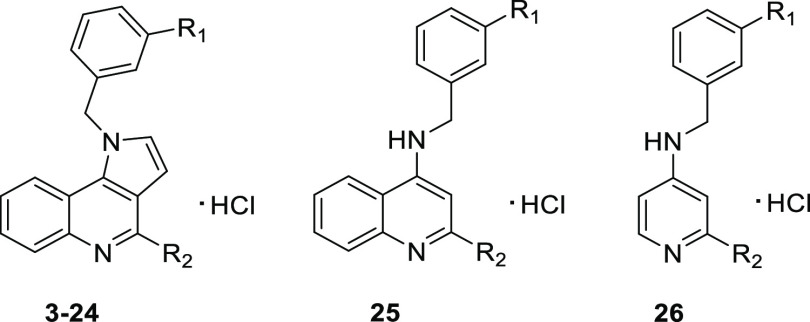

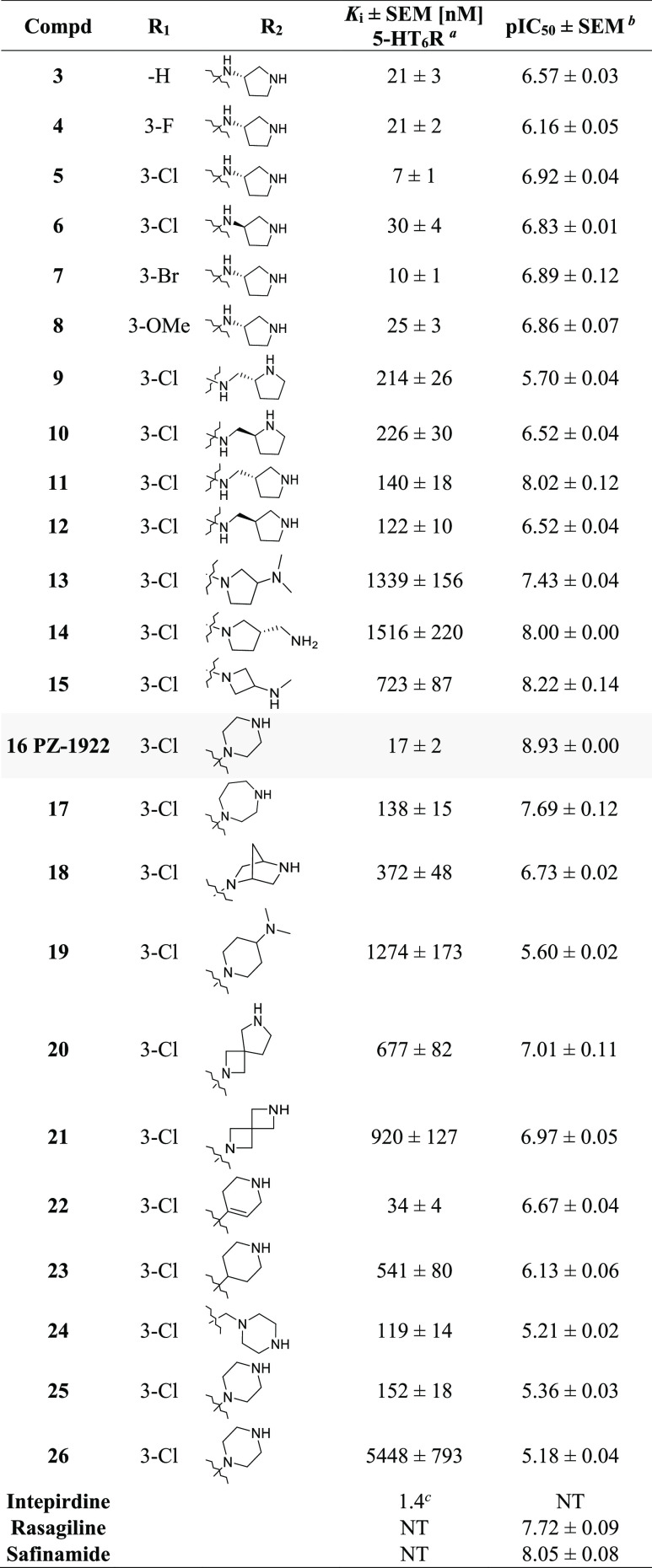
Affinity for 5-HT_6_R and Potency for MAO-B Inhibition of Synthesized Compounds **3–26** and Reference 5-HT_6_R Ligands and MAO-B Inhibitors

a Mean *K*_i_ values ± SEMs (standard error of the mean) based on three independent binding experiments in HEK293 cells stably expressing *h*5-HT_6_R.

b pIC_50_ values ± SEMs based on two experiments run in duplicate, determined by fluorometric method using human recombinant MAO-B and rasagiline [1 μM] as a positive control.

c Data taken from ref (18).

Our first goal was to verify the impact of the structural diversification on the affinity for 5-HT_6_R and the inhibitory activity at MAO-B. We aimed to identify compounds that exhibited dual activity within the nanomolar range, which would then be further evaluated for their activity at 5-HT_3_R. Among the various substituents at position 3 of the benzyl fragment (compounds **3**–**8**), the introduction of a chlorine atom proved to be the most favorable modification resulting in increased affinity for 5-HT_6_R and providing the most potent inhibitory activity at MAO-B.

The inclusion of different alicyclic amine fragments at position 4 of the 1*H*-pyrrolo[3,2-*c*]quinoline core, which are critical for affinity toward class A G-protein-coupled receptors (GPCRs), did not hamper the inhibitory activity at the MAO-B enzyme. The size and geometry of the alicyclic ring had an impact on the formation of a salt bridge between the basic center and the respective amino acid residue in the binding site of the biological targets. This, in turn, determined the activity and selectivity of the compounds (Figure 1 and Table 1). It is worth noting that alicyclic amines with exocyclic amino groups (*N*,*N*-dimethylpyrrolidin-3-amine, (*S*)-pyrrolidin-3-ylmethanamine, *N*-methylazetidin-3-amine, and *N*,*N*-dimethylpiperidin-4-amine) were detrimental for binding to 5-HT_6_R (compounds **13**, **14**, **15**, **19**). However, alicyclic amines containing pyrrolidine and azetidine moieties (*N*,*N*-dimethylpyrrolidin-3-amine, (*S*)-pyrrolidin-3-ylmethanamine, *N*-methylazetidin-3-amine) showed potent inhibitory activity at MAO-B (compounds **13**–**15**). On the other hand, spirocyclic amines did not exhibit favorable binding to either 5-HT_6_R or inhibition of MAO-B (compounds **20**, **21**).

Structure–activity relationship (SAR) evaluation revealed that the piperazine ring at the *C*^4^ position of the 1*H*-pyrrolo[3,2-*c*]quinoline core was the only fragment that ensured the desired pharmacological profile at both 5-HT_6_R and MAO-B and (*K*_i_ (5-HT_6_R) = 17 nM, pIC_50_ (MAO-B) = 8.93).

To further demonstrate the role of the planar tricyclic scaffold in the interaction with both targets, we conducted experiments in which the fused pyrrole ring was removed from the pyrroloquinoline core (Table 1, Scheme S1). The resulting quinoline congener (**25**) exhibited a significant drop in affinity for 5-HT_6_R and reduced inhibitory activity at MAO-B. Furthermore, we performed additional structural simplification to obtain a compound (**26**) with a pyridine core. This compound was devoid of activity at both targets, indicating that the presence of the 1*H*-pyrrolo[3,2-*c*]quinoline core is required for dual action on both 5-HT_6_R and MAO-B (Figure 1).

### **PZ-1922** Behaves as an Antagonist at 5-HT_6_R/5-HT_3_R and Reversibly Inhibits MAO-B

**Figure 2:**
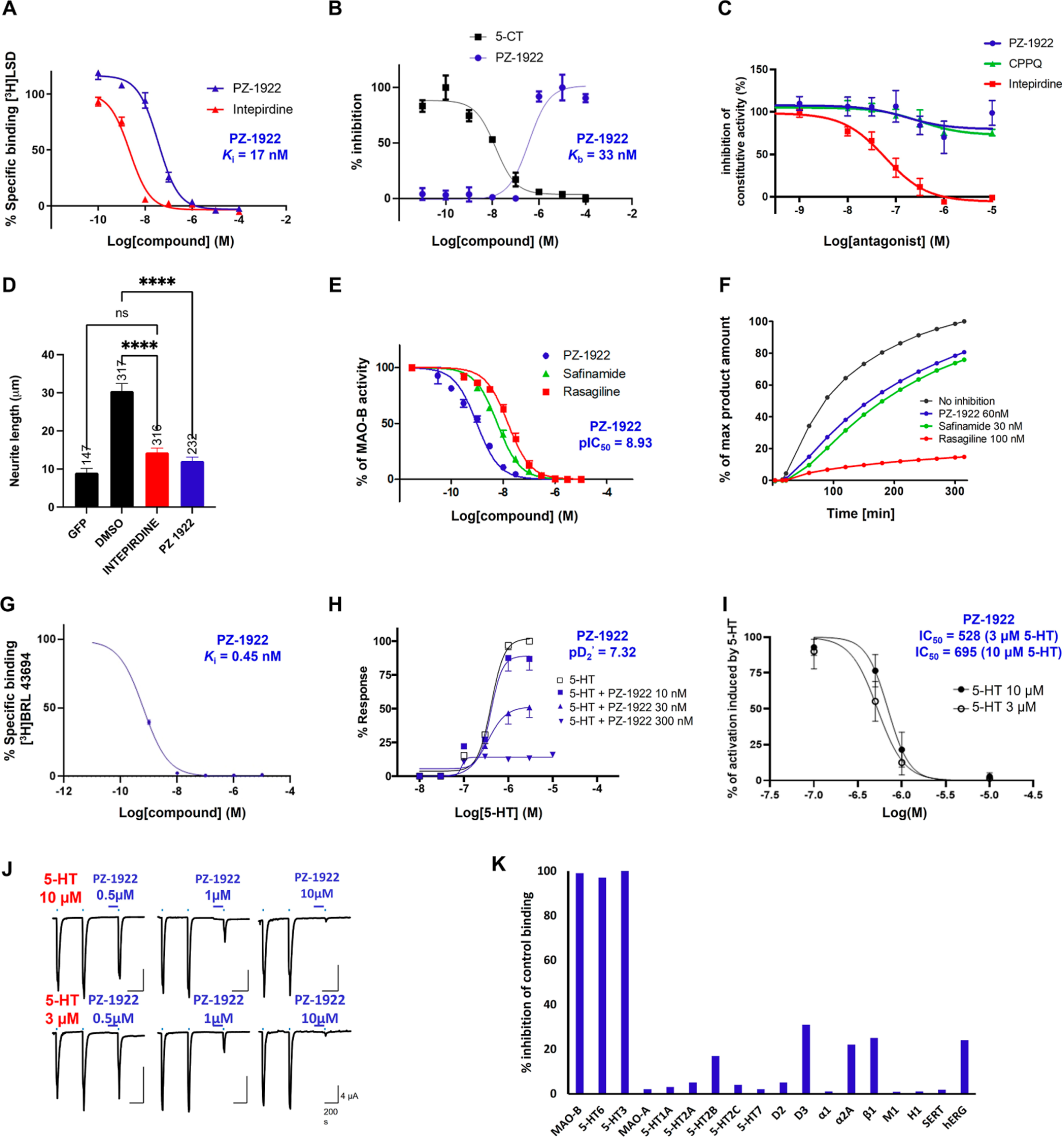
**PZ-1922** behaves as a 5-HT_6_R/5-HT_3_R antagonist and MAO-B inhibitor. (A) **PZ-1922** and intepirdine inhibit binding to 5-HT_6_R using 2 nM [^3^H]-LSD in HEK cells stably expressing *h*5-HT_6_R. *K*_i_ value based on three independent binding experiments (SEM ≤ 15%). (B) **PZ-1922** increases cyclic AMP production elicited by an incremental concentration of 5-carboxamidotryptamine (5-CT) in 1321N1 cells. *K*_b_ value was calculated from the equation *K*_b_ = IC_50_/(1 + *A*/EC_50_) where *A* is the agonist (5-CT) concentration used (1000 nM), IC_50_ is the concentration of antagonist producing a 50% reduction in response to the agonist, and EC_50_ (13 nM) is 5-CT concentration that causes a 50% maximal response. Data are means ± SEM of the values obtained in three independent experiments (SEM ≤ 22%). (C) **PZ-1922**, in contrast to intepirdine, does not impact 5-HT_6_R constitutive activity at Gs signaling in NG108-15 cells transiently expressing the human 5-HT_6_R. Cyclic AMP levels were estimated by using the CAMYEL BRET probe.^22^ Data are the mean ± SEM of the values obtained in three independent experiments performed in quadruplicate using different sets of cultured cells. (D) **PZ-1922** and intepirdine inhibit the Cdk5-induced neurite growth in NG108-15 cells transiently expressing 5-HT_6_R. Cells were exposed to either vehicle, intepirdine (1 μM) or compound **PZ-1922** (1 μM) for 24 h. The histogram shows the means ± SEM of neurite length in each experimental condition measured in three independent experiments. ***p < 0.001 vs cells expressing 5-HT_6_R and treated with DMSO. (E) **PZ-1922** inhibits MAO-B enzyme: inhibitor concentration–enzyme activity curve for **PZ-1922** in the human recombinant MAO-B enzyme assay using the fluorometric method and 200 μM p-tyramine as a substrate. pIC_50_ value calculated from two experiments run in duplicate (SEM < 1%). (F) **PZ-1922** and safinamide behave as reversible MAO-B inhibitors and rasagiline as an irreversible inhibitor of MAO-B used at concentrations corresponding to their IC_80_ values in the presence of the substrate *p*-tyramine. Data present recovery of MAO-B activity after blockade with tested compound and represent means from two experiments performed in duplicate. (G) **PZ-1922** inhibits binding of the radioligand (0.5 nM [^3^H]-BRL 43694) to 5-HT_3_R. *K*_i_ value was based on two independent binding experiments in human recombinant CHO cells expressing *h*5-HT_3_R (SEM ≤ 15%). (H) **PZ-1922** displays antagonist properties at 5-HT_3_R, inhibiting serotonin-induced guinea pig ileum contractility. Responses are expressed as a percentage of maximal serotonin (5-HT) effect (*E*_max_ = 100%), reached in the concentration–response curves obtained before incubation with the tested compound. Each point represents the mean ± SEM (*N* = 4 to 8 trials, SEM ≤ 6.6%). (I) **PZ-1922** inhibits the activity of 5-HT_3_ heterologously expressed in *Xenopus*oocytes. Currents generated by 5-HT_3_ are recorded by the two-electrode voltage-clamp (TEVC) technique. Dots are means ± SEM of 5-HT_3_ normalized activation by 5-HT after preincubation with **PZ-1922**. The number of tested oocytes per condition is between 4 and 8. (J) Representative TEVC recordings. Representative current traces measured for one concentration of **PZ-1922** and one concentration of 5-HT in one oocyte. (K) **PZ-1922** displays selectivity for 15 targets (at 1 μM). A significant response (>50% inhibition) was obtained only for MAO-B, 5-HT_6_R, and 5-HT_3_R. Inhibition assay for MAO-A and binding assays for serotonin 5-HT_1A_R, 5-HT_7_R, and D_2_Rs were performed according to the previously reported methods.^8,17^ Binding experiments for adrenergic α_1_R, α_2A_R, and β_1_R, dopamine D_3_, histaminergic H_1_R, muscarinic M_1_R, serotonin 5-HT_2A_R, 5-HT_2B_R, 5-HT_2C_R, serotonin transporter SERT, and *h*ERG channel were performed at Eurofins, France. For details, see Supporting Information.

Due to its potent and dual activity at 5-HT_6_R and MAO-B (Figure 2A,E), compound **PZ-1922** was selected for further comprehensive evaluation. The high affinity of **PZ-1922** for 5-HT_6_R translated well into potent antagonist properties when tested in 1321N1 cells expressing the human 5-HT_6_R (*K*_b_ = 33 nM, Figure 2B). 5-HT_6_R displays strong constitutive activity at Gs signaling in NG108-15 cells,^19^ defined as spontaneous activity of the receptor in the absence of an agonist. This allows for the pharmacological differentiation of 5-HT_6_R antagonists for 5-HT_6_R inverse agonists, which can inhibit spontaneous cAMP production (may inhibit both agonist-evoked and constitutive activity of the receptor), while neutral antagonists do not affect basal cAMP levels (may inhibit only agonist-evoked activity). In line with the prototypic 5-HT_6_R neutral antagonist CPPQ,^20^ compound **PZ-1922** did not affect basal cAMP levels in NG108-15 cells expressing 5-HT_6_R, classifying it as a neutral antagonist at Gs signaling (Figure 2C). In contrast, the well-characterized 5-HT_6_R inverse agonist intepirdine, used as a control, inhibited basal cAMP production in this model (Figure 2C). Furthermore, the constitutive activity of 5-HT_6_R at Cdk5 signaling can induce neurite growth and differentiation in NG108-15 cells.^21^ In contrast to its neutral antagonist properties at Gs signaling, **PZ-1922** exhibited similar efficacy as intepirdine in reducing neurite length in NG108-15 cells expressing 5-HT_6_Rs, indicating its inverse agonist activity at Cdk5 signaling (Figure 2D).

Similar to safinamide, **PZ-1922** demonstrated the reversible inhibition of MAO-B in kinetic studies performed using a fluorometric method. This reversible inhibition enabled a time-dependent recovery of the enzymatic activity of human recombinant MAO-B (Figure 2F).

**PZ-1922** also displayed a high affinity for human 5-HT_3_R (Figure 2G). It displayed potent antagonist properties at 5-HT_3_R, as evidenced by an ex vivo assay assessing guinea pig ileum contractility (with a p*D*_2_’ (**PZ-1922**) = 7.32, Figure 2H). The 5-HT_3_R antagonist properties of **PZ-1922** were further confirmed through electrophysiological recordings, which indicated its ability to inhibit 5-HT-induced inward currents in *Xenopus* oocytes expressing 5-HT_3_Rs (Figure 2I,J).

**PZ-1922** showed high selectivity over selected GPCRs (such as adrenergic α_2A_, dopamine D_2_, D_3_ receptors, and serotonin 5-HT_1A_, 5-HT_2A_, 5-HT_2C_, and 5-HT_7_, as depicted in Figure 2K) in radioligand binding assays. It did not inhibit the MAO-A enzyme isoform, as assessed by the fluorometric method detecting the activity of human recombinant MAO-A (Figure 2K). Furthermore, **PZ-1922** demonstrated no potential adverse effects related to its affinity for the adrenergic α_1_ receptor, adrenergic β_1_ receptor (which could be associated with hypertension or arrhythmia), D_2_ dopamine receptor (linked to extrapyramidal symptoms and hyperprolactinemia), M_1_ muscarinic receptor, and H_1_ histaminic receptor (which could cause sedation). The cardiac safety of **PZ-1922** was based on its lack of effect on 5-HT_2B_R and the human Ether-à-go-go-Related Gene (*h*ERG) channel, which are indicative of valvulopathy and prolongation of the QT interval, respectively.

### *In Silico* Evaluation of the Binding Mode of **PZ-1922** at 5-HT_6_R and MAO-B

The initial attempt to perform molecular docking of **PZ-1922** with the original crystal structures of 5-HT_6_R^23^ and MAO-B^24^ resulted in inconsistent results. This inconsistency was caused by conformational constraints of the binding site. To overcome these limitations, we used the induced-fit docking procedure. This allowed us to explore more flexible conformational states of the target proteins and obtain a stable form of the ligand complexes. In order to comprehensively analyze the selected SAR outcomes related to **PZ-1922**, we also included previously reported structural congeners from dual 5-HT_6_R/5-HT_3_R antagonists (**PZ-1939**, **FPPQ**),^10^ as well as a class of selective MAO-B inhibitors (**PZ-1771**).^17^

The effect of replacing a sulfonyl linker with a methylene one was examined in 5-HT_6_R comparing compounds **PZ-1939** and **PZ-1922**. Similarly, the replacement of a gem-diF group with a basic center was investigated in MAO-B, comparing compounds **PZ-1771** and **PZ-1922**. To evaluate the stability of the L–R complexes and analyze intermolecular interactions, 100 ns-long MD simulations were performed. The results were illustrated by using the geometrics of the L–R complexes obtained from the most frequently occurring MD trajectories, selected through trajectory clustering.

**Figure 3:**
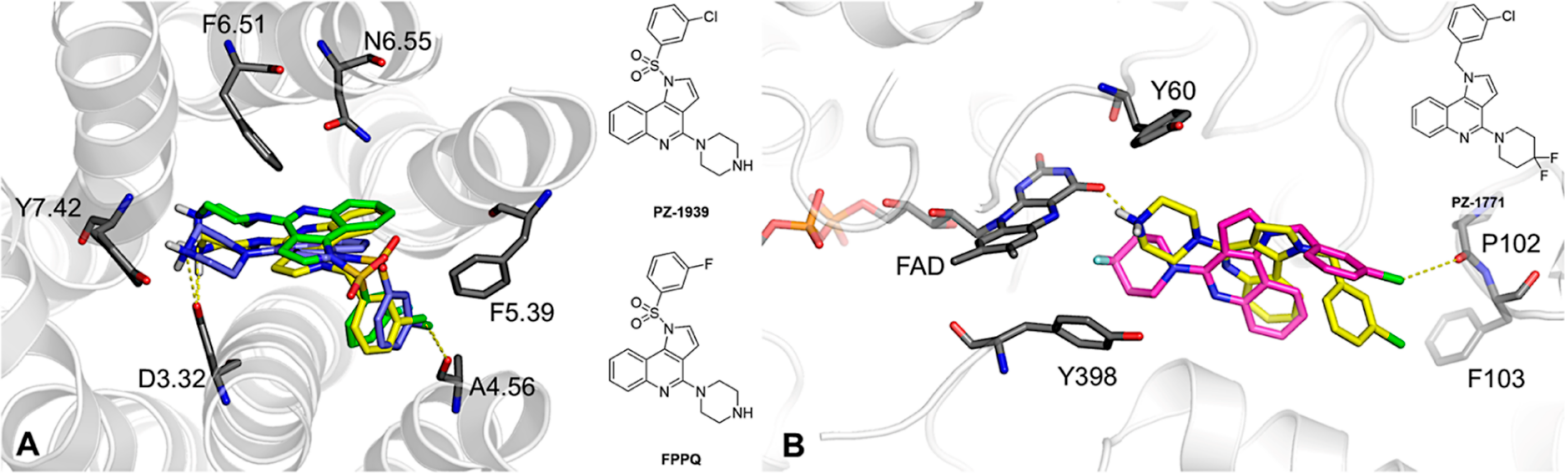
Illustrations of L-R complexes for 5-HT_6_R and MAO-B obtained by 100 ns-long MD simulations. (A) Comparison of binding modes of **PZ-1922** (yellow), **PZ-1939** (blue), and **FPPQ** (green) in 5-HT_6_R (PDB ID: 7XTB). (B) Comparison of binding modes of **PZ-1922** (yellow) and **PZ-1771** (magenta) in the MAO-B catalytic site (PDB ID: 2V5Z). Compounds **PZ-1939** (presented as **18**) and FPPQ were disclosed in ref (10), compound **PZ-1771** refers to compound **26** from ref (17).

Upon analysis of the complexes of **PZ-1922**, **PZ-1939**, and **FPPQ** with 5-HT_6_R, a consistent pattern of L–R interactions at the binding site was observed. This included a salt bridge formation between the protonated piperazine nitrogen and the D3.32 side chain, CH···π interactions with F6.51, CH···π with F5.39, and a halogen bond with the carbonyl oxygen of A4.57 (Figure 3A). Further examination of the MD trajectories for **PZ-1922** and **PZ-1939** showed that the slightly lower affinity of **PZ-1922** could be attributed to a reduced frequency of halogen bond (XB) formation with A4.56, compared to **PZ-1939** (for detailed information, refer to the multivariate plots showing the dependency of the XB distance Cl···O versus the σ-hole angle C–Cl···O for each trajectory frame, Figure S1C). The methylene linker in **PZ-1922** offered greater rotational freedom than the sulfonamide in **PZ-1939**, leading to increased rotatability of the entire benzyl group around the S–C/C–C bond (refer to the C1–N1–C2–C3 dihedral angles in Figure S1B). Additionally, quantum mechanical calculations were performed using a noncovalent interaction (NCI) approach to gain insight into the conformational preferences of **PZ-1922** and **PZ-1939** (Figure S1A). In the structure of **PZ-1922**, only a weak C–H···H attractive NCI was observed. Conversely, in **PZ-1939**, a series of S=O···H–C intramolecular hydrogen bonds between the benzyl and 1*H*-pyrrolo[3,2-*c*]quinoline moieties were observed (Figure S1A). These intramolecular hydrogen bonds in **PZ-1939** restricted its flexibility during MD simulations and positioned the 3-chlorobenzyl group favorably for additional stabilization of the L–R complex by the XB of A4.57.

Regarding MAO-B, **PZ-1922** compared to its analogue lacking a basic center (**PZ-1771**) created an additional charge-assisted hydrogen bond with the cofactor FAD. Furthermore, cation···π interactions occurred between the protonated piperazine fragment and an “aromatic cage” composed of Y435, Y398, Y60, and F343. These crucial interactions properly positioned the ligand for interaction with FAD (Figure 3B).^25,26^ The 3-chlorobenzyl fragment of the ligand was placed near the B entrance channel, and its position was stabilized by weak halogen bonds, with **PZ-1922** forming a halogen bond with the ring centroid of F103, while **PZ-1771** formed a halogen bond with the carbonyl oxygen of P102. It is worth mentioning that the created XBs stabilize the L–R complex (the dihedral angle in both cases is almost at a constant level, Figure S2B), despite the presence of weak intramolecular interactions between the 1*H*-pyrrolo[3,2-*c*]quinoline framework and the 3-chlorobenzyl fragment (as depicted in the NCI plots in Figure S2A, showing only weak C–H···H and/or C–H···C interactions).

### Cryo-EM Structure of **PZ-1922** Bound to 5-HT_3_R

**Figure 4:**
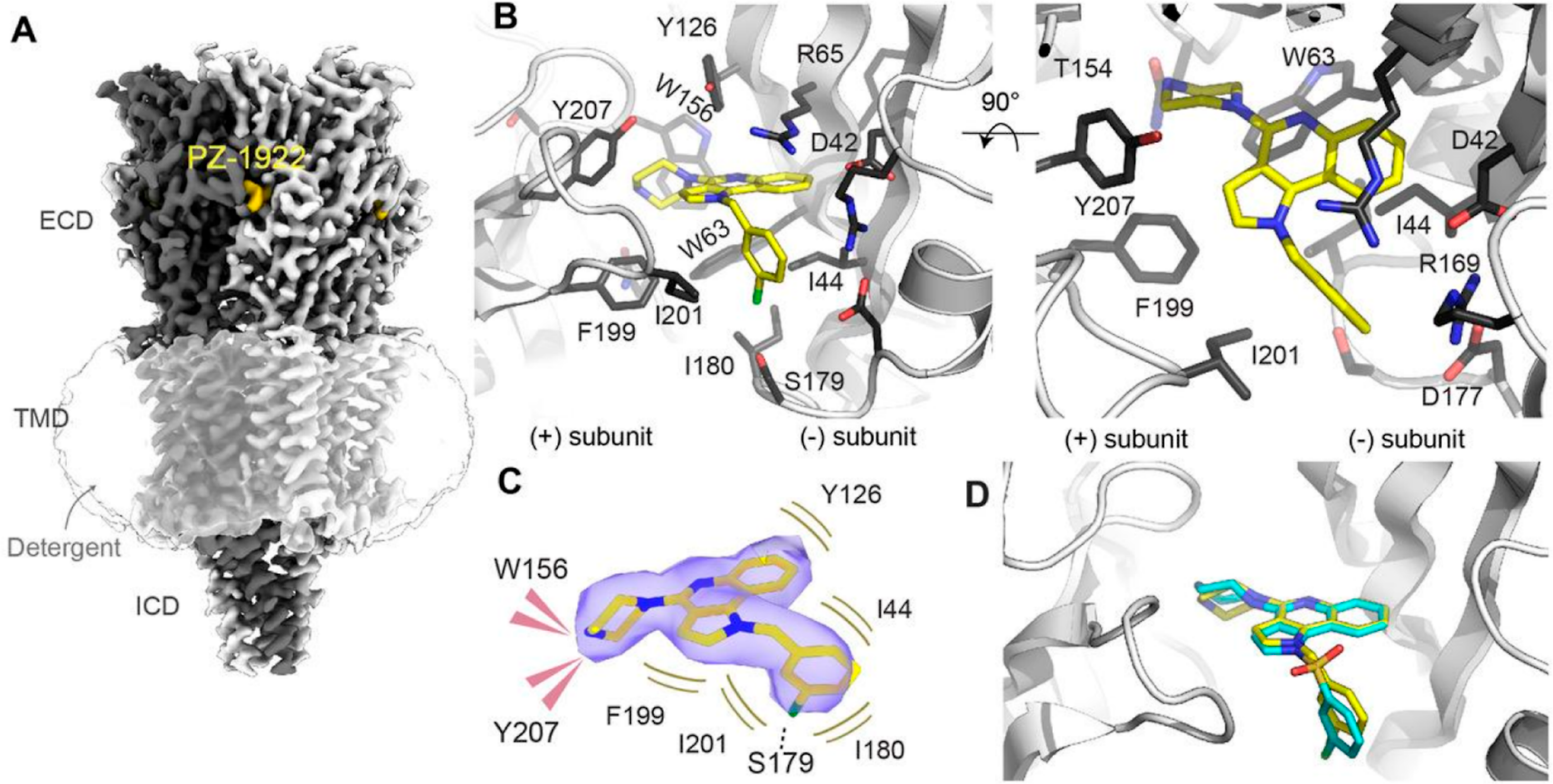
Structure of the mouse 5-HT_3A_ receptor in complex with **PZ-1922**. (A) Cryo-EM representation of the overall structure of the receptor from the perspective of the membrane. (B) Side and top close-up views of the binding site, with **PZ-1922** (PDB ID: 8CC6; EMDB ID: EMD-16555) in yellow sticks, and residues within 4.5 Å of it are depicted as sticks. In the top view, loop E side chains are removed for clarity. (C) Quality of the Coulombic potential map around **PZ-1922** and schematic of the interaction between the drug and the receptor. (D) Superimposition with **PZ-1939** (PDB ID: 8CC7; EMDB ID: EMD-16557). The drug positions are extremely similar, but in the case of **PZ-1939** the density corresponding to the outer ring is not well-defined.

Purified mouse 5-HT_3A_R was subjected to cryo-EM imaging in the presence of 10 mM **PZ-1922** (Figure S3 and Table S1). A reconstruction of the receptor at 3.2 Å was obtained, revealing an extra density corresponding to the ligand within the binding site. The structure possesses the typical receptor features: a pentamer is organized around a central pseudosymmetrical axis that coincides with the ion pathway. The binding sites were located in the extracellular domain, specifically in the clefts between subunits (Figure 4A). The transmembrane domain contained the ion pore, which featured a closed hydrophobic gate (data not shown). The intracellular domain, which harbors an intrinsically disordered stretch, is partially resolved. The overall conformation of the receptor closely resembled previously determined inhibited states observed in the presence of other classes of antagonists (e.g., RMSD of 0.7 for Calphas with the structure bound to the antiemetic drug palonosetron).^27,28^

The binding pose of **PZ-1922** is in good agreement with the known determinants of 5-HT_3_R antagonism (Figure S3). The piperazine moiety of the ligand is located at the back of the binding cavity, surrounded by a cluster of aromatic residues (F199, Y207, W156 and W63, as depicted in Figures 4B and 4C). Notably, these aromatic residues are also conserved in the human 5-HT_3_R. The piperazine group is appropriately positioned to engage in a characteristic cation–π interaction with W156. The central 1*H*-pyrrolo[3,2-*c*]quinoline group is stabilized by hydrophobic interactions with F199, Y207, Y126, R75, I44, and W63. Furthermore, the chlorobenzyl fragment of the ligand resides at the entrance of the binding cavity, establishing hydrophobic interactions and potentially forming an H-bond with S179.

To compare the binding of two closely related compounds, we also performed cryo-EM imaging of m5-HT_3_R in the presence of 10 mM **PZ-1939**, resulting in a reconstruction with a resolution of 3.0 Å (Figure S4). The overall structure of the receptor and the binding pose of the ligand closely resemble those observed with **PZ-1922** (Figure 4D). However, it is worth noting that the density corresponding to the most outward cycle of **PZ-1939** appears fragmented, indicating a certain degree of flexibility (Figures S4 and S5). This observation can be attributed to the bulkier O=S=O linker in **PZ-1939**, which may hinder the complete capping of loop C (residues 199–207) and permit this flexibility. Furthermore, this finding supports the notion that the chlorobenzyl cycle of **PZ-1922** plays a secondary role in its inhibitory effect on 5-HT_3_R (Figure S6).

### **PZ-1922** is Brain Penetrant and Shows Favorable Bioavailability

**Figure 5:**
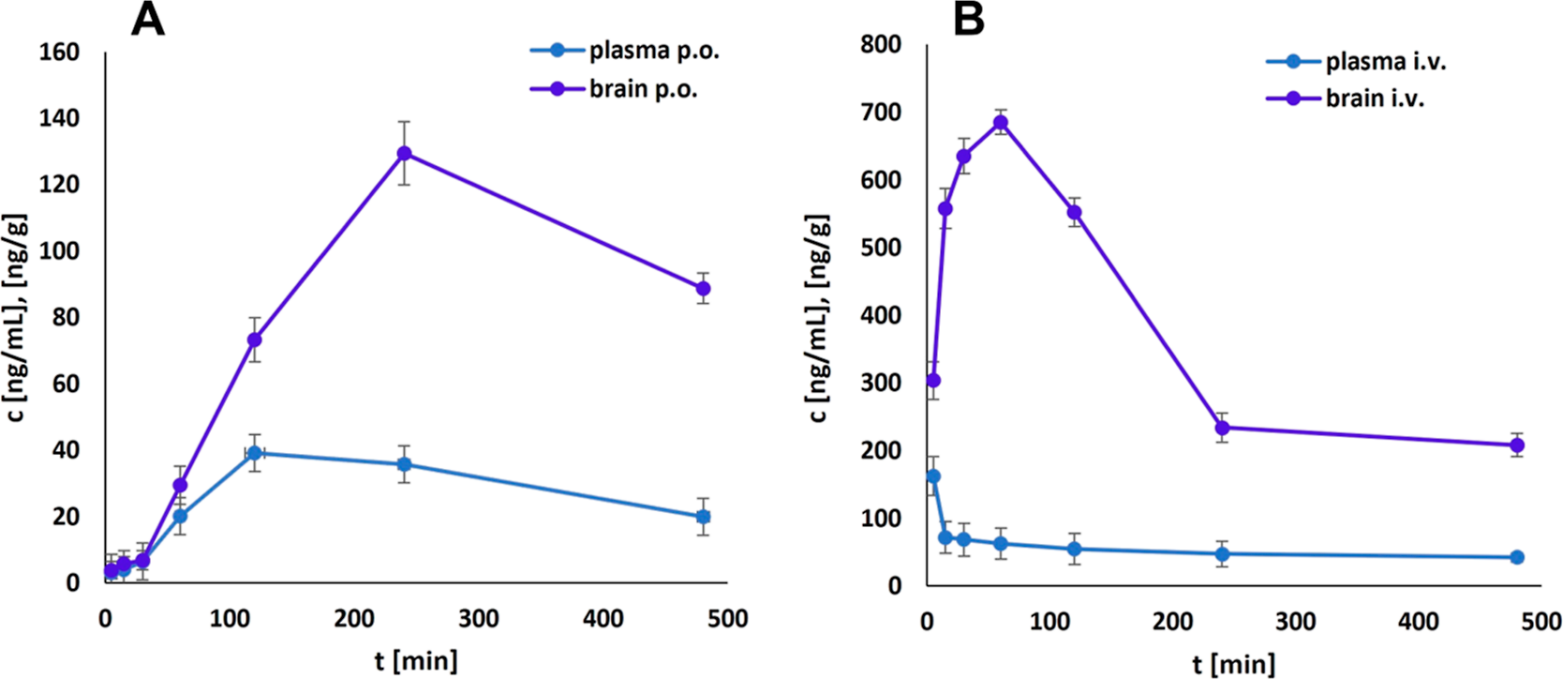
Concentration–time profiles for **PZ-1922** in plasma and brain after intragastric (A) and intravenous (B) administration to Wistar rats (*N* = 6 per animal group) at a dose of 3 mg/kg. Concentrations of **PZ-1922** in plasma and brain are expressed in nanograms per milliliter and nanogram per gram (±SD), respectively.

To investigate the preliminary pharmacokinetic characteristics of **PZ-1922**, its concentrations in plasma and brain were determined in male Wistar rats at various time points following oral (intragastric, *ig* administration, Figure 5 and Table S2) and intravenous (*iv*, Figure 5 and Table S2) administration at a dose of 3 mg/kg. **PZ-1922** demonstrated the ability to penetrate the blood–brain barrier (BBB), as evidenced by a brain concentration (*C*_max_) of 129.6 ± 9.49 ng/g reached 4 h (*T*_max_) after *ig* administration. The compound exhibited a decent half-life of approximately 6 h after *ig* administration and 13 h after *iv* administration. Notably, **PZ-1922** displayed a high volume of distribution (*ca*. 42 L/kg), indicating high penetration throughout the body. Furthermore, it exhibited significant BBB penetration, as indicated by brain-to-plasma ratios of 3.14 (*ig*) and 6.10 (*iv*). The compound was characterized by high bioavailability (*F* = 48%) after *ig* administration. Overall, the study indicated that **PZ-1922** possesses favorable pharmacokinetic properties for *in vivo* pharmacology studies.

### **PZ-1922** Reverses Scopolamine-Induced Cognitive Impairment in the NOR Test

**Figure 6:**
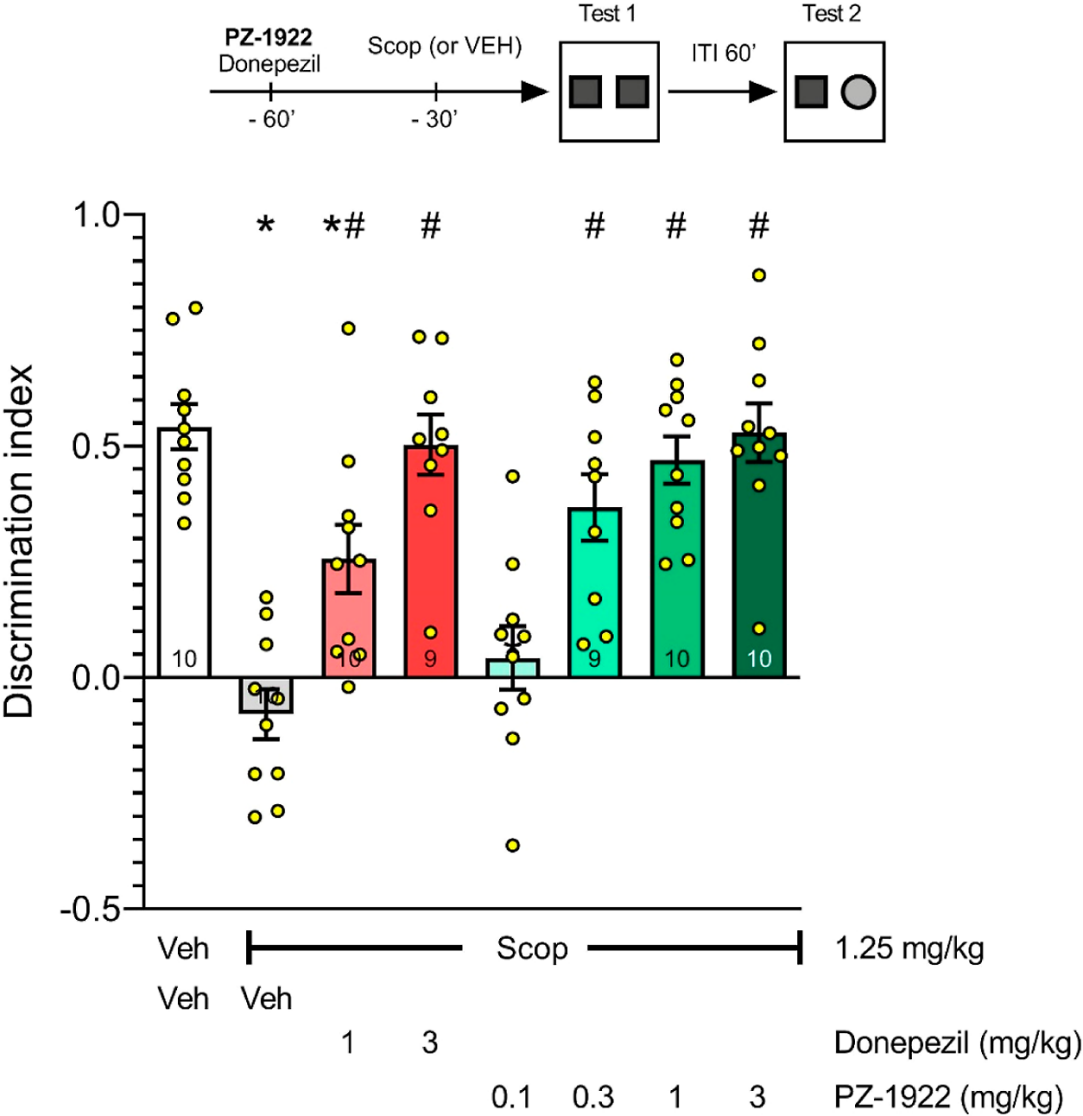
**PZ-1922** and a “positive” control donepezil prevent SCOP-induced cognitive impairment in a NOR test in rats. At the recognition trial, vehicle-treated, but not SCOP-treated, rats spent significantly more time exploring novel objects compared with the familiar one, indicating that the administration of SCOP (1.25 mg/kg, *ip*) abolished the ability to recognize objects. The effect of SCOP was prevented in a dose-related manner by the *ip* administration of donepezil and **PZ-1922**. Data are presented as the mean ± SEM of the DIs. *N* = 9–10 animals per group, shown in detail at the bottom of bars. Symbols *P < 0.05 represent significant reduction in DI compared with the vehicle-treated group; #*P* < 0.05, significant increase in DI as compared with vehicle/scopolamine-treated group (Tukey’s post hoc test following one-way ANOVA: *F*(7,70) = 14.50; *P* < 0.0001).

Administration of SCOP, a muscarinic receptor antagonist, results in both short- and long-term memory impairment in the NOR test, which is often used to examine cognitive decline caused by alteration of cholinergic transmission. In the NOR test, when **PZ-1922** was administered at varying doses (0.3, 1, and 3 mg/kg) 30 min prior to SCOP administration, it dose-dependently prevented the SCOP-induced deficits in short-term memory. Similarly, donepezil, an AChE inhibitor commonly used as a reference drug, also reduced the memory deficits induced by SCOP when administered at doses of 1 and 3 mg/kg (Figure 6).

### **PZ-1922**, Used as a Curative and Preventive Treatment, Abolishes Aβ-Induced Memory Deficits in the T-Maze Test

**Figure 7:**
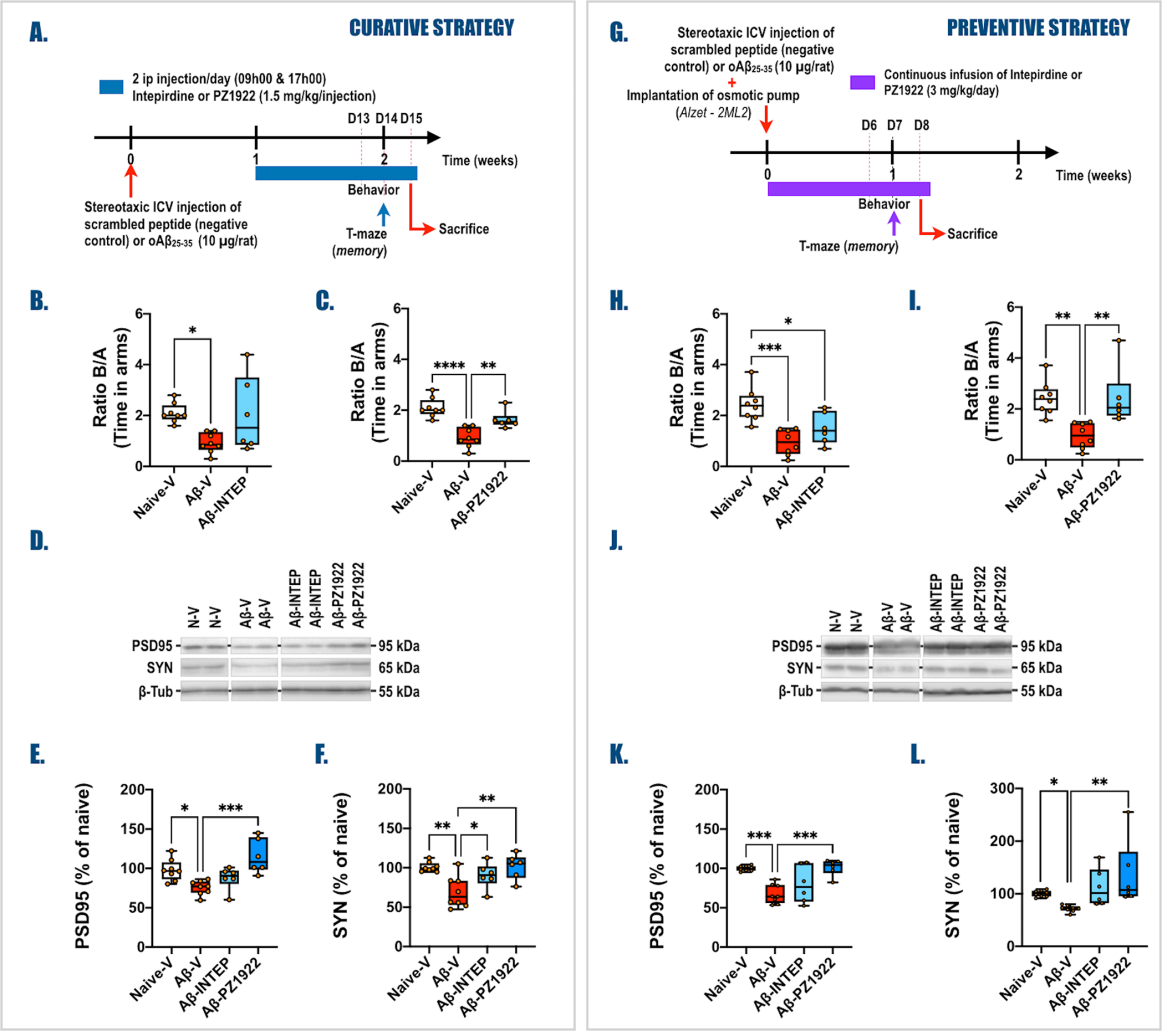
Impact of curative and preventive strategies using **PZ-1922** (in comparison with intepirdine) on oAβ_25–35_-induced behavioral and synaptic alterations. (A) Curative experimental protocol—At T0, adult male rats (Sprague–Dawley) received any *icv* injection (Naive-V groups), an *icv* injection of oAβ_25–35_ (10 μg/rat, Aβ groups), or an *icv* injection of Scrambled control peptide (Scr groups). One week later, animals were subjected to treatment twice a day for 7 days (09:00 and 17:00) with an intraperitoneal (*ip*) injection of either vehicle (NaCl 0.9%, V groups), intepirdine (1.5 mg/kg/*ip* injection, INTEP groups), or **PZ-1922** (1.5 mg/kg/*ip* injection, **PZ-1922** groups). One day before the sacrifice (day 14), the spatial short-term memory of each rat was tested in the T-maze test. On the morning of day 15, the animals were sacrificed, and hippocampi were rapidly collected for Western blot analysis. (B,C) Spatial short-term memory performance was determined in the T-maze test after intepirdine (B) and **PZ-1922** (C) treatments and expressed as the ratio of the time spent in the initially closed arm (B) to the time spent in the previous arm (A). (D–F) Variations of postsynaptic (PSD-95, 95 kDa, D,E) and presynaptic (SYN, 65 kDa, D,F) markers in the whole hippocampus were evaluated by Western blot in each group. (G) Preventive experimental protocol. At T0, adult male rats (Sprague–Dawley) received any *icv* injection (Naive groups), an *icv* injection of oAβ_25–35_ (10 μg/rat, Aβ groups), or an *icv* injection of Scrambled peptide (Scr groups). In the same chirurgical procedure, osmotic pumps (Alzet, EML2) were implanted in intraperitoneal (*ip*), and either vehicle (NaCl 0.9%, V groups) or intepirdine (3 mg/kg/day, INTEP groups) or **PZ-1922** (3 mg/kg/day, **PZ-1922** groups) was delivered continuously during 1 week. One day before the sacrifice (day 7), the spatial short-term memory of each rat was tested in the T-maze test. The following day (day 8), the animals were sacrificed between 9:00 and 12:00, and hippocampi were rapidly collected for Western blot analysis. (H,I) Spatial short-term memory performance was determined in the T-maze test after intepirdine (H) and **PZ-1922** (I) treatments and was expressed as the ratio of the time spent in the initially closed arm (B) over the time spent in the previous arm (A). (J–L) Expression of postsynaptic (PSD-95, 95 kDa, J,K) and presynaptic (SYN, 65 kDa, J,L) markers in the whole hippocampus were evaluated by Western blot in each group. All Western blot data were normalized to the respective variations of β-tubulin (β-tub, 55 kDa) and expressed in percent of values obtained in the noninjected Naive group. To simplify data, the Naive-V, Aβ-V, Aβ-INTEP, and Aβ-**PZ-1922** groups were represented. Data for all other groups are available as Supporting Information (Figures S8 and S11). All data are presented as box and whiskers with Min to Max and Median *n* = 8 for Naive-V and Aβ-V groups; and *n* = 6 for Aβ-INTEP and Aβ-**PZ-1922** groups. One-way ANOVA followed by a Tukey’s or Dunnett’s multiple comparison was performed (Table S4). *p < 0.05; **p < 0.01; ***p < 0.001; and ****p < 0.0001 vs selected group.

Accumulation of Aβ oligomers in AD leads to synaptic disruption and progressive neuronal cell death. In AD patients, Aβ oligomers primarily consist of Aβ_1–40_ and Aβ_1–42_ peptides,^29^ along with shorter sequences such as Aβ_25–35_ or Aβ_25–35/40_, resulting from enzymatic cleavage of Aβ_1–40._^30,31^ These shorter peptides contain extracellular and transmembrane residues, including a biologically active region of Aβ^32,33^ and a highly hydrophobic region that facilitates the formation of stable aggregates. Notably, the undecapeptide Aβ_25–35_, which exhibits better solubility and higher efficacy, induces neurite atrophy, neuronal cell death, synaptic loss, as well as synaptic plasticity and memory deficits, similarly to Aβ_1–40_ and Aβ_1–42_.^34^ To investigate the impact of **PZ-1922** and intepirdine on Aβ-induced memory deficits, we performed intracerebroventricular (*icv*) injection of a solution containing Aβ_25–35_ oligomers (oAβ_25–35_) in rats.^35,36^ Subsequently, we examined the effects of **PZ-1922** and intepirdine on these memory deficits (Figure 7).

In the curative approach, the tested compounds (intepirdine and **PZ-1922**) were administered 1 week after the induction of amyloid toxicity (*icv* injection of oAβ_25–35_). The administration involved two *ip* injections per day for 1 week, with each injection containing 1.5 mg/kg of the respective compound (Figure 7A). Fourteen days after the oAβ_25–35_ injection, a behavioral memory test (T-maze) was performed (Figure 7A).

In the preventive approach, the tested compounds were administered simultaneously with the induction of Aβ toxicity. This involved the *icv* injection of oAβ_25–35_ and concurrent implantation of an osmotic pump (Alzet-EML2, Charles-River, France). The osmotic pump continuously delivered the compounds at a dose of 3 mg/kg/day for 1 week (Figure 7G). One week after the beginning of the treatment (day 7), the animals underwent the T-maze test.

The administration of **PZ-1922** 1 week after the oAβ_25–35_ injections effectively reversed the memory deficits induced by amyloid toxicity in the T-maze test, demonstrating its curative effect (Figures 7C and S8B). On the other hand, intepirdine only partially reversed the memory deficits in this experimental paradigm (Figures 7B and S8A). Similarly, when administered concomitantly with oAβ_25–35_, **PZ-1922** (Figure 7G) prevented the emergence of cognitive deficits, while intepirdine did not exhibit the same preventive effect (Figures 7H,I and S11B).

### **PZ-1922** Impacts the Level of AD Biomarkers Associated with Synaptic Integrity, Cdk5 Activity, and Neuroinflammation

Having established the beneficial effect of a triple inhibitory approach targeting 5-HT_6_/5-HT_3_/MAO-B on SCOP-induced and Aβ-induced memory decline, our next step was to evaluate the impact of **PZ-1922** and intepirdine on biomarkers associated with synaptic integrity (postsynaptic density protein 95—PSD-95, synaptotagmin—SYN), apoptosis (caspase type 3—Casp 3), Cdk5 activity (Cdk5, p22/p35), and neuroinflammation (glial fibrillary acidic protein—GFAP, ionized calcium-binding adaptor molecule 1—Iba1) in the hippocampus of rats injected with oAβ_25–35_. We assessed these biomarkers by performing Western blotting analysis on proteins extracted from the hippocampus of animals treated with curative and preventive approaches involving **PZ-1922** and intepirdine.

Injection of oAβ_25–35_ produced alterations in hippocampal synapses, as evidenced by a decrease in PSD-95 and SYN levels (Figure 7D–F,J–L). **PZ-1922** reversed PSD-95 and SYN levels when administered as a curative or preventive treatment (Figures 7D–F,I–K, S8D,F, and S11D,F). In contrast, intepirdine reversed only the SYN level in the curative experimental paradigm (Figures 7D,F, S8C,E, and S11C,E).

The synaptic effects of oAβ_25–35_ are also accompanied by cell death and progressive apoptosis, as indicated by an increased expression of Caspase-3 and Pro-caspase-3.^37,38^ Both **PZ-1922** and intepirdine decreased expression of Caspase-3 and Pro-caspase-3 when employed in a curative strategy (Figures S7A,C and S10E,H), and prevented increased expression of Caspase-3 and Pro-caspase-3 when administered as a preventive treatment (Figures S7D,F and S13E–I,H).

Further analysis focused on the effect of **PZ-1922** and intepirdine on Cdk5 expression in the hippocampus of Aβ-injected rats. Apart from its coupling to 5-HT_6_R, which enables the regulation of neurite growth,^21,39,40^ Cdk5 activity is dependent on its association with the neuron-specific activators p35 and p39, as well as their truncated forms p25 and p29. Aberrant overactivation of Cdk5 occurs through the conversion of p35 to p25, leading to the formation of the Cdk5-p25 complex. This complex hyperphosphorylates Tau protein and indirectly increases amyloid production by stimulating BACE1.

**Figure 8:**
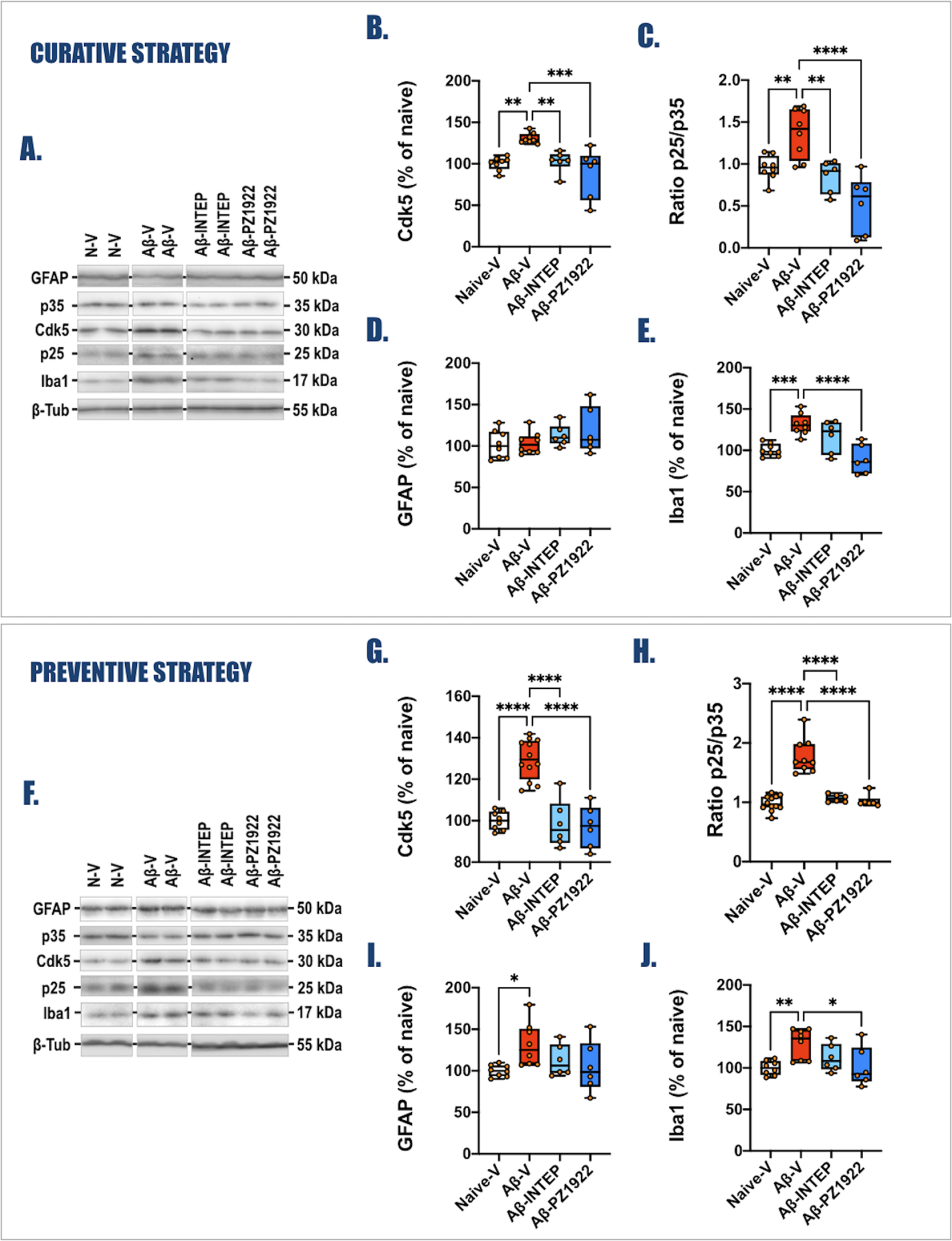
Impact of the curative and preventive treatments with intepirdine and **PZ-1922** on specific AD markers induced by the *icv* injection of oAβ_25–35_ was evaluated by Western blot analysis of hippocampal proteins. Variations of Cdk5 activity (Cdk5, 30 kDa; and the ratio of p25/p35, 25/35 kDa) (panels (A–C) for curative strategy and (F–H) for preventive strategy) and neuroinflammation (GFAP, 50 kDa; and Iba1, 17 kDa) (panels (A,D,E) for curative strategy and (F,I,J) for preventive strategy) were evaluated in each group, normalized using β-tubulin expression (β-tub, 55 kDa). Results are expressed as the percentage of variations compared to noninjected rats (Naive group). For experimental protocol, see Figure 6A (Curative) and 6G (Preventive). To simplify data, only the Naive-V, Aβ-V, Aβ-INTEP, and Aβ-**PZ-1922** groups were represented. Data for all other groups are available as Supporting Information (Figures S8–S10 and S11–S13). All data are presented as box and whiskers with Min to Max and Median with *n* = 8 for Naive-V and Aβ-V groups; and *n* = 6 for Aβ-INTEP and Aβ-**PZ-1922** groups. One-way ANOVA followed by Dunnett’s multiple comparison was performed (Table S4). *p < 0.05; ***p < 0.001, and ****p < 0.0001 vs oAβ_25–35_ group treated with vehicle (Aβ-V).

The *icv* injection of oAβ_25–35_ induced an increase in Cdk5 expression (Figure 8A,B,F,G) and an elevated conversion of p35 to p25 (Figure 8A,C,F,H). Both **PZ-1922** and intepirdine reduced the level of Cdk5 overexpression and the level of p25 protein and p25/p35 ratio in curative treatment (Figures 8 and S9). Moreover, **PZ-1922** and intepirdine decreased the Cdk5 overexpression, the level of p25 protein, and p25/p35 ratio and restored the level of p35 protein when applied in preventive treatment approach (Figures 8 and S12).

Since astrocyte activation and recruitment of microglia contribute to the neuroinflammatory processes, we next assessed the hippocampal GFAP and Iba1. As previously reported,^37,38,41^ we observed a transient activation of astrocytes only 1 week after the *icv* injection of oAβ_25–35_, as demonstrated by the increased level of GFAP in the preventive approach (Figures 8A,D,F,I, S10A,B, S13A,B). This was followed by a sustained recruitment of microglial cells (Figures 8A,E,F,J, S10C,D, and S13C,D). It is worth noting that GFAP expression was no longer elevated 2 weeks after the *icv* injection of oAβ_25–35_ in the curative approach. Neither **PZ-1922** nor intepirdine impacted the GFAP increase (Figure 8D,I). In contrast to intepirdine, **PZ-1922** decreased the Iba1 level regardless of the applied approach (Figure 8E,J).

## Discussion and Conclusions

The emerging consensus regarding the multifactorial nature of AD has prompted a shift in small-molecule drug discovery strategies from single-target to multitarget approaches. In this study, we considered the combination of 5-HT_6_R/5-HT_3_R antagonism and inhibition of MAO-B as a promising approach to address the complex processes underlying cognition and degeneration in AD.

The rationale for this approach originated from behavioral studies revealing that antagonism at 5-HT_6_R alleviates cognitive deficits through the corticolimbic release of acetylcholine and glutamate achieved by blocking 5-HT_6_Rs located on GABAergic neurons. Moreover, ondansetron, a 5-HT_3_R antagonist, has already displayed pro-cognitive properties in clinical studies, and the neuroprotective effects of 5-HT_3_R antagonists found in *in vitro* and *in vivo* experiments further supported the inclusion of 5-HT_3_R antagonism in our multitarget approach. Considering the beneficial effects of lazabemide (a reversible MAO-B inhibitor) in a phase III clinical trial for AD, and the potential of reversible MAO-B inhibition to attenuate astrogliosis and enhance synaptic transmission,^16^ reversible MAO-B inhibition holds promise for modulating neurodegenerative and neuroinflammatory processes in AD.

We thus explored the concept of the merged ligands to identify a novel compound, 1-(3-chlorobenzyl)-4-(piperazine-1-yl)-1*H*-pyrrolo[3,2-*c*]quinoline (**PZ-1922**), as a potential treatment for AD (Figure 1). **PZ-1922** exhibited antagonistic activity at both 5-HT_6_R and 5-HT_3_R, as well as potent reversible inhibition of MAO-B. In contrast to the 5-HT_6_R inverse agonist intepirdine, it exhibited neutral antagonistic properties at 5-HT_6_R-mediated Gs signaling and behaved as an inverse agonist at Cdk5 signaling induced by constitutively active 5-HT_6_Rs (Figure 2).

Molecular dynamic simulations of **PZ-1922**, along with its dual-acting 5-HT_6_R/5-HT_3_R antagonist (**PZ-1939**) and selective MAO-B inhibitor (**PZ-1771**), revealed structural determinants that allowed simultaneous 5-HT_6_R antagonism and MAO-B inhibition. Cryo-EM confirmed the fragments required for interaction with 5-HT_3_R. Subsequent experiments using full-length mouse 5-HT_3_R demonstrated that the binding structure of **PZ-1922** resolved at 3.2 Å resembled that of palonosetron.

The progressive memory decline characterizing AD is associated with the loss of cholinergic neurons and synapses and a decrease in ACh levels in brain regions involved in cognitive functions. To study the impact of altered cholinergic transmission on cognitive decline, we examined the compound’s efficacy in reversing scopolamine-induced amnesia and demonstrated that **PZ-1922** mimicked the pro-cognitive effects of clinically used donepezil in the NOR test (Figure 6).

In the Aβ-induced memory deficit model in rats, which involved *icv* injection of oAβ_25–35_, **PZ-1922** exhibited pro-cognitive properties in both curative and preventive paradigms. Importantly, in the latter model, **PZ-1922** demonstrated superior efficacy compared to interpirdine, which acts solely as a 5-HT_6_R antagonist. These data suggest that the combined antagonism at 5-HT_6_R and 5-HT_3_R, along with the inhibitory activity at MAO-B, enhances the effectiveness of 5-HT_6_R antagonism alone in mitigating Aβ-induced memory deficits.

As previously reported,^38,41^ behavioral changes induced by oAβ_25–35_ were accompanied by alterations in hippocampal synapses, as evidenced by a decrease in PSD-95 and SYN levels (Figure 7D–F,J–L). When administered as a curative or preventive treatment, **PZ-1922** effectively reversed and prevented both postsynaptic and presynaptic deficits (Figure 7D,E,J,K). In contrast, intepirdine showed efficacy only at the presynaptic level and solely in the curative experimental paradigm (Figure 7D,F). These observations are in line with the results of the behavioral studies, indicating that compound **PZ-1922** displays both curative and preventive effects, while intepirdine is only partially effective in the curative approach.

Multiple pieces of evidence support the correlation between Cdk5 overactivity and the pathogenesis of AD. Consistent with previous data,^41,42^ the *icv* injection of oAβ_25–35_ induced an increase in Cdk5 expression (Figure 8A,B,F,G) and an elevated conversion of p35 to p25 (Figure 8A,C,F,H), ultimately leading to Cdk5 overactivation. Our findings demonstrate that both **PZ-1922** and intepirdine effectively blocked Cdk5 activation in the hippocampus, regardless of the preventive or curative approach (Figures 8, S9, and S12).

The progression of AD is also associated with sustained neuroinflammation resulting from the activation of astrocytes, known as “reactive astrogliosis”, and the recruitment of microglia. Markers of microglia and astrocytes are elevated in AD patients and colocalized with Aβ plaques.^37,38,41,43^ Therefore, we examined the levels of the reactive astrocyte marker GFAP and the microglial marker Iba1 in the hippocampus. **PZ-1922**, but not intepirdine, effectively abolished the neuroinflammatory response triggered by the administration of oAβ_25–35_ in both the curative and preventive approaches (Figures 8, S10, and S13).

In conclusion, we demonstrate that simultaneous blockade of 5-HT_6_R and 5-HT_3_R, along with inhibitory activity at MAO-B, enhances the effectiveness of 5-HT_6_R antagonism alone in the alleviation of cognitive deficits in rats. The present results further confirm the superiority of **PZ-1922** over intepirdine (a 5-HT_6_R antagonist) in preventing and alleviating the molecular and synaptic alterations, as well as modulating neuroinflammatory processes in the hippocampus of Aβ-injected rats. The properties of **PZ-1922** highlight the promising potential of simultaneously targeting 5-HT_6_R, 5-HT_3_R, and MAO-B as a novel approach for the development of anti-AD agents.

## Experimental Section

### Synthesis General Information

The synthesis was conducted at room temperature unless indicated otherwise. Organic solvents (from Sigma-Aldrich and Chempur) were reagent grade and used without purification. All reagents (Sigma-Aldrich, Fluorochem, and TCI) were of the highest purity. Column chromatography was performed on silica gel Merck 60 (70–230 mesh ASTM).

UPLC analyses and high-resolution mass spectra (LC-HRMS) were obtained on a Waters ACQUITY I-Class PLUS SYNAPT XS high-esolution mass spectrometer (Waters, Milford, CT, USA) with the MS-Q-TOF detector and UV–VIS-DAD eλ detector. The ACQUITY UPLC BEH C18, 1.7 μm (2.1 × 100 mm) column was used with the VanGuard Acquity UPLC BEH C18, 1.7 μm (2.1 × 5 mm) (Waters, Milford, CT, USA). Standard solutions (1 mg/mL) of each compound were prepared in analytical grade MeCN/water mixture (1:1; v/v). Conditions applied were as follows: eluent A (water/0.1% HCOOH), eluent B (MeCN/0.1% HCOOH), a flow rate of 0.3 mL/min, a gradient of 5–100% B over 13 min, and an injection volume of 1 μL. The UPLC/MS purity of all test compounds and key intermediates was determined to be >95%.

Elemental analysis for C, H, N, and S was performed on the elemental Vario EI III Elemental Analyzer (Hanau, Germany). All values are reported as percentages and were within ±0.4% of the calculated values.

^1^H NMR and ^13^C NMR spectra were recorded using JEOL JNM-ECZR 500 RS1 (ECZR version) at 500 and 126 MHz, respectively, and are reported in ppm using deuterated solvent for calibration (CDCl_3_, methanol-*d*_4_ or dmso-*d*_6_). The *J* values are given in Hertz (Hz). Melting points were determined with a Büchi apparatus and are uncorrected.

Spectral data for the final compounds **3**, **4**, **6**, **7**–**9**, **11–15**, **17–19**, **21**, **22**, and **24**–**26** are included in the Supporting Information.

#### General Procedure for Preparation of Compounds **3**–**12**

Respective compound **2a–2e** (0.52 mmol, 1 equiv) was mixed together with Pd_2_(dba)_3_ (10 mg, 0.010 mmol, 0.02 equiv), BINAP (13 mg, 0.021 mmol, 0.04 equiv), and *t*-BuONa (70 mg, 0.73 mmol, 1.4 equiv). The solids were suspended in 4 mL of a mixture of dioxane and *t*-BuOH (3/1, v/v), and the respective primary amine (1.2 equiv) was added. The reaction was irradiated with microwaves at 90 °C for 1 h under argon atmosphere. The resulting mixture was filtrated through a pad of Celite, concentrated, and purified by column chromatography on silica gel using CH_2_Cl_2_/MeOH as a developing solvent. The obtained product was converted into HCl salt of secondary amine upon overnight treatment with 1 M methanolic solution of HCl and subsequent filtration.

#### (*S*)-1-(3-Chlorobenzyl)-*N*-(pyrrolidin-3-yl)-1*H*-pyrrolo[3,2-*c*]quinolin-4-amine Hydrochloride **5**

White solid, overall yield 60%, *t*_R_ = 4.47 min, mp 181–183 °C, C_22_H_22_Cl_2_N_4_, MW 413.35. ^1^H NMR (500 MHz, methanol-*d*_4_): δ ppm 2.34–2.48 (m, 1H), 2.58–2.75 (m, 1H), 3.50–3.58 (m, 1H), 3.63–3.74 (m, 2H), 3.84 (dd, *J* = 12.6, 6.8 Hz, 1H), 5.15–5.28 (m, 1H), 5.91 (s, 2H), 6.97–7.04 (m, 2H), 7.22–7.34 (m, 2H), 7.40 (ddd, *J* = 8.3, 7.2, 1.2 Hz, 1H), 7.53–7.64 (m, 3H), 8.01 (d, *J* = 8.2 Hz, 1H), and 8.18 (d, *J* = 8.2 Hz, 1H). ^13^C NMR (126 MHz, methanol-*d*_4_): δ ppm 31.90, 45.75, 50.69, 52.98, 53.64, 105.77, 111.75, 115.30, 120.16, 123.02, 125.48, 126.72, 126.98, 129.14, 130.02, 131.75, 133.22, 135.30, 135.39, 136.14, 140.17, and 149.84. Monoisotopic mass 376.15, [M + H]^+^ = 377.2. HRMS calcd for C_22_H_22_ClN_4_, 377.1533; found, 377.1489.

#### (*S*)-1-(3-Chlorobenzyl)-*N*-(pyrrolidin-2-ylmethyl)-1*H*-pyrrolo[3,2-*c*]quinolin-4-amine Hydrochloride **10**

White solid, overall yield 52%, *t*_R_ = 4.52 min, mp 172–174 °C, C_23_H_24_Cl_2_N_4_, MW 427.37. ^1^H NMR (500 MHz, methanol-*d*_4_): δ ppm 1.92–1.99 (m, 1H), 2.07–2.15 (m, 1H), 2.17–2.25 (m, 1H), 2.37–2.46 (m, 1H), 3.35–3.43 (m, 1H), 3.45–3.53 (m, 1H), 4.10–4.20 (m, 2H), 4.21–4.28 (m, 1H), 5.83 (s, 2H), 6.96–7.00 (m, 2H), 7.19–7.30 (m, 2H), 7.35 (t, *J* = 7.7 Hz, 1H), 7.44 (d, *J* = 3.1 Hz, 1H), 7.52–7.57 (m, 2H), 7.90 (d, 1H), and 8.20 (d, *J* = 8.3 Hz, 1H). ^13^C NMR (126 MHz, methanol-*d*_4_): δ ppm 24.28, 29.07, 44.40, 46.73, 53.64, 60.33, 105.52, 111.70, 115.16, 120.19, 122.89, 125.49, 126.63, 126.96, 129.11, 129.90, 131.73, 133.23, 135.09, 135.19, 136.07, 140.10, and 150.26. Monoisotopic mass 390.16, [M + H]^+^ = 391.1. HRMS calcd for C_23_H_24_ClN_4_, 391.1689, found, 391.1672.

#### General Procedure for Preparation of Compounds **13**–**21**

Compound **2c** (170 mg, 0.52 mmol, 1 equiv) was suspended in acetonitrile (12 mL), followed by addition of TEA (0.290 mL, 2.08 mmol, 4 equiv). Next, the respective secondary amine (1.56 mmol, 3 equiv) was added. The reaction was heated in a microwave reactor at 140 °C for 7 h. The solvent was evaporated, and the crude products were purified using CH_2_Cl_2_/MeOH (compounds: **13**–**15**) or ethyl acetate (AcOEt)/hexane (Hex) (compounds **16**–**21**) as the developing solvent. The obtained product was converted into HCl salt of secondary amine upon overnight treatment with 1 M methanolic solution of HCl and subsequent filtration.

#### 1-(3-Chlorobenzyl)-4-(piperazin-1-yl)-1*H*-pyrrolo[3,2-*c*]quinoline Hydrochloride **(16) PZ-1922**

White solid, overall yield 55%, *t*_R_ = 4.29 min, mp 144–146 °C, C_22_H_24_Cl_2_N_4_O, Anal. Calcd for C_22_H_22_Cl_2_N_4_ × H_2_O: C, 61.26; H, 5.61; N, 12.99. Found: C, 61.27; H, 5.82; N, 12.81. MW 431.36. ^1^H NMR (500 MHz, methanol-*d*_4_): δ ppm 3.60–3.65 (m, 4H), 4.36–4.42 (m, 4H), 6.00 (s, 2H), 7.03–7.07 (m, 2H), 7.26–7.35 (m, 3H), 7.47 (ddd, *J* = 8.4, 7.2, 1.1 Hz, 1H), 7.67 (ddd, *J* = 8.4, 7.2, 1.2 Hz, 1H), 7.73 (d, *J* = 3.4 Hz, 1H), and 8.10–8.14 (m, 2H). ^13^C NMR (126 MHz, methanol-*d*_4_): δ ppm 44.32, 47.64, 53.97, 107.56, 112.52, 115.33, 120.34, 123.22, 125.61, 127.03, 127.26, 129.24, 130.68, 131.83, 133.96, 135.23, 136.19, 137.97, 139.86, and 151.96. Monoisotopic mass 376.15, [M + H]^+^ = 377.2. HRMS calcd for C_22_H_22_ClN_4_, 377.1533; found, 377.1565.

#### 1-(3-Chlorobenzyl)-4-(2,6-diazaspiro[3.4]octan-2-yl)-1*H*-pyrrolo[3,2-*c*]quinoline Hydrochloride **20**

White solid, overall yield 46%, *t*_R_ = 4.57 min, mp 125–127 °C, C_24_H_24_Cl_2_N_4_, MW 439.38. ^1^H NMR (500 MHz, dmso-*d*_6_): δ 2.32 (t, *J* = 7.5 Hz, 2H), 3.19–3.26 (m, 2H), 3.44–3.56 (m, 2H), 4.74 (dd, *J* = 106.4, 44.0 Hz, 4H), 5.97 (s, 2H), 6.87 *J* = 6.95 (m, 2H), 7.05 (s, 1H), 7.27–7.36 (m, 3H), 7.53 (t, *J* = 7.8 Hz, 1H), 7.77 (d, *J* = 3.1 Hz, 1H), 7.96 (d, *J* = 8.3 Hz, 1H), and 8.19 (d, *J* = 8.4 Hz, 1H). ^13^C NMR (126 MHz, methanol-*d*_4_): δ ppm 36.29, 42.22, 45.92, 53.71, 54.34, 106.16, 110.62, 114.80, 119.24, 123.19, 125.50, 126.14, 126.94, 129.15, 130.22, 131.76, 133.35, 135.61, 135.63, 136.15, 140.11, and 149.78. Monoisotopic mass: 402.16, [M + H]^+^ = 403.2. HRMS calcd for C_24_H_24_ClN_4_, 403.1689; found, 403.1701.

#### 1-(3-Chlorobenzyl)-4-(piperidin-4-yl)-1*H*-pyrrolo[3,2-*c*]quinoline Hydrochloride **23**

Compound **22** (70 mg, 0.17 mmol, 1 equiv) was dissolved in 3 mL of MeOH and 10% Pd/C (25 mg, 30% weight) was subsequently added. The mixture was stirred under a hydrogen atmosphere at room temperature for 2 h. Then, the mixture was filtered through Celite, which was rinsed three times with MeOH. The solvent was evaporated, and the obtained product was converted into HCl salt of secondary amine upon overnight treatment with 1 M methanolic solution of HCl and subsequent filtration.

White solid, overall yield 47%, *t*_R_ = 4.32 min, mp 180–182 °C, C_23_H_23_Cl_2_N_3_, MW 412.36. ^1^H NMR (500 MHz, methanol-*d*_4_): δ ppm 2.38 (d, *J* = 13.8 Hz, 2H), 2.68–2.80 (m, 2H), 3.39 (td, *J* = 12.6, 11.9, 2.9 Hz, 2H), 3.69 (d, *J* = 13.1 Hz, 2H), 4.04–4.15 (m, 1H), 6.12 (s, 2H), 7.00–7.07 (m, 1H), 7.12–7.14 (m, 1H), 7.27–7.34 (m, 2H), 7.68 (d, *J* = 3.4 Hz, 1H), 7.75 (t, *J* = 8.3, 7.1, 1.1 Hz, 1H), 7.89 (t, *J* = 8.4, 7.1, 1.2 Hz, 1H), 7.94 (d, *J* = 3.4 Hz, 1H), 8.38 (dd, *J* = 8.6, 1.2 Hz, 1H), and 8.49 (d, *J* = 8.5 Hz, 1H). ^13^C NMR (126 MHz, methanol-*d*_4_): δ ppm 28.07, 40.02, 44.93, 53.95, 106.99, 117.63, 121.02, 122.35, 123.62, 125.62, 127.21, 129.39, 129.80, 131.40, 131.90, 136.25, 136.48, 138.54, 139.47, and 157.14. Monoisotopic mass, 375.15; [M + H]^+^ = 376.2. HRMS calcd for C_23_H_23_ClN_3_, 376.1580; found, 376.1552.

### *In Silico* Evaluation

#### Structures of the Receptors

The structure of 5-HT_6_R in the complex with agonist serotonin (PDB ID: 7XTB) and MAO-B cocrystallized with the inhibitor safinamide (PDB ID: 2V5Z) were retrieved from the Protein Data Bank.

#### Molecular Docking

The three-dimensional structures of the ligands were prepared using LigPrep v3.6,^44^ and the appropriate ionization states at pH = 7.4 ± 1.0 were assigned using Epik v3.4.^45^ The Protein Preparation Wizard was used to assign the bond orders and appropriate amino acid ionization states and to check for steric clashes. The receptor grid was generated (OPLS3e force field)^46^ by centering the grid box with a size of 10 Å on the cocrystallized ligand. Automated flexible docking was performed using Glide v6.9^47^ at the SP level, and eight poses per ligand were generated.

#### Molecular Dynamics

100 ns-long molecular dynamics (MD) simulations were performed using Schrödinger Desmond software.^48^ Each ligand–receptor complex was immersed into a POPC (309.5 K) membrane bilayer, whose position was calculated using the PPM web server (accessed 20 January 2023).^49^ The system was solvated by water molecules described by the TIP4P potential, and the OPLS3 force field was used for all atoms. 0.15 M NaCl was added to mimic the ionic strength inside the cell. The output trajectories were hierarchically clustered into 5 groups according to the ligand using the trajectory analysis tool from Schrödinger Suite.

#### Quantum Chemical Calculations

To recognize intramolecular interactions within the **PZ-1939**, **PZ-1771**, and **PZ-1922** and structural frameworks, the NCIPLOT program was used.^50^ First, the density functional theory (DFT) calculations for ligand conformations determined from the MD trajectory (the most populated cluster) with the GAUSSIAN16 package^51^ at the B3LYP-D3/cc-pVDZ level^52−55^ with the PCM (water) were performed.^56^ Wave functions were obtained and further used to generate reduced electron density gradient (RDG) surfaces by using the NCIPLOT program. The NCI (noncovalent interaction) analysis is based on the reduced RDG defined as
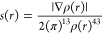
where ∇ρ(*r*) corresponds to a gradient of the electron density. The NCI allows for visualization of both attractive and repulsive interaction regions. To calculate the energy of detected intramolecular interactions, the topological analysis of electron density was carried out in the AIMAll program.^57^ The energy of the noncovalent intramolecular bonds detected in the analyzed structures was calculated using the Espinosa equation^58^ as follows
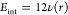
where *E*_int_ is the energy of the interatomic interaction, and *v*(*r*) is the kinetic energy at the bond critical point (BCP).

### Cryo-Electron Microscopy

The mouse 5-HT_3A_ receptor expression, purification, and cryo-EM imaging were performed as previously described with some minor modifications. Briefly, the receptor was produced using a stable and inducible HEK293 cell line. The membranes of harvested cells were solubilized in the detergent C12E9 (Anatrace), and the receptor was purified by affinity purification followed by size-exclusion chromatography. The fractions corresponding to the pentameric receptors were pooled and concentrated to 10 mg/mL. The purified receptor was incubated with the ligand (10 mmol of **PZ-1922** or **PZ-1939**) for 5 min, then 3.0 μL were deposited on glow-discharged (25 mA, 45 s) Copper/Rhodium Quantifoil R1.2/1.3 grids. Grids were blotted (8 s, 100% humidity, 4 °C) and plunged frozen. The best-looking among a set of 6 grids (in terms of particle density and apparent ice thickness) was selected for data collection (see Table S1) on the IBS Glacios microscope equipped with a Gatan K2 Summit camera. Data treatment was done with CryoSPARC, except for the picking step that was performed with crYOLO. After particles were extracted, several rounds of 2D classification, ab initio model reconstruction with 3 classes, and heterogeneous refinement were performed. The best-looking class was subjected to nonuniform refinement. An existing structure of the 5-HT_3A_ receptor was rigidly fitted in the 3D reconstruction. The ligands structures, generated within Phenix Elbow, were added in the binding site, and the model was iteratively refined with Isolde, Coot, and Phenix.

### *In Vitro* Pharmacological Evaluation

#### 5-HT_6_R Affinity Evaluation

##### Cell Culture for Radioligand Binding Assays

HEK293 cells stably expressing human 5-HT_6_R were grown at 37 °C in a humidified atmosphere with 5% CO_2_ and grown in Dulbecco’s modified Eagle medium (DMEM) containing 10% dialyzed fetal bovine serum and 500 μg/mL G418 sulfate. For membrane preparation, cells were subcultured in 150 cm^2^ flasks, grown to 90% confluence, washed twice with phosphate buffered saline (PBS) prewarmed to 37 °C, and pelleted by centrifugation (200*g*) in PBS containing 0.1 mM EDTA and 1 mM dithiothreitol. Prior to membrane preparation, pellets were stored at −80 °C.

##### Radioligand Binding Assay

The cell pellets were thawed and homogenized in 10 volumes of assay buffer using an Ultra Turrax tissue homogenizer, centrifuged twice at 35,000*g* for 15 min at 4 °C, and incubated for 15 min at 37 °C between centrifugation rounds. The composition of the assay buffer was 50 mM Tris–HCl, 0.5 mM EDTA, and 4 mM MgCl_2_. The assays were performed in a total volume of 200 μL in 96-well microtiter plates for 1 h at 37 °C. The process of equilibration was terminated by rapid filtration through Unifilter plates with a 96-well cell harvester, and radioactivity retained on the filters was quantified on a Microbeta plate reader (PerkinElmer, USA). For displacement studies, the assay samples contained 2 nM [^3^H]-LSD (83.6 Ci/mmol, PerkinElmer). Nonspecific binding was defined with 10 μM methiothepine. Each compound was tested at incremental concentrations (10^–10^ to 10^–4^ M) in triplicate. The inhibition constants (*K*_i_) were calculated from the Cheng–Prusoff equation.^59^ Results were expressed as the means of results obtained in two independent experiments.

##### Evaluation of 5-HT_6_R Antagonist Activity of **PZ-1922** at Gs Signaling

Compounds were examined for their ability to inhibit 5-HT_6_R-operated cAMP production in the presence of 100 nM (EC_80_) 5-carboxamidotryptamine (5-CT). The level of cAMP was measured in 1321N1 cells expressing *h*5-HT_6_R (PerkinElmer, #ES-316-CF). Total cAMP was measured using the LANCE cAMP detection kit according to the manufacturer’s instructions (PerkinElmer, #TRF0263). Cells were incubated with a mixture of compounds for 30 min at room temperature (RT) in a white polystyrene OptiPlate-384 (PerkinElmer, #6007299) microplate. After incubation, the cells were lysed by the addition of 10 μL of cAMP detection buffer, including Eu-cAMP tracer and ULight-anti-cAMP working solution. The plate was incubated at RT for 1 h before measuring the signal with a Tecan multimode plate reader (Infinite M1000 Pro). Compounds were tested in triplicate at eight concentrations in the range from 10^–11^ to 10^–4^ M. *K*_b_ constants were calculated from Cheng–Prusoff equation^59^ adapted to functional assays.

##### Determination of 5-HT_6_R Constitutive Activity at Gs Signaling

cAMP measurement was performed in NG108-15 cells transiently expressing 5-HT_6_R using the Bioluminescence Resonance Energy Transfer (BRET) sensor for cAMP, CAMYEL (cAMP sensor using YFP-Epac-RLuc).^22^ NG108-15 cells were cotransfected in suspension with 5-HT_6_R (or empty vector for Mock condition) and CAMYEL constructs, using Lipofectamine 2000, according to the manufacturer’s protocol, and plated in white 96-well plates (Greiner), at a density of 80,000 cells per well. 24 h after transfection, cells were washed with PBS containing calcium and magnesium. Coelanterazine H (Molecular Probes) was added at a final concentration of 5 μM and left at room temperature for 5 min. BRET was measured using a Mithras LB 940 plate reader (Berthold Technologies).

##### Impact of Compounds on Neurite Growth

NG108-15 cells were grown in DMEM supplemented with 10% dialyzed fetal calf serum, 2% hypoxanthine/aminopterin/thymidine (Life technologies), and antibiotics. Cells were transfected with plasmids encoding either cytosolic GFP or GFP-tagged 5-HT_6_R in suspension using Lipofectamine 2000 (Life technologies) and plated on glass coverslips. Six hours after transfection, cells were treated with either DMSO (Vehicle), **PZ-1922** (1 μM), or intepirdine (1 μM) for 24 h. Cells were fixed in 4% paraformaldehyde (PFA) supplemented with 4% sucrose for 10 min. PFA fluorescence was quenched by incubating the cells in PBS containing 0.1 M glycine prior to mounting in Prolong Gold antifade reagent (Thermo Fisher Scientific). Cells were imaged using an AxioImager Z1 microscope equipped with epifluorescence (Zeiss), using a 20× objective for cultured cells, and neurite length (index of 5-HT_6_R constitutive activity as Cdk5 signaling) was assessed using the Neuron J plugin of the ImageJ software (NIH).

##### Monoamine Oxidase Assays

Inhibition activity of the evaluated compounds was measured using human recombinant MAO-B and MAO-A (Sigma-Aldrich M7441 and M7316) in the fluorometric method for detecting monoamine oxidase activity. The assay was carried out in a 96-well plate. Two μL of the appropriate concentrations of tested compounds in DMSO were added to wells that contained 98 μL of enzyme dilution (0.53 U/mL) in phosphate buffer (50 mM, pH 7.4). After 30 min of preincubation at room temperature, 50 μL of the solution of 800 μM 10-Acetyl-3,7 dihydroxyphenoxazine (Cayman Chemical Company 10010469) and 4 U/mL horse radish peroxidase (HRP, Sigma-Aldrich P6782) were added, and enzymatic reaction was started by the addition of 50 μL of 800 μM *p*-tyramine (Alfa Aesar A12220) solution. The signal was measured after 1 h (excitation at 570 nm and emission at 585 nm) using an EnSpire multimode plate reader (PerkinElmer, Inc.). Rasagiline (1 μM) or clorgyline (1 μM) were tested as reference compounds for MAO-B and MAO-A inhibitions, respectively.^60,61^

##### MAO-B Reversibility Studies

To investigate the reversibility of MAO-B inhibition, compound **PZ-1922**, rasagiline, and safinamide were tested in concentrations corresponding to their IC_80_ values. The experiment was carried out in a 96-well plate. *h*MAO-B was incubated with inhibitors for 30 min, then low concentration of *p*-tyramine (10 μM in wells) and a solution of 10-acetyl-3,7-dihydroxyphenoxazine and HRP (200 μM and 1 U/mL in wells) were added to the plate. The fluorescence signal had been measured in a microplate reader for 22 min, then the concentration of *p*-tyramine was increased to 1 mM. After the addition of *p*-tyramine, fluorescence was measured every 5 min for 5 h in order to monitor the enzymatic reaction product formation.^60−62^

### Ex Vivo Evaluation of Antagonistic Properties of **PZ-1922** at 5-HT_3_R

Isolated guinea pig ileum was employed to test the affinity for 5-HT_3_Rs and the intrinsic activity of the investigated compound. The tissue was dissected from male guinea pigs previously deprived of food for 24 h but with free access to drinking water. A 15 cm ileum segment was excised from the small intestine of male guinea pigs and immersed into a Krebs solution (NaCl 120 mM, KCl 5.6 mM, MgCl_2_ 2.2 mM, CaCl_2_ 2.4 mM, NaHCO_3_ 19 mM, glucose 10 mM, pH 7.4). After the first 5 cm length closest to the ileocecal junction had been discarded, 2 cm long fragments were cut. Each segment of the ileum was placed in a 20 mL chamber of the tissue organ bath system (Tissue Organ Bath System-750 TOBS, DMT, Denmark) filled with the Krebs solution at 37 °C, pH 7.4, with constant oxygenation (O_2_/CO_2_, 19:1), fixed by the lower end to a rod and by the upper end to the force–displacement transducer. The preparation was allowed to stabilize in organ baths for 60 min under a resting tension of 0.5 g, washing every 15 min with fresh Krebs solution. After the equilibration period, a cumulative concentration–response curve was constructed in each tissue for 5-HT (10 nM to 10 μM) by the method of van Rossum.^63^ The inhibitory effect of compounds was first evaluated by their influence (after 15 min of incubation with the tissue) on the contraction induced by a single administration of 5-HT at the concentration of 3 μM and expressed as a percentage of inhibition of the maximal tension obtained with the contractile agent. **PZ-1922** was then tested by using an additional method. After establishment of the first 5-HT concentration–response curve, washing out the tissue, and stabilization period, the same tissues were subsequently incubated with one of the concentrations of the tested compound for 15 min, and the next cumulative concentration curve for 5-HT was obtained. Only one concentration of the studied compound was tested on each piece of tissue. Concentration–response curves were analyzed using GraphPad Prism 5.0 (GraphPad Software Inc., San Diego, CA, USA), and the antagonistic properties were expressed as p*D*_2_’ (p*D*_2_’—negative logarithm of the molar concentration of antagonist which reduces the effect of an agonist to 50% its maximum).

### Electrophysiological Evaluation of Antagonist Properties of **PZ-1922** at 5-HT_3_R

m5HT_3_R was heterologously expressed in *Xenopus* oocytes by microinjection of 2 ng of phenol/chloroform purified mRNA obtained by *in vitro* transcription (mMESSAGE mMACHINE T7 kit, ThermoFisher). Oocytes were surgically removed from anesthetized *Xenopus* females using procedures that conformed to European regulations for animal handling and experiments and were approved by governmental services (authorization APAFIS# 30915-2021040615209331 granted to Christophe Moreau by the Ministère de l’enseignement supérieur, de la recherche et de l’innovation, on 07 April 2021, valid until 6 April 2026).

Two-electrode voltage-clamp recordings have been performed with the Hi-Clamp automate (Multi-Channel Systems). The running buffer was modified from the standard ND96 buffer by removing calcium ions and adding 1 mM EGTA to avoid the activation of endogenous calcium-activated chloride channels. The composition of the buffer was 91 mM Na, 99 mM Cl^–^, 2 mM KCl, 1.8 mM Ca^2+^, 1 mM Mg^2+^, 5 mM HEPES, pH7.4. Two borosilicate pipets were filled with 3 M KCl. Two 15 s incubations of 5-HT were performed followed by 5 min washing in buffer flow before 2 min incubation with **PZ-1922**. Amplitude of inhibitions was determined by a third 15 s incubation with 5-HT immediately following **PZ-1922** application. 5-HT and **PZ-1922** solution changes were performed by moving the impaled oocytes in continuously stirred 200 mL reservoirs of a 96-well plate. Washings were performed in the wash-station under continuous flow of the running buffer for 5 min. Data were collected in excel files with homemade software by Michel Vivaudou and statistically analyzed with the Prism 8 software (Graphpad). Current amplitudes were normalized from responses after 2 min of incubation in buffer (in the absence of **PZ-1922**).

### *In Vivo* Pharmacokinetic and Pharmacological Properties of Compound **PZ-1922**

#### Animals for Pharmacokinetic Assessment, NOR, and T-Maze Test

A group of 64 adult male Wistar rats (220–250 g) were used for assessment of the pharmacokinetic profile of **PZ-1922**. The animals were purchased from the Animal House at the Faculty of Pharmacy, Jagiellonian University Medical College, Krakow, Poland. During the habituation period, groups of 4 rats were kept in a plastic cage at a controlled room temperature (22 ± 2 °C), humidity (55 ± 10%), and full spectrum cold white light (350–400 lx) on 12 h light/12 h dark cycles (the lights on at 7:00 a.m. and off at 19:00 p.m.) and had free access to standard laboratory pellet and tap water. For the pharmacokinetic study, **PZ-1922** dissolved in water for injection was administered intravenously (*iv*) and intragastrically (*ig*) at a dose of 3 mg/kg. Blood samples were collected at 0 (before injection) and after injection at 5, 15, 30, 60, 120, 240, and 480 min. Blood and brain samples were collected under general anesthesia induced by *ip* injection of 50 mg/kg ketamine plus 8 mg/kg xylazine. Blood samples were taken after animal decapitation into heparinized tubes and immediately centrifuged at 1000*g* for 10 min, and plasma was collected. The plasma and brain samples were immediately frozen at −80 °C. All experimental procedures were carried out in accordance with EU Directive 2010/63/EU and approved by the I Local Ethics Committee for Experiments on Animals of the Jagiellonian University in Krakow, Poland (no. 347/2019).

For the NOR test, male Sprague–Dawley rats (Charles River, Germany) weighing ∼250 g at the arrival were housed in the standard laboratory cages, under standard colony A/C controlled conditions: room temperature 21 ± 2 °C, humidity (40–50%), 12 h light/dark cycle (lights on: 06:00) with *ad libitum* access to food and water. Rats were allowed to acclimate for at least 7 days before the beginning of the experimental procedure. During this week, the animals were handled at least 3 times. Behavioral testing was carried out during the light phase of the light/dark cycle. The experiments were conducted in accordance with the NIH Guide for the Care and Use of Laboratory Animals and were approved by the Ethics Committee for Animal Experiments, Maj Institute of Pharmacology.

For the AD model, adult male Sprague–Dawley rats (Janvier Lab., France) weighing 260–280 g (8 weeks) at the beginning of the experiments were housed 1 week before experiments in a standard animal facility of the University of Montpellier (CECEMA, registration number E34-172-23) (12/12 h light/dark cycle with lights on at 07:00; 21 ± 1 °C, food and water ad libitum). All experiments, including sacrifices, were performed in conscious rats between 09:00 and 12:00, during the diurnal period of the circadian rhythm. Male has been preferred over female because the latter may present a confounding factor of the estrous cycle, a parameter that needs to be systematically controlled. Animal procedures were conducted in strict adherence to the European Union Directive of 2010 (2010/63/EU). The National French Animal Welfare Committee and the local committee at the University of Montpellier approved all protocols (authorization: CEEA-LR-12160). All efforts were made to minimize the number of animals used, potential pain, suffering, and distress.

### Preliminary Pharmacokinetics Evaluation of **PZ-1922**

#### Materials and Reagents

HPLC grade methanol and acetonitrile and reagent grade formic acid, hydrochloric acid, potassium dihydrogen phosphate, orthophosphoric acid, and sodium chloride were purchased from Merck (Darmstadt, Germany).

Control blood and brain samples were obtained from adult male Wistar rats. Rats were anesthetized by intraperitoneal injection of 50 mg/kg ketamine plus 8 mg/kg xylazine, and blood samples were collected in heparinized tubes after animal decapitation. The plasma was separated by centrifugation (1000*g*, 10 min). Plasma and brain were stored at −80 °C pending analysis.

#### Sample Treatment

The plasma and brain sample pretreatment procedures involved acetonitrile precipitation. A 10 μL aliquot of the internal standard (IS, PH002437, Merck, Darmstadt, Germany) working solution (5 μg/mL) was added to 100 μL of the collected mice plasma and brain samples, which were then vortex-mixed for 10 s. Thereafter, 200 μL of acetonitrile was added, vortexed during 20 min, and then centrifuged (7840*g*, 10 min). The supernatant (200 μL) was then transferred to an insert placed in an autosampler vial, and a 10 μL volume of this was injected onto the analytical column.

Brain samples were thawed before use, and whole brains were weighted and placed in a glass mortar and pestle tissue grinder and homogenized with an appropriate amount of phosphate buffer (pH 7.4) in a 1:5 ratio. Afterward, 100 μL of tissue homogenates was transferred to new Eppendorf tubes and spiked with 10 μL of the internal standard working solution. All samples were stored on ice during the preparation process, followed by procedures similar to those described above.

#### Pharmacokinetic Study

Pharmacokinetic parameters were calculated by a noncompartmental approach from the average concentration values, using Phoenix WinNonlin software (Certara, Princeton, NJ 08540 USA). The first-order elimination rate constant (λz) was calculated by linear regression of the log concentration versus time. The area under the mean plasma and brain concentration versus time curve (AUC_0→t_) was calculated from zero to the last concentration point using the linear trapezoidal rule. The detailed calculations are presented in Supporting Information.

### Behavioral and Biochemical Impact of **PZ-1922**

#### Scopolamine-Induced Amnesia in NOR Test in Rats

##### NOR Procedure

At least 1 h before the beginning of the experiments, rats were transferred to the experimental room for acclimation. Rats were tested in a dimly lit (25 lx) “open field” apparatus made of dull gray plastic (66 × 56 × 30 cm). After each measurement, the floor was cleaned and dried.

The procedure consisted of habituation to the arena (without any objects) for 5 min, 24 h before the test, which comprised two trials separated by an inter-trial interval (ITI).

For the scopolamine-induced memory impairment paradigm, 1 h ITI was chosen. During the first trial (familiarization T1), two identical objects (A1 and A2) were presented in opposite corners, approximately 10 cm from the walls of the open field. In the second trial (recognition, T2), one of the objects was replaced by a novel one (A = familiar and B = novel). Both trials lasted 3 min, and animals were returned to their home cage after T1. The objects used were glass beakers filled with gravel and plastic bottles filled with sand. The heights of the objects were comparable (∼12 cm), and the objects were heavy enough that they could not be displaced by the animals. The sequence of presentations and the location of the objects were randomly assigned to each rat. The animals explored the objects by looking, licking, sniffing, or touching the object while sniffing but not when leaning against, standing, or sitting on the object. Any rat spending less than 5 s exploring the two objects within 3 min of T1 or T2 was eliminated from the study. Exploration time of the objects and the distance traveled were measured using the Any-maze video tracking system. Based on exploration time (*E*) of two objects during T2, the discrimination index (DI) was calculated according to the formula DI = (EB – EA)/(EA + AB).

Scopolamine, used to attenuate learning, was administered at a dose of 1.25 mg/kg (*ip*) 30 min before the familiarization phase (T1). The compounds **PZ-1922** (0.1; 0.3; 1 and 3 mg/kg) and donepezil (1 and 3 mg/kg) were administrated *ip* 60 min before the familiarization phase (T1).

##### Drugs

Scopolamine hydrobromide (Sigma-Aldrich, Germany), donepezil hydrochloride (Abcam, United Kingdom), or **PZ-1922** was diluted in distilled water.

#### Aβ-Induced Toxicity in T-Maze Test in Rats

##### Amyloid-β Peptide

Aβ_25–35_ and scrambled Aβ_25–35_ peptides (Eurogentec, France) were dissolved in sterile water (1 μg/μL) and stored at −20 °C. Since soluble Aβ oligomers correlate better with the progression of the disease,^29^ Aβ_25–35_ and scrambled peptides were preaggregated by an *in vitro* incubation at 37 °C (4 days) to obtain a solution mainly composed (more than 95%) of a mixture of soluble oligomer species (oAβ_25–35_), as previously characterized.^64^

#### Experimental Procedures for oAβ_25–35_ Model of AD

As previously detailed,^37,64^ to induce the acute model of AD (oAβ_25–35_ model), rats were divided into three groups. One group had no surgery (Naive group), a second set of animals received an intracerebroventricular (*icv*) injection of incubated scrambled peptide (negative control groups—10 μg/rat—Sc), and a third set received an *icv* injection of oAβ_25–35_ (AD groups—10 μg/rat—Aβ). The animals were anesthetized with an intraperitoneal (*ip*) injection of a mixture of ketamine and xylazine (80 and 10 mg/kg b.w., respectively). Scrambled peptide and oAβ_25–35_ were injected directly into the lateral ventricles at a rate of 2 μL/min using a David-Kopf stereotaxic apparatus (coordinates of injection: AP −1 mm, L ±1.5 mm, DV −3.5 mm).

In order to determine the therapeutic potential of the two 5-HT_6_R antagonists (intepirdine (INTEP) and **PZ-1922**) in AD pathology induced by the *icv* injection of oAβ_25–35_, two therapeutic approaches were evaluated.

1—A curative approach: This treatment was started 1 week after the induction of amyloid toxicity (*icv injection of oAβ*_*25–35*_) and consisted of 2 *ip* injections (1.5 mg/kg per injection) per day for 1 week. On day 14, animals were subjected to a behavioral memory test (T-maze) and sacrificed on day 15 by decapitation.

2—A preventive approach: This treatment was started at the same time as the induction of amyloid toxicity and consisted of implanting *ip*, and during the same anesthetic procedure necessary for the *icv* injection of oAβ_25–35_, an osmotic pump (Alzet-EML2, Charles-River, France) which continuously delivered the molecules for 1 week at the dose of 3 mg/kg/day. On day 7, animals were submitted to a behavioral memory test (T-maze) and sacrificed on day 8 by decapitation.

At the time of sacrifice, all hippocampi were dissected out, weighed, and rapidly frozen in liquid nitrogen for Western blot (WB) analysis. Vehicle rats (*N–V*, *Sc–V*, *A*β*-V groups*) received only *ip* injections (or Alzet pump) of vehicle (sterile 0.9% NaCl) and served as negative controls for pharmacological treatments (*N-INTEP*, *N-PZ1922*, *Sc-INTEP*, *Sc-PZ1922*, *A*β*-INTEP*, and *A*β*-PZ1922 groups*).

#### Spatial Short-Term Memory Deficits in the T-Maze Test in Rats

As previously reported, the T-maze test was used to rapidly assess cognitive ability in rats, especially the short-term memory deficits, when performed in two successive sessions.^37,38,64,65^ The T-maze consisted of two short arms (A and B), extending from a longer alley (C) and enclosed with high walls. The test involved two trials, separated by 1 h. During the training session, one short arm (B) was closed. Rats were placed at the end of the long alley, allowed to visit the maze for 10 min, and then returned to their home cage. During the test session, which was videotracked (Noldus EthoVisonXT, France), animals were placed in the maze for 2 min, with free access to all arms. The number of visits and time spent in each arm were measured. The results were expressed as the ratio of the time spent in the initially closed novel arm, over the time spent in the previous arm, and as a ratio of the number of entries into the novel arm over the familiar one. The apparatus was cleaned with diluted ethanol (50%) between animals.

#### Western Blot Analysis

Western blot analyses were performed on the whole hippocampus as previously described.^37^ All antibodies used are detailed in Table S3. Briefly, after sacrifice, hippocampi were microdissected, weighed, immediately frozen in liquid nitrogen, and stored at −20 °C. Tissues were sonicated (VibraCell; Sonics & Materials, USA) in a lysis buffer^37^ and centrifuged (4 °C). Supernatants were collected, and the protein concentration was measured using a bicinchoninic Acid (BCA) assay (ThermoFisher Scientific, France). Samples (60 μg protein) were separated on SDS-polyacrylamide gels (12%) and transferred to PVDF membranes (Merck-Millipore, France). The membranes were incubated overnight (4 °C) with the primary antibody, washed, and then incubated for 2 h with the appropriate horseradish peroxidase-conjugated secondary antibody. Peroxidase activity was revealed by using enhanced chemiluminescence (ECL) reagents (Luminata-Crescendo, Merck-Millipore). The intensity of the immunoreactive signals was quantified using Image-J software (NIH, Bethesda, MA, USA). β-Tubulin (β-Tub) was used as a loading control for all experiments.

#### Statistical Analysis

Data are presented as box and whiskers (min to max), including the median value. Before each analysis of variance, the Gaussian distribution was evaluated and validated by a Kolmogorov–Smirnov test (GraphPad-Prism 9.0). Nonparametric tests were used (Mann–Whitney & Dunn’s) when the distribution was not normal. Two- or one-way ANOVA followed by Tukey’s or Dunnett’s multiple comparison tests were used for the normally distributed data set and were resumed in Tables S4 and S6. *P* < 0.05 was considered significant. The number of animals in each group is indicated in the figure legends. Statistical power analysis was calculated using G*Power.

## Supporting Information Available

Synthesis of compounds **22** and **24–26**; characterization data for final compounds **3**, **4**, **6**, **7–9**, **11–15**, **17–19**, **21**, **22**, and **24–26**; UPLC, ^1^H NMR, ^13^C NMR spectra of final compounds **5**, **10**, **15–18**, **20**, and **23**; assessment of physicochemical parameters of **PZ-1922**, selectivity screen for **PZ-1922**; *in silico* evaluation of **PZ-1922** in 5-HT R and MAO-B, cryo-EM studies on **PZ-1922** at 5-HT_3_R; pharmacokinetic evaluation of **PZ-1922**, results of the biochemical analysis of the impact of **PZ-1922** and intepirdine on apoptotic processes in the curative and preventive treatments; and results of the biochemical analysis of curative and preventive treatment with **PZ-1922** and intepirdine (PDF)
5-HT_3_R cryo-EM structure with **PZ-1922** (PDB ID: 8CC6; EMDB ID: EMD-16555) 5-HT_3_R cryo-EM structure with **PZ-1939** (PDB ID: 8CC7; EMDB ID: EMD-16557) 5-HT_6_ receptor crystal structure (PDB ID: 7XTB PDB) MAO-B crystal structure (PDB ID: 2V5Z PDB) 5-HT_6_ receptor complex with **PZ-1922** 5-HT_6_ receptor complex with **PZ-1939** 5-HT_6_ receptor complex with FPPQ MAO–B complex with **PZ-1922** MAO–B complex with PZ-1771 SMILE strings of all shown molecules and corresponding biological activity (CSV)
PDB coordinates of 5-HT_6__**PZ-1922** (PDB)
PDB coordinates of 5-HT_6__**FPPQ** (PDB)
PDB coordinates of 5-HT_6__**PZ-1939** (PDB)
PDB coordinates of MAO_**PZ-1922** (PDB)
PDB coordinates of MAO_**PZ-1771** (PDB)

## Supplementary Material

jm3c01482_si_002.csv

jm3c01482_si_003.pdb

jm3c01482_si_004.pdb

jm3c01482_si_006.pdb

jm3c01482_si_007.pdb

jm3c01482_si_005.pdb

## Abbreviations

AChE: acetylcholinesterase
AcOEt: ethyl acetate
AD: Alzheimer’s disease
BBB: blood–brain barrier
BINAP: 2,2′-bis(diphenylphosphino)-1,1′-binaphthyl
BTPP: phosphazene base P1-*t*-Bu-tris(tetramethylene)
Casp 3: caspase type 3
CNS: central nervous system
cryo-EM: cryo-electron microscopy
5-CT: 5-carboxamidotryptamine
DAO: diamine oxidase
GABA: gamma aminobutyric acid
GFAP: glial fibrillary acidic protein
GPCRs: G protein-coupled receptors
*h*ERG: human Ether-à-go-go-Related Gene
5-HT: serotonin
Hex: hexane
Iba1: ionized calcium-binding adaptor molecule 1
*icv*: intracerebroventricular
*ig*: intragastric
INTEP: intepirdine
*ip*: intraperitoneal
*iv*: intravenous
MAO-B: monoamine oxidase type B
MeOH: methanol
MD: molecular dynamics
NOR: novel object recognition
oAβ: oligomeric solution of amyloid-β peptide
Pd[(C_6_H_5_)_3_P]_4_: tetrakis (triphenylphosphine) palladium(0)
Pd_2_(dba)_3_: tris(dibenzylideneacetone)dipalladium(0)
Pd(dppf)Cl_2_: [1,1′-bis(diphenylphosphino)ferrocene]dichloropalladium(II)
PSD-9: postsynaptic density protein 95
SAR: structure activity relationship
SCOP: scopolamine
Scr: scrambled peptide
SEM: standard error of the mean
SERT: serotonin transporter
SYN: synaptotagmin
*t*-BuOH: *tert*-butanol
TEVC: two-electrode voltage-clamp
