# Supplementary material for: Superiority of the Triple-Acting 5-HT6R/5-HT3R Antagonist and MAO-B Reversible Inhibitor PZ-1922 over 5-HT6R Antagonist Intepirdine in Alleviation of Cognitive Deficits in Rats
Source: J Med Chem. 2023 Oct 5;66(21):14928–47. doi: 10.1021/acs.jmedchem.3c01482 (PMC10641814; doi:10.1021/acs.jmedchem.3c01482)
Supplement: Supplementary file 1 — jm3c01482_si_001.pdf [file jm3c01482_si_001.pdf]

SUPPORTING INFORMATION

Superiority of the triple-acting 5-HT$_6$R/5-HT$_3$R antagonist and MAO-B reversible inhibitor PZ-1922 over 5-HT$_6$R antagonist intepirdine in alleviation of cognitive deficits in rats

Katarzyna Grychowska,[1] Uriel López Sánchez,[2] Mathieu Vitalis,[3] Geoffrey Canet,[4] Grzegorz Satała,[5] Agnieszka Olejarz-Maciej,[1] Joanna Gołębiowska,[5] Rafał Kurczab,[5] Wojciech Pietruś,[5] Monika Kubacka,[1] Christophe Moreau,[2] Maria Walczak,[1] Klaudia Blicharz-Futera,[1] Xavier Bantreil,[6] Gilles Subra,[6] Andrzej J. Bojarski,[5] Frédéric Lamaty,[6] Carine Becamel,[7] Charleine Zussy,[3] Séverine Chaumont-Dubel,[7] Piotr Popik,[5] Hugues Nury,[2] Philippe Marin,[7] Laurent Givalois[3,4,8] and Paweł Zajdel[1,*]

[1] Faculty of Pharmacy Jagiellonian University Medical College, 9 Medyczna Str., 30-688 Kraków, Poland

[2] Univ. Grenoble Alpes, CNRS, CEA, IBS, F-38000 Grenoble, France

[3] Molecular Mechanisms in Neurodegenerative Dementia (MMDN) Laboratory, University of Montpellier, EPHE-PSL, INSERM U1198, 34-095 Montpellier, France

[4] Faculty of Medicine, Laval University, CR-CHUQ, G1V 4G2, Québec city (QC), Canada

[5] Maj Institute of Pharmacology, Polish Academy of Sciences, 12 Smętna Str., 31-324 Kraków, Poland

[6] IBMM, Université de Montpellier, CNRS, ENSCM, 34-293 Montpellier, France

[7] Institut de Génomique Fonctionnelle, Université de Montpellier, CNRS, INSERM, 34-094 Montpellier, France

[8] CNRS, 75-016 Paris, France

*Corresponding author, e-mail: pawel.zajdel@uj.edu.pl

Table of content

1. Chemistry ... 3
1.1.Synthesis of compounds **25** and **26**. ... 3
1.2.Characterization data for final compounds **3**, **4**, **6**, **7–9**, **11** and **12**. ... 4
1.3.Characterization data for final compounds, **13–15**, **17–19** and **21**. ... 7
1.4.Procedures for preparation and characterization data for final compounds **22**, **24–26** *and intermediates* **II** *and* **IV**. ... 9
1.5. UPLC spectra, ^{1}H NMR and ^{13}C NMR spectra of representative compounds **5**, **10**, **15–18**, **20** and **23**. ... 12
2. Assessment of physicochemical parameters of PZ-1922 ... 24
3. Selectivity screen of PZ-1922 ... 25
4. *In silico* evaluation of PZ-1922 in 5-HT$_6$R and MAO-B ... 27
Figure S-1. The QM and MD data analysis results were obtained for **PZ-1922** and **PZ-1939** in the 5-HT$_6$R. ... 27
Figure S-2. The QM and MD data analysis results were obtained for **PZ-1922** and **PZ-1771** in the MAO-B enzyme. ... 28
5. Cryo-EM studies on PZ-1922 at 5-HT$_3$R ... 29
Figure S-3. Image analysis workflow and quality density maps of the m5-HT$_3$AR in complex with **PZ-1922**. ... 29
Figure S-4. Image analysis workflow and quality density maps of the m5-HT$_3$AR in complex with **PZ-1939**. ... 30
Table S-1. Cryo-EM data collection, refinement and validation statistics. ... 31
Figure S-5. Comparison of ligand densities for **PZ-1922** and **PZ-1939**. ... 32
Figure S-6. Comparison of ligand poses for **PZ-1922** with palonosetron and **PZ-1939** ... 32
6. Pharmacokinetic evaluation of PZ-1922 ... 33
Table S-2. Pharmacokinetic parameters and brain uptake for **PZ-1922** after intravenous and intragastric administration 33
7. Results of the biochemical analysis of the impact of PZ-1922 and intepirdine on apoptotic processes in the curative and preventive treatment ... 35
Figure S-7. The impact in the hippocampus of the curative (A-C) and preventive (D-F) strategies with intepirdine (INTEP) and **PZ-1922** on apoptotic processes induced by the *icv* injection of A$\beta_{25\text{-}35}$ ... 35
8. Results of the biochemical analysis of curative treatment with PZ-1922 and intepirdine ... 36
Figure S-8. Impact of curative strategy with intepirdine (INTEP) and **PZ-1922** on A$\beta_{25\text{-}35}$-induced toxicity ... 36
Figure S-9. The impact in the hippocampus of the curative strategy with intepirdine (INTEP) and **PZ-1922** on Cdk5 activity (A-H) induced by the *icv* injection of A$\beta_{25\text{-}35}$ ... 37
Figure S-10. The impact in the hippocampus of the curative strategy with intepirdine (INTEP) and **PZ-1922** on neuroinflammation (A-D) and apoptotic processes (E-H) induced by the *icv* injection of A$\beta_{25\text{-}35}$ ... 38
9. Results of the biochemical analysis of preventive treatment with PZ-1922 and intepirdine ... 39
Figure S-11. Impact of preventive strategy with intepirdine (INTEP) and **PZ-1922** on A$\beta_{25\text{-}35}$-induced toxicity. ... 39
Figure S-12. The impact in the hippocampus of the preventive strategy with intepirdine (INTEP) and **PZ-1922** on Cdk5 activity (A-H) induced by the *icv* injection of A$\beta_{25\text{-}35}$ ... 40
Figure S-13. The impact in the hippocampus of the preventive strategy with intepirdine (INTEP) and **PZ-1922** on neuroinflammation (A-D) and apoptotic processes (E-H) induced by the *icv* injection of A$\beta_{25\text{-}35}$ ... 41
Table S-3. Antibodies used in Western blot experiments ... 42
Table S-4. Statistical analysis of principal figures ... 43
Table S-5. Statistical analysis of Figure S-7. ... 43
Table S-6. Statistical analysis of supplementary figures. ... 44
References ... 47

1. Chemistry

1.1.Synthesis of compounds **25** and **26**.

Synthesis of quinoline derivative **25** comprised two subsequent microwave-assisted substitutions of 2,4-dichloroquinoline **I**. First one consisted in the introduction of 3-chlorobenzyl amine upon heating in DMSO whereas second one involved coupling of the 3-chlorobenzyl derivative **II** with Boc-piperazine in a presence of TEA in acetonitrile (Scheme S-1A). On the other hand, pyridine derivative **26** was obtained in two steps: reductive amination of 2-bromopyridin-4-amine **III** upon treatment with 3-chlorobenzaldehyde and sodium cyanoborohydride in ethanol, followed by substitution of the 3-chlorobenzyl derivative **IV** with Boc-piperazine in a presence of triethylamine (TEA) in toluene (Scheme S-1B).

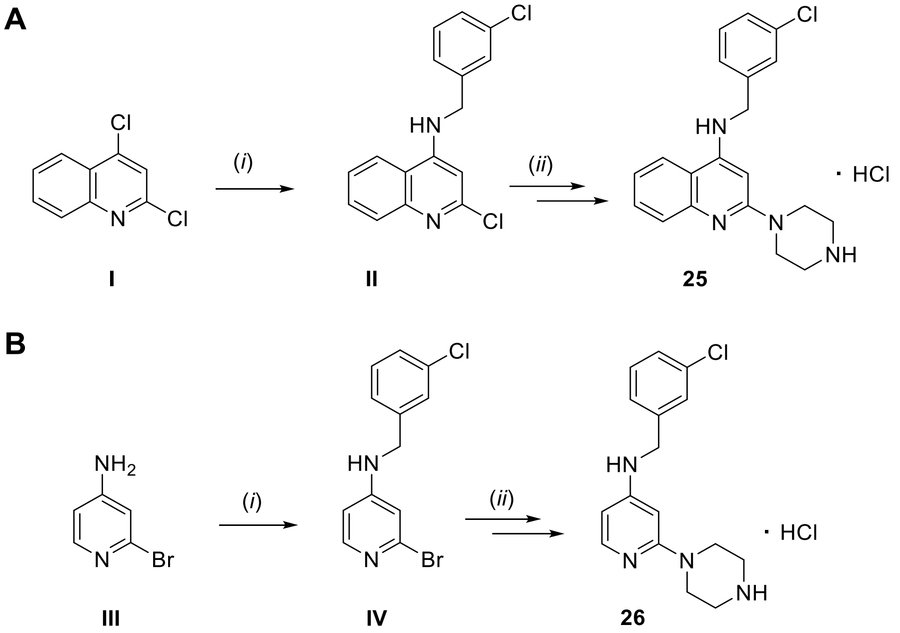

Scheme S-1. (A) Synthetic pathway leading to quinoline derivative **25** (*i*) 3-chlorobenzyl amine, DMSO, 140 °C, MW, 2h. (*ii*) Boc-piperazine, acetonitrile, 140 °C, 12h (B) Synthetic pathway leading to pyridine derivative **26** (*i*) 3-chlorobenaldehyde, $NaBH_3CN$, ethanol, rt, 12h (*ii*) Boc-piperazine, TEA, toluene, 114 °C, 12h..

1.2. Characterization data for final compounds **3**, **4**, **6**, **7**–**9**, **11** and **12**.

(S)-1-Benzyl-N-(pyrrolidin-3-yl)-1H-pyrrolo[3,2-c]quinolin-4-amine hydrochloride **3**
White solid, overall yield 53%, t_R = 4.33min, Mp 246–248 °C, $C_{22}H_{23}ClN_4$, MW 378.90. ^{1}H NMR (500 MHz, methanol-d_4) δ ppm 2.14–2.34 (m, 1H), 2.50–2.69 (m, 1H), 3.42–3.50 (m, 1H), 3.61–3.75 (m, 2H), 3.91 (dd, J = 12.6, 6.8 Hz, 1H), 4.95–5.13 (m, 1H), 5.86 (s, 2H), 6.95–7.06 (m, 2H), 7.21–7.26 (m, 3H), 7.43 (ddd, J = 8.3, 7.2, 1.2 Hz, 1H), 7.56–7.70 (m, 3H), 7.92–8.06 (m, 1H), 8.08–8.13 (m, 1H). ^{13}C NMR (126 MHz, methanol-d_4) δ ppm 33.80, 45.81, 50.42, 53.98, 54.80, 101.84, 109.01, 116.30, 119.10, 121.44, 125.62, 126.46, 127.69, 127.98, 129.16, 129.37, 130.81, 134.46, 136.22, 137.11, 140.97, 149.84. Monoisotopic mass 342.44, $[M+H]^+$ = 343.4. HRMS calcd for $C_{22}H_{23}N_4$ 342.1844 found 342.1832.

(S)-1-(3-Fluorobenzyl)-N-(pyrrolidin-3-yl)-1H-pyrrolo[3,2-c]quinolin-4-amine hydrochloride **4**
White solid, overall yield 46%, t_R = 4.77 min, Mp 121–123 °C, $C_{22}H_{22}ClFN_4$, MW 396.89. ^{1}H NMR (500 MHz, dmso-d_6) δ ppm 2.23 (d, J = 4.69 Hz, 1H), 2.42–2.52 (m, 2H), 3.33 (bs, 2H), 3.61–3.71 (m, 2H), 5.41 (bs, 1H), 5.95 (s, 2H), 6.75–6.92 (m, 2H), 7.05 (dt, J = 1.8, 8.50 Hz, 1H), 7.28–7.39 (m, 1H), 7.53 (t, J = 7.6 Hz, 1H), 7.70 (d, J = 3.5 Hz, 1H), 7.79 (d, J = 2.9 Hz, 1H), 7.94 (d, J = 8.2 Hz, 1H), 8.50 (d, J = 8.2 Hz, 1H), 9.60–9.79 (m, 1H), 9.85 (d, J = 7.6 Hz, 1H). ^{13}C NMR (126 MHz, dmso-d_6) δ ppm 31.15, 32.75, 43.51, 46.49, 52.03, 55.21, 103.97, 113.28, 115.87, 119.08, 123.87, 124.01, 124.87, 126.79, 127.51, 129.18, 130.97, 131.72, 133.44, 134.67, 138.99, 148.02. Monoisotopic mass 360.18, $[M+H]^+$ = 361.2. HRMS calcd for $C_{22}H_{22}FN_4$ 360.1750 found 360.1742.

(R)-1-(3-Chlorobenzyl)-N-(pyrrolidin-3-yl)-1H-pyrrolo[3,2-c]quinolin-4-amine hydrochloride **6**

White solid, overall yield 49%, t_R = 4.78 min, Mp 199–201 °C, $C_{22}H_{22}Cl_2N_4$, MW 413.35. ^{1}H NMR (500 MHz, methanol-*d4*) δ ppm 2.14–2.28 (m, 1H), 2.45–2.61 (m, 1H), 3.63–3.71 (m, 1H), 3.76–3.82 (m, 2H), 3.89–3.96 (m, 1H), 5.16–5.24 (m, 1H), 6.04 (s, 2H), 6.97–7.12 (m, 2H), 7.28–7.37 (m, 2H), 7.41–7.52 (m, 1H), 7.58–7.64 (m, 3H), 8.01 (d, *J* = 8.2 Hz, 1H), 8.05 (d, *J* = 8.2 Hz, 1H). ^{13}C NMR (126 MHz, methanol-*d4*) δ ppm 32.16, 45.83, 51.63, 52.99, 53.68, 105.83, 111.88, 115.42, 120.21, 123.11, 125.62, 126.81, 127.07, 129.22, 130.10, 131.82, 133.30, 135.45, 135.57, 136.20, 140.22, 149.98. Monoisotopic mass 376.15, $[M+H]^+$ = 377.2. HRMS calcd for $C_{22}H_{22}ClN_4$ 377.1633 found 377.1489.

(S)-1-(3-Bromobenzyl)-N-(pyrrolidin-3-yl)-1H-pyrrolo[3,2-c]quinolin-4-amine hydrochloride **7**

White solid, overall yield 52%, t_R = 4.78 min, Mp 274–276 °C, $C_{22}H_{22}BrClN_4$, MW 457.80^{1}H NMR (500 MHz, methanol-*d4*) δ ppm 2.18–2.25 (m, 1H), 3.31–3.50 (m, 2H), 3.63–3.71 (m, 2H), 3.88–3.97 (m, 1H), 4.89–5.13 (m, 1H), 5.98 (s, 2H), 6.73–6.82 (m, 2H), 7.18–7.21 (m, 2H), 7.47 (ddd, *J* = 8.3, 7.2, 1.2 Hz, 1H), 7.51–7.59 (m, 3H), 7.73–7.90 (m, 2H). ^{13}C NMR (126 MHz, methanol-*d4*) δ ppm 32.12, 46.15, 50.75, 53.03, 53.88, 105.82, 111.80, 115.33, 119.98, 123.12, 125.55, 126.60, 126.98, 129.12, 130.18, 131.85, 131.99, 135.23, 136.39, 136.57, 141.02, 149.12. Monoisotopic mass 420.09, $[M+H]^+$ = 421.1. HRMS calcd for $C_{22}H_{22}BrN_4$ 421.1028 found 421.1093.

(S)-1-(3-Methoxybenzyl)-N-(pyrrolidin-3-yl)-1H-pyrrolo[3,2-c]quinolin-4-amine hydrochloride **8**

White solid, overall yield 47%, t_R = 4.64 min, Mp 136–137 °C, $C_{23}H_{25}ClN_4O$, MW 408.93^{1}H NMR (500 MHz, dmso-*d6*) δ ppm 2.20–2.28 (m, 1H), 3.49–3.56 (m, 1H), 3.57–3.64 (m, 2H), 3.67 (s, 3H), 5.35 (s, 1H), 5.75 (s, 1H), 5.91 (s, 2H), 6.53 (d, *J* = 7.7 Hz, 1H), 6.62 – 6.65 (m, 1H), 6.82 (dd, *J* = 8.3, 2.6 Hz, 1H), 7.22 (t, *J* = 8.0 Hz, 1H), 7.34–7.40 (m, 1H), 7.54–7.59 (m, 1H), 7.68–7.79 (m, 2H), 8.01 (d, *J* = 8.3 Hz, 1H), 8.41–8.49 (m, 1H). ^{13}C NMR (126 MHz, dmso-*d6*) δ ppm 32.60, 36.70, 43.94, 47.46, 51.26, 55.16, 104.72, 112.44, 115.39, 119.00, 122.41, 123.50, 124.78, 126.80, 127.73, 129.20, 130.97, 131.83, 133.50, 134.57, 139.72, 148.18. Monoisotopic mass 372.20, $[M+H]^+$ = 373.2. HRMS calcd for $C_{23}H_{25}N_4O$ 372.1950 found 372.1961.

(R)-1-(3-Chlorobenzyl)-N-(pyrrolidin-2-ylmethyl)-1H-pyrrolo[3,2-c]quinolin-4-amine hydrochloride **9**

White solid, overall yield 41%, t_R = 3.27 min, Mp 123–125 °C, $C_{23}H_{24}Cl_2N_4$, MW 427.37. ^{1}H NMR (500 MHz, methanol-*d₄*) δ ppm 1.92–1.99 (m, 1H), 2.07–2.15 (m, 1H), 2.17–2.25 (m, 1H), 2.37 –2.46 (m, 1H), 3.35–3.43 (m, 1H), 3.45–3.53 (m, 1H), 4.10–4.20 (m, 2H), 4.21–4.28 (m, 1H), 5.83 (s, 2H), 6.96–7.00 (m, 2H), 7.19–7.30 (m, 2H), 7.35 (t, *J* = 7.7 Hz, 1H), 7.44 (d, *J* = 3.1 Hz, 1H), 7.52–7.57 (m, 2H), 7.90 (d, 1H), 8.20 (d, *J* = 8.3 Hz, 1H). ^{13}C NMR (126 MHz, methanol-d_4) δ ppm 24.25, 29.05, 44.29, 46.78, 53.63, 60.29, 105.33, 111.82, 115.38, 120.17, 123.08, 125.50, 126.73, 126.98, 129.17, 130.05, 131.77, 133.34, 135.40, 136.17, 140.19, 150.53. Monoisotopic mass 390.16, $[M+H]^+$ = 391.1. HRMS calcd for $C_{23}H_{24}ClN_4$ 391.1689 found 391.1694.

(S)-1-(3-Chlorobenzyl)-N-(pyrrolidin-3-ylmethyl)-1H-pyrrolo[3,2-c]quinolin-4-amine hydrochloride **11**

White solid, overall yield 41%, t_R = 4.58 min, Mp 155–157 °C, $C_{23}H_{24}Cl_2N_4$, MW 427.37. ^{1}H NMR (500 MHz, dmso-d_6) δ ppm 1.74–1.83 (m, 1H), 2.10–2.17 (m, 1H), 2.76–2.86 (m, 1H), 3.03–3.16 (m, 2H), 3.83–3.92 (m, 1H), 3.97–4.07 (m, 1H), 5.93 (s, 2H), 6.92 (d, *J* = 6.6 Hz, 1H), 7.12 (s, 1H), 7.26–7.35 (m, 3H), 7.52 (t, *J* = 7.8 Hz, 1H), 7.55–7.69 (m, 2H), 7.93 (d, *J* = 8.1 Hz, 1H), 8.50 (s, 1H), 9.34 (d, *J* = 34.5 Hz, 2H), 9.91 (s, 1H), 12.88 (s, 1H). ^{13}C NMR (126 MHz, dmso-d_6) δ ppm 27.60, 36.96, 43.94, 44.20, 47.46, 51.71, 104.72, 110.47, 113.48, 119.09, 121.40, 124.50, 124.73, 125.78, 127.63, 128.18, 130.90, 131.70, 132.49, 133.56, 139.55, 148.77. Monoisotopic mass 390.16, $[M+H]^+$ = 391.1. HRMS calcd for $C_{23}H_{24}ClN_4$ 391.1689 found 391.1694.

(R)-1-(3-Chlorobenzyl)-N-(pyrrolidin-3-ylmethyl)-1H-pyrrolo[3,2-c]quinolin-4-amine hydrochloride **12**

White solid, overall yield 45%, t_R = 4.59 min, Mp 134–136 °C, $C_{23}H_{24}Cl_2N_4$, MW 427.37. ^{1}H NMR (500 MHz, dmso-d_6) δ ppm 1.74–1.83 (m, 1H), 2.08–2.17 (m, 1H), 2.75–2.85 (m, 1H), 3.02–3.14 (m, 2H), 3.83–3.91 (m, 1H), 3.98–4.05 (m, 1H), 5.92 (s, 2H), 6.91 (dt, *J* = 7.0, 1.8 Hz, 1H), 7.11 (s, 1H), 7.25–7.34 (m, 3H), 7.50 (t, *J* = 7.8 Hz, 1H), 7.56 (s, 1H), 7.65 (d, *J* = 3.2 Hz, 1H), 7.92 (d, *J* = 8.2 Hz, 1H), 8.49 (s, 1H), 9.34 (d, *J* = 28.4 Hz, 2H), 9.91 (s, 1H), 12.88 (s, 1H). ^{13}C NMR (126 MHz, dmso-d_6) δ ppm 27.62, 37.00, 43.95, 44.20, 47.48, 51.73, 104.77, 110.49, 113.51, 119.12, 121.42, 124.52, 124.72, 125.80, 127.65, 128.18, 130.92, 131.70, 132.52, 133.59, 139.58, 148.79. Monoisotopic mass 390.16, $[M+H]^+$ = 391.1. HRMS calcd for $C_{23}H_{24}ClN_4$ 391.1689 found 391.1694.

1.3.Characterization data for final compounds, **13**–**15**, **17**–**19** and **21**.

1-(1-(3-Chlorobenzyl)-1H-pyrrolo[3,2-c]quinolin-4-yl)-N,N-dimethylpyrrolidin-3-amine hydrochloride **13**
White solid, overall yield 38%, t_R = 4.61 min, Mp 165–166 °C, $C_{24}H_{26}Cl_2N_4$, MW 441.40. ^{1}H NMR (500 MHz, methanol-d_4) δ ppm 2.40 (s, 6H), 2.95–3.03 (m, 1H), 3.59–3.66 (m, 1H), 3.73 (d, *J* = 14.5, 11.7, 6.8 Hz, 2H), 3.94 (t, *J* = 10.4, 6.7 Hz, 1H), 4.11–4.20 (m, 2H), 5.78 (s, 2H), 6.90–6.97 (m, 2H), 7.07 (d, *J* = 3.3 Hz, 1H), 7.13 (ddd, *J* = 8.4, 7.1, 1.3 Hz, 1H), 7.21–7.26 (m, 2H), 7.36–7.42 (m, 2H), 7.81 (ddd, *J* = 11.5, 8.4, 1.3 Hz, 2H). ^{13}C NMR (126 MHz, methanol-d_4) δ ppm 22.03, 31.18, 44.44, 50.51, 52.28, 65.54, 106.75, 112.61, 115.38, 122.32, 124.13, 125.45, 126.98, 128.84, 128.89, 131.07, 131.62, 136.03, 140.83. Monoisotopic mass 404.18, $[M+H]^+$ = 405.2. HRMS calcd for $C_{24}H_{26}ClN_4$ 405.1846 found 405.1816.

(S)-(1-(1-(3-Chlorobenzyl)-1H-pyrrolo[3,2-c]quinolin-4-yl)pyrrolidin-3-yl)methanamine hydrochloride **14**
White solid, overall yield 53%, t_R = 4.49 min, Mp 180–182 °C, $C_{23}H_{24}Cl_2N_4$, MW 427.37. ^{1}H NMR (500 MHz, dmso-d_6) δ ppm 1.11–1.25 (m, 1H), 1.45–1.75 (m, 2H), 2.00–2.25 (m, 2H), 3.33 (s, 1H), 3.90–4.07 (m, 2H), 4.41–4.65 (m, 1H), 5.63 (s, 2H), 6.68–6.72 (m, 2H), 6.75–6.80 (m, 1H), 7.13-7.18 (m, 2H), 7.27–7.35 (m, 2H), 7.65 (dd, *J* = 8.2, 1.0 Hz, 1H), 7.79 (d, *J* = 8.2 Hz, 1H), 7.80–8.02 (m, 1H). ^{13}C NMR (126 MHz, dmso-d_6) δ ppm 33.37, 52.75, 67.02, 112.0, 112.72, 118.18, 120.35, 128.20, 130.49, 159.96. Monoisotopic mass 390.16, $[M+H]^+$ = 391.1. HRMS calcd for $C_{23}H_{24}ClN_4$ 391.1689 found 391.1676.

1-(1-(3-Chlorobenzyl)-1H-pyrrolo[3,2-c]quinolin-4-yl)-N-methylazetidin-3-amine hydrochloride **15**
White solid, overall yield 45%, t_R = 3.73 min, Mp 119–121 °C, $C_{22}H_{22}Cl_2N_4$, MW 413.35. ^{1}H NMR (500 MHz, dmso-d_6) δ ppm 2.61 (s, 3H), 4.21–4.32 (m, 1H), 4.71–5.17 (m, 4H), 5.99 (s, 2H), 6.93 (dt, *J* = 7.3, 1.7 Hz, 1H), 7.05–7.10 (m, 2H), 7.29–7.36 (m, 3H), 7.56 (t, *J* = 7.8 Hz, 1H), 7.80 (d, *J* = 3.3 Hz, 1H), 7.99 (dd, *J* = 8.3, 1.2 Hz, 1H), 8.20 (d, *J* = 8.3 Hz, 1H). ^{13}C NMR (126 MHz, dmso-d_6) δ ppm 30.14, 47.89, 51.90, 57.24, 121.79, 124.53, 125.74, 127.76, 130.99, 133.34, 133.67, 139.39. Monoisotopic mass 376.15, $[M+H]^+$ = 377.2. HRMS calcd for $C_{22}H_{22}ClN_4$ 377.1533 found 377.1524.

1-(3-Chlorobenzyl)-4-(1,4-diazepan-1-yl)-1H-pyrrolo[3,2-c]quinoline hydrochloride **17**
White solid, overall yield 56%, t_R = 4.53 min, Mp 182–184 °C, $C_{23}H_{24}Cl_2N_4$, MW 427.37. ^{1}H NMR (500 MHz, methanol-d_4) δ ppm 2.48–2.54 (m, 2H), 3.49–3.54 (m, 2H), 3.75–3.81 (m,

2H), 4.30 (t, J = 5.7 Hz, 2H), 4.48–4.55 (m, 2H), 5.99 (s, 2H), 7.02–7.06 (m, 2H), 7.24 (d, J = 3.4 Hz, 1H), 7.26–7.34 (m, 2H), 7.41 (ddd, J = 8.4, 7.2, 1.2 Hz, 1H), 7.62 (ddd, J = 8.4, 7.2, 1.2 Hz, 1H), 7.69 (d, J = 3.4 Hz, 1H), 8.06 (dd, J = 8.4, 1.2 Hz, 1H), 8.17 (dd, J = 8.5, 1.1 Hz, 1H). ^{13}C NMR (126 MHz, methanol-d_4) δ ppm 25.84, 47.09, 47.23, 47.80, 50.84, 54.04, 108.27, 111.39, 114.91, 120.03, 123.08, 125.57, 126.76, 126.99, 129.21, 130.44, 131.81, 133.37, 135.38, 136.17, 137.20, 139.94, 152.00. Monoisotopic mass 390.16, $[M+H]^+$ = 391.2. HRMS calcd for $C_{23}H_{24}Cl_2N_4$ 391.1689 found 391.1676.

4-((1R,4R)-2,5-Diazabicyclo[2.2.1]heptan-2-yl)-1-(3-chlorobenzyl)-1H-pyrrolo[3,2-c]quinoline hydrochloride **18**

White solid, overall yield 48%, t_R = 5.40 min, Mp 178–180 °C, $C_{23}H_{22}Cl_2N_4$, MW 425.36. ^{1}H NMR (500 MHz, methanol-d_4) δ ppm 2.36 (d, J = 10.4 Hz, 1H), 2.54 (d, J = 11.2 Hz, 1H), 3.63–3.82 (m, 2H), 4.35–4.63 (m, 2H), 4.82 (s, 1H), 5.70 (bs, 1H), 5.97 (s, 2H), 7.00–7.06 (m, 2H), 7.25–7.34 (m, 3H), 7.39 (t, J = 7.6 Hz, 1H), 7.61 (t, J = 7.6 Hz, 1H), 7.68 (s, 1H), 8.04 (d, J = 8.2 Hz, 1H), 8.12 (d, J = 8.3 Hz, 1H). ^{13}C NMR (126 MHz, methanol- d_4) δ ppm 22.89, 52.68, 106.30, 113.51, 118.47, 121.83, 124.29, 125.29, 125.68, 127.89, 129.12, 130.51, 132.17, 134.21, 134.85, 135.46, 138.62. Monoisotopic mass 388.15, [M+H]+ = 389.2. HRMS calcd for HRMS calcd for $C_{23}H_{22}ClN_4$ 389.1533 found 389.1523.

1-(1-(3-Chlorobenzyl)-1H-pyrrolo[3,2-c]quinolin-4-yl)-N,N-dimethylpiperidin-4-amine hydrochloride **19**

White solid, overall yield 54%, t_R = 3.67 min, Mp 142–145 °C, $C_{25}H_{28}Cl_2N_4$, MW 455.43. ^{1}H NMR (500 MHz, methanol-d_4) δ ppm 2.06–2.15 (m, 2H), 2.44 (d, J = 12.3 Hz, 2H), 2.97 (s, 6H), 3.68 (t, J = 12.8 Hz, 2H), 3.73 J = 3.81 (m, 1H), 4.75 (d, J = 13.6 Hz, 2H), 5.97 (s, 2H), 7.02–7.06 (m, 2H), 7.25–7.33 (m, 3H), 7.42 (ddd, J = 8.4, 7.2, 1.2 Hz, 1H), 7.58–7.66 (m, 1H), 7.67–7.71 (m, 1H), 8.06 (td, J = 9.0, 8.5, 1.2 Hz, 2H). ^{13}C NMR (126 MHz, methanol-d_4) δ ppm 27.21, 30.75, 48.19, 53.47, 65.14, 74.54, 104.24, 115.28, 117.40, 121.77, 124.37, 125.43, 126.99, 127.69, 128.17, 128.74, 130.60, 131.05, 131.54, 131.89, 132.02, 132.81, 134.24, 141.47, 155.61. Monoisotopic mass 418.19, $[M+H]^+$ = 419.2. HRMS calcd for $C_{25}H_{28}ClN_4$ 419.2002 found 419.2025.

1-(3-Chlorobenzyl)-4-(2,6-diazaspiro[3.3]heptan-2-yl)-1H-pyrrolo[3,2-c]quinoline hydrochloride **21**

White solid, overall yield 45%, t_R = 3.57 min, Mp 145–147 °C, $C_{23}H_{22}Cl_2N_4$, MW 425.836 ^{1}H NMR (500 MHz, methanol-d_4) δ ppm 3.26–3.33 (m, 4H), 3.70–3.76 (m, 4H), 5.93 (s, 2H), 7.00 (dd, J = 17.2, 7.1 Hz, 2H), 7.10 (dd, J = 12.3, 3.0 Hz, 1H), 7.23–7.37 (m, 3H), 7.54–7.63 (m,

2H), 7.88–8.01 (m, 2H). ^{13}C NMR (126 MHz, methanol-*d_4*) δ ppm 40.31, 43.42, 56.00, 56.56, 106.12, 106.16, 110.54, 119.22, 123.19, 125.54, 126.95, 129.15, 130.21, 130.27, 131.78, 133.38, 135.56, 136.14, 140.11, 149.38. Monoisotopic mass 388.15, $[M+H]^+$ = 389.2. HRMS calcd for HRMS calcd for $C_{23}H_{22}ClN_4$ 389.1533 found 389.1523.

1.4. Procedures for preparation and characterization data for final compounds **22**, **24–26** *and intermediates* **II** *and* **IV**.

1-(3-Chlorobenzyl)-4-(1,2,3,6-tetrahydropyridin-4-yl)-1H-pyrrolo[3,2-c]quinoline hydrochloride **22**

Compound **2c** (0.17 g, 0.52 mmol, 1 eq), 3,6-dihydro-2*H*-pyridine-1-*N*-Boc-4-boronic acid pinacol ester (109 mg, 0.52 mmol, 1 eq), Pd(dppf)Cl_2 (15 mg, 0.021 mmol, 0.04 eq) and K_2CO_3 (287 mg, 2.08 mmol, 4 eq) were suspended in 4 mL dioxane/water (3/1, v/v). The vial was heated at 80 °C for 6 h under microwave-assisted conditions. The mixture was diluted with AcOEt, washed twice with saturated solution of NaCl, dried over Na_2SO_4 and evaporated. The crude product was purified using column chromatography and AcOEt/Hex (4/6, v/v) as a developing solvent. The obtained product was converted to HCl salt of secondary amine upon overnight treatment with 1M methanolic solution of HCl and subsequent filtration.

White solid, overall yield 52%, t_R = 4.20 min, Mp 123–125 °C, $C_{23}H_{21}Cl_2N_3$, MW 410.34. ^{1}H NMR (500 MHz, methanol-*d_4*) δ ppm 2.41 (d, *J* = 13.8 Hz, 2H), 2.71–2.83 (m, 1H), 3.42 (td, *J* = 12.6, 11.9, 2.9 Hz, 2H), 3.71 (d, *J* = 13.1 Hz, 2H), 6.15 (s, 2H), 7.02–7.09 (m, 1H), 7.12–7.15 (m, 1H), 7.29–7.35 (m, 2H), 7.70 (d, *J* = 3.4 Hz, 1H), 7.78 (t, *J* = 8.3, 7.1, 1.1 Hz, 1H), 7.93 (t, *J* = 8.4, 7.1, 1.2 Hz, 1H), 7.96 (d, *J* = 3.4 Hz, 1H), 8.40 (dd, *J* = 8.6, 1.2 Hz, 1H), 8.52 (d, *J* = 8.5 Hz, 1H). ^{13}C NMR (126 MHz, methanol-*d_4*) δ ppm 28.09, 44.97, 53.97, 106.99, 117.66, 121.05, 122.39, 123.64, 124.29, 125.64, 127.25, 128.45, 129.39, 129.83, 131.42, 131.92, 136.28, 136.52, 138.56, 139.49, 157.16. Monoisotopic mass 373.13, $[M+H]^+$ = 374.1. HRMS calcd for $C_{22}H_{22}ClN_4$ 374.1424 found 374.1416.

1-(3-Chlorobenzyl)-4-(piperazin-1-ylmethyl)-1H-pyrrolo[3,2-c]quinoline hydrochloride **24**

Compound **2c** (0.17 g, 0.52 mmol, 1 eq), potassium ((4-(*tert*-butoxycarbonyl)piperazin-1-yl)methyl)trifluoroborate (171 mg, 0.57 mmol, 1.1 eq), Pd[$(C_6H_5)_3P$]$_4$, (60 mg, 0.05 mmol, 0.1 eq) and K_2CO_3 (287 mg, 2.08 mmol, 4 eq) were dissolved in 4 mL of dioxane/water (3/1 v/v). The reaction vial was heated at 90˚C for 5h upon microwave-assisted conditions. The obtained product was converted to HCl salt of secondary amine upon overnight treatment with 1M methanolic solution of HCl and subsequent filtration.

White solid, overall yield 62%, t_R = 3.67 min, Mp 166–168 °C, $C_{23}H_{24}Cl_2N_4$, MW 427.37. ^{1}H NMR (500 MHz, methanol-d_4) δ ppm 2.95–3.02 (m, 4H), 3.34–3.40 (m, 4H), 4.52 (s, 2H), 6.10 (s, 2H), 7.03 (d, *J* = 7.5 Hz, 1H), 7.11–7.13 (m, 1H), 7.23–7.31 (m, 3H), 7.55 (d, *J* = 3.3 Hz, 1H), 7.74 (t, *J* = 7.8 Hz, 1H), 7.84–7.89 (m, 1H), 7.91 (d, *J* = 3.3 Hz, 1H), 8.36 (d, *J* = 8.4 Hz, 1H), 8.48 (d, *J* = 8.5 Hz, 1H). ^{13}C NMR (126 MHz, methanol-d_4) δ ppm 41.62, 44.61, 50.96, 53.87, 57.82, 106.97, 117.79, 121.92, 122.44, 123.66, 125.66, 127.22, 129.33, 129.82, 131.36, 131.88, 135.71, 136.16, 136.63, 138.21, 139.48, 152.41. Monoisotopic mass 390.16, $[M+H]^+$ = 391.2. HRMS calcd for $C_{23}H_{24}ClN_4$ 391.1689 found 391.1672.

N-(3-chlorobenzyl)-2-(piperazin-1-yl)quinolin-4-amine hydrochloride **25**

Compound **II** (100 mg, 0.3 mmol, 1 eq) was dissolved in MeCN (3 mL) followed by addition of Boc-piperazine (223 mg, 1.2 mmol, 4 eq). The reaction mixture was heated at 140°C for 12 h upon microwave assisted conditions. The solvent was evaporated and the crude product was purified using column chromatography and AcOEt/Hex (4/6, v/v) as a developing solvent. The obtained product was converted to HCl salt of secondary amine upon overnight treatment with 1M methanolic solution of HCl and subsequent filtration.

White solid, overall yield 37%, t_R = 3.67 min, $C_{20}H_{22}Cl_2N_4$, MW 389.32. ^{1}H NMR (500 MHz, DMSO-d_6) δ 3.31–3.38 (m, 4H), 3.46–3.55 (m, 4H), 5.00 (s, 2H), 6.72 (s, 1H), 7.32–7.51 (m, 4H), 7.60 (s, 1H), 7.73 (t, *J* = 7.7 Hz, 1H), 7.92 (d, *J* = 8.2 Hz, 1H), 8.30 (bs, 1H), 9.77 (s, 2H), 9.86–10.07 (m, 1H). Monoisotopic mass 352.15, $[M+H]^+$ = 353.2. HRMS calcd for $C_{20}H_{22}ClN_4$, 353.1533 found 353.1534.

N-(3-chlorobenzyl)-2-(piperazin-1-yl)pyridin-4-amine hydrochloride **26**

Compound **IV** (100 mg, 0.4 mmol, 1 eq) was dissolved in toluene (3 mL) followed by addition of Boc-piperazine (300 mg, 1.6 mmol, 4 eq). The reaction mixture was heated at 114°C for 12 h. The solvent was evaporated and the crude product was purified using column chromatography and AcOEt/Hex (5/5, v/v) as a developing solvent. The obtained product was converted to HCl salt of secondary amine upon overnight treatment with 1M methanolic solution of HCl and subsequent filtration.

White solid, overall yield 35%, t_R = 3.22 min, $C_{16}H_{20}Cl_2N_4$, MW 339.26. ^{1}H NMR (500 MHz, Methanol-d_4) δ 3.35–3.44 (m, 4H), 3.68–3.75 (m, 4H), 4.54 (s, 2H), 6.03 (s, 1H), 6.48 (dd, *J* = 7.2, 1.7 Hz, 1H), 7.28–7.41 (m, 4H), 7.60 (d, *J* = 7.0 Hz, 1H). Monoisotopic mass 302.13, $[M+H]^+$ = 303.2. HRMS calcd for $C_{16}H_{20}ClN_4$ 303.1376 found 303.1364.

2-Chloro-N-(3-chlorobenzyl)quinolin-4-amine **II**

2,4-Dichloroquinoline (400 mg, 2 mmol, 1 eq) was dissolved in DMSO. 3-Chlorobenzyl amine (338 mg, 2.4 mmol, 1.2 eq) was added and the mixture was heated at 140 °C for 2 h upon microwave-assisted conditions. The mixture was diluted with AcOEt, washed three times with water, once with saturated solution of NaCl and dried over Na_2SO_4. The solvent was evaporated and the crude product was purified using column chromatography and AcOEt/Hex (1/9, v/v) as a developing solvent.

White oil, yield 45%, t_R = 6.38 min, $C_{16}H_{12}Cl_2N_2$, MW 303.19.^{1}H NMR (500 MHz, Chloroform-*d*) δ 4.69 (d, *J* = 5.3 Hz, 2H), 5.62 (bs, 1H), 6.77 (s, 1H), 7.25 – 7.29 (m, 3H), 7.31 – 7.35 (m, 1H), 7.37 – 7.39 (m, 1H), 7.60 (ddd, *J* = 8.4, 6.9, 1.5 Hz, 1H), 7.70 – 7.73 (m, 1H), 8.00 (dd, *J* = 8.3, 1.5 Hz, 1H). Monoisotopic mass 302.04, $[M+H]^+$ = 302.9.

2-Bromo-N-(3-chlorobenzyl)pyridin-4-amine **IV**

2-Bromopyridin-4-amine (300 mg, 1.7 mmol, 1 eq)) was dissolved in ethanol (4 mL) and 3-chlorobenaldehyde (280 mg, 2 mmol, 1.2 eq) was added. The mixture was stirred overnight and $NaBH_3CN$ (213 mg, 3.4 mmol, 2 eq) was added as a solid. After 30 min of stirring the solvent was evaporated and the remaining crude product was purified using column chromatography and AcOEt/Hex 4/6 (v/v) as a developing solvent.

White oil, yield 72%, t_R = 5.40 min, $C_{12}H_{10}BrClN_2$, MW 297.58. ^{1}H NMR (500 MHz, Chloroform-*d*) δ 4.35 (d, *J* = 5.6 Hz, 2H), 5.05 (bs, 1H), 6.42 (dd, *J* = 5.8, 2.2 Hz, 1H), 6.66 (d, *J* = 2.2 Hz, 1H), 7.14–7.23 (m, 1H), 7.24–7.34 (m, 3H), 7.91 (d, *J* = 5.8 Hz, 1H). Monoisotopic mass 295.97, $[M+H]^+$ = 297.0.

1.5. UPLC spectra, ^{1}H NMR and ^{13}C NMR spectra of representative compounds **5**, **10**, **15–18**, **20** and **23**.

(S)-1-(3-Chlorobenzyl)-N-(pyrrolidin-3-yl)-1H-pyrrolo[3,2-c]quinolin-4-amine hydrochloride **5**

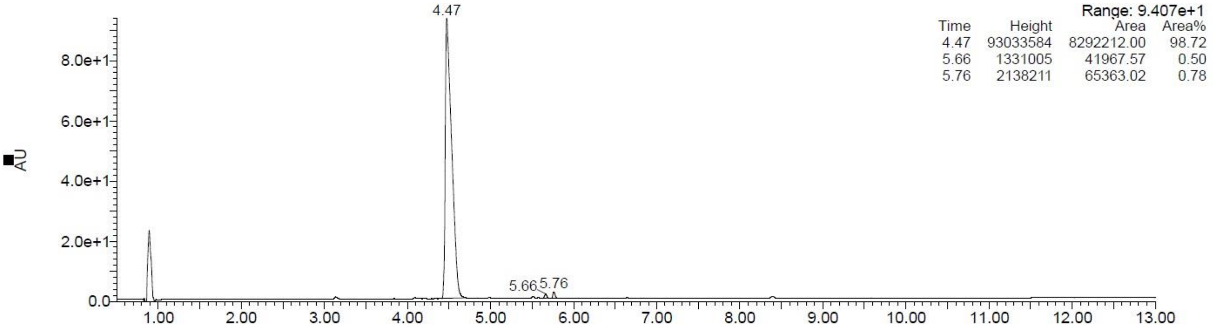

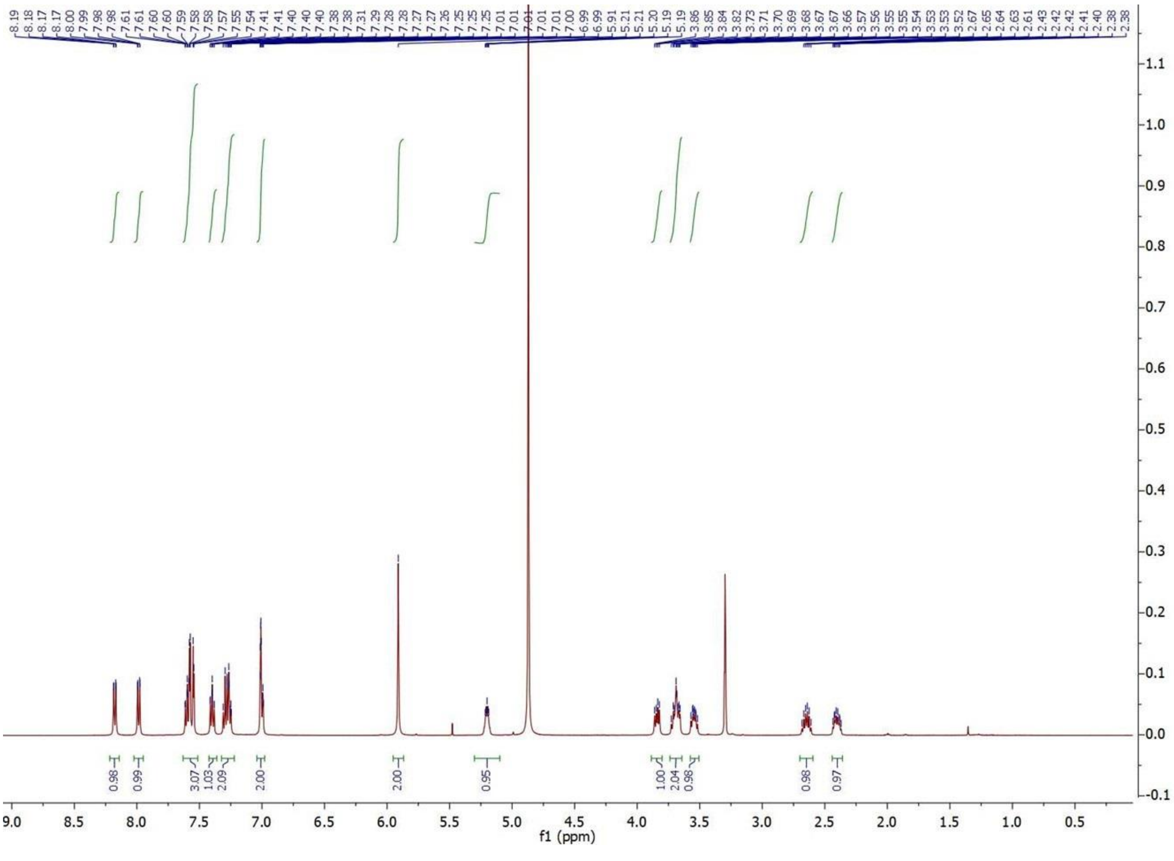

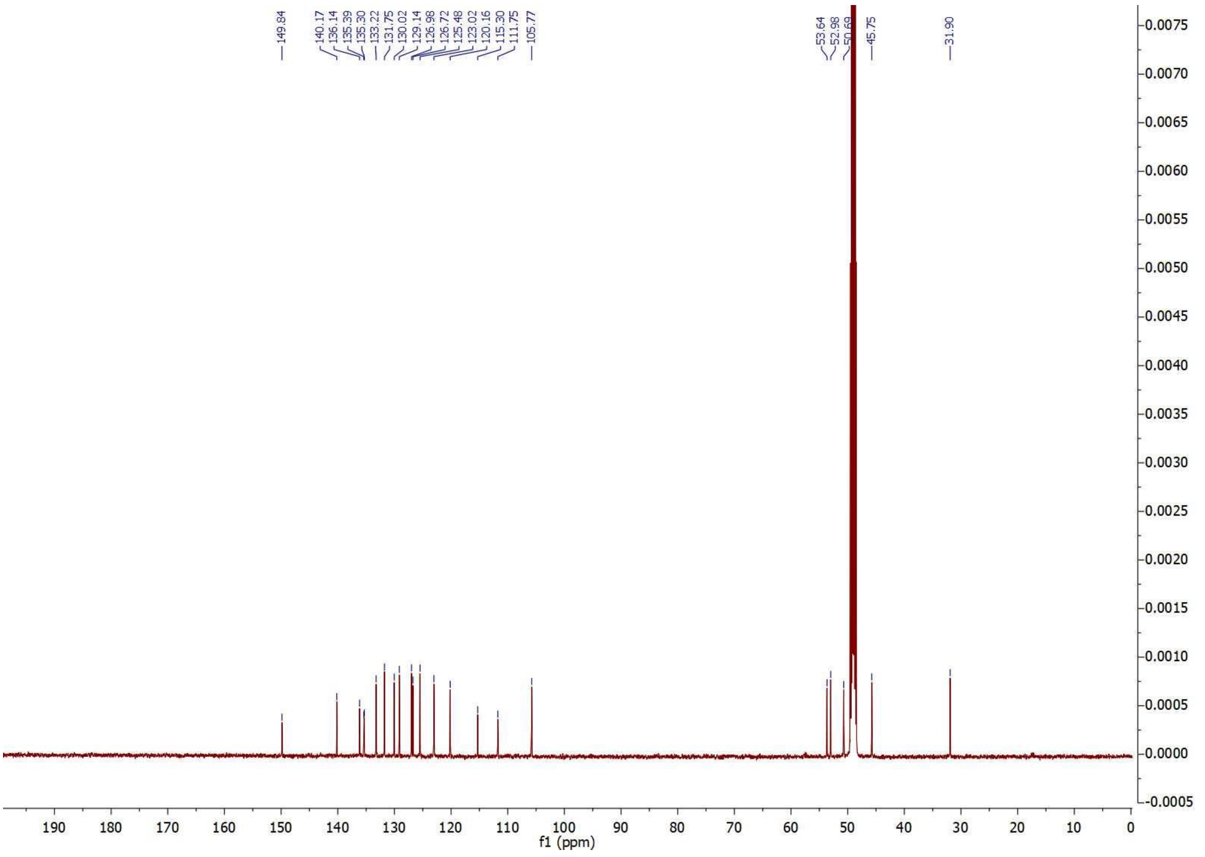

(S)-1-(3-Chlorobenzyl)-N-(pyrrolidin-2-ylmethyl)-1H-pyrrolo[3,2-c]quinolin-4-amine hydrochloride **10**

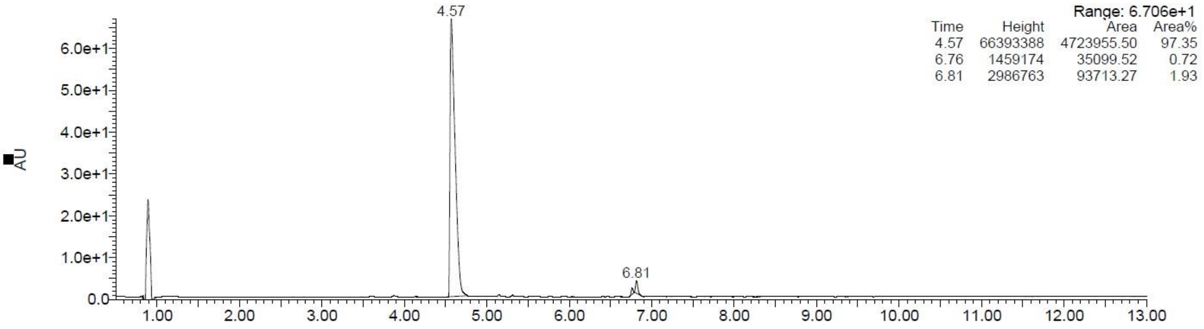

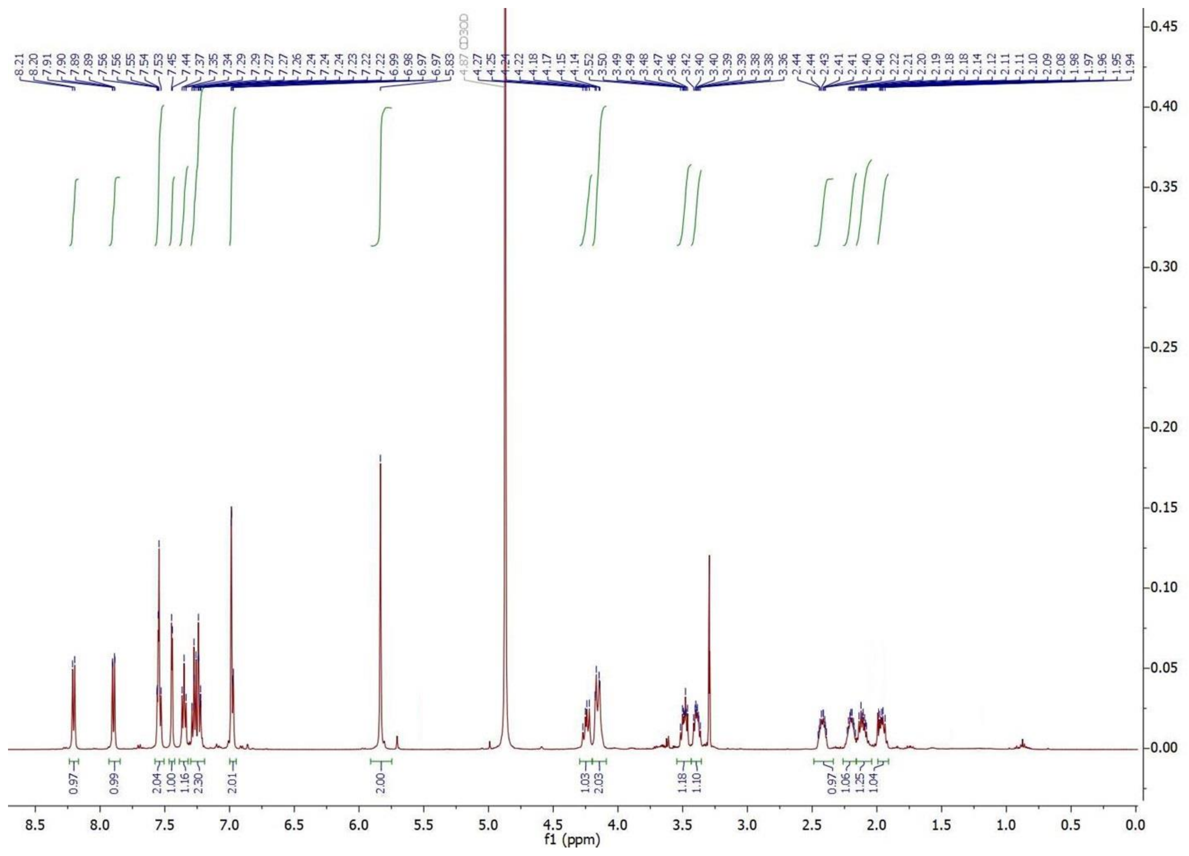

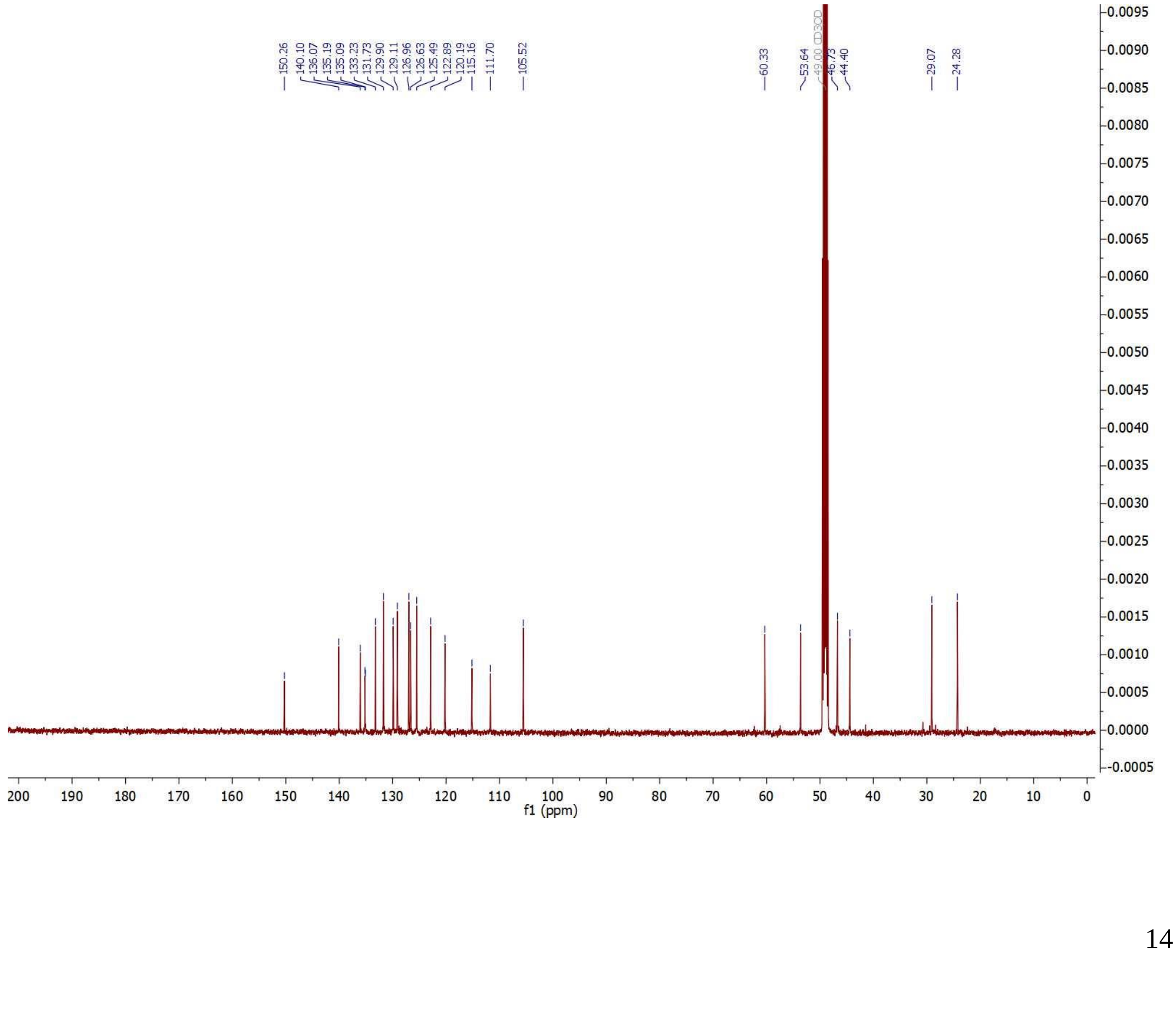

1-(1-(3-Chlorobenzyl)-1H-pyrrolo[3,2-c]quinolin-4-yl)-N-methylazetidin-3-amine hydrochloride **15**

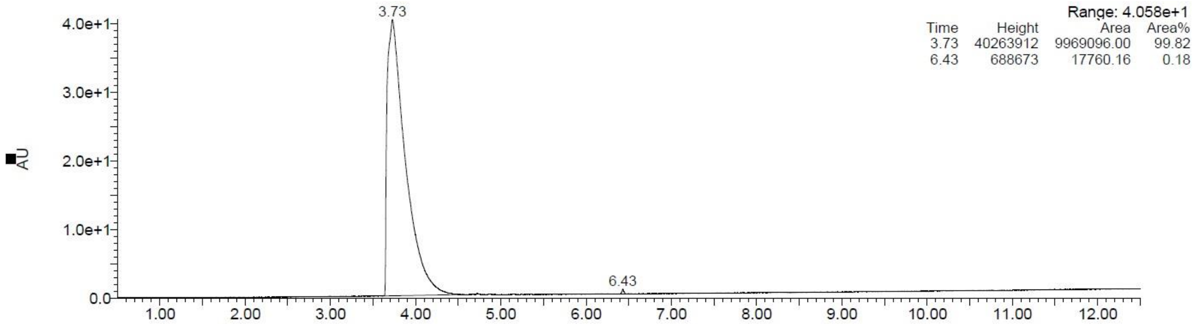

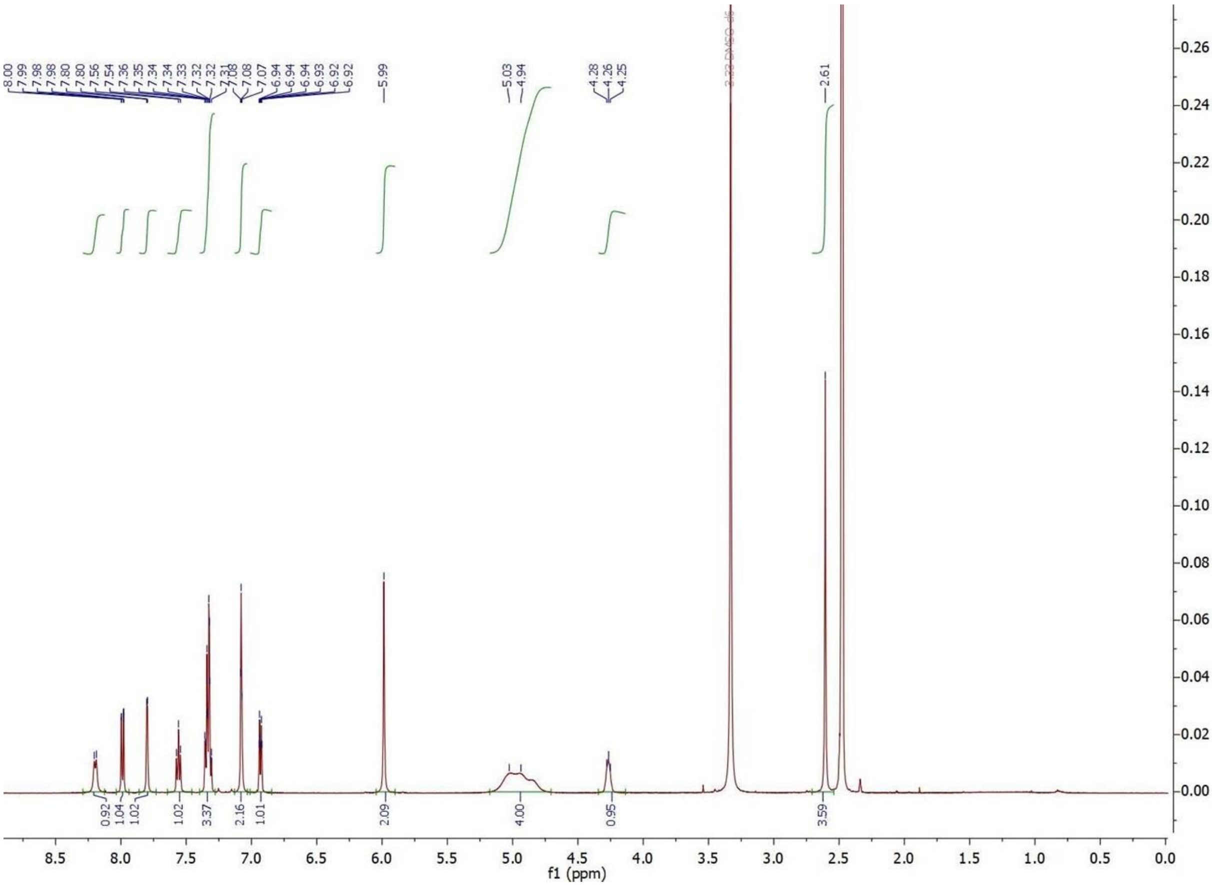

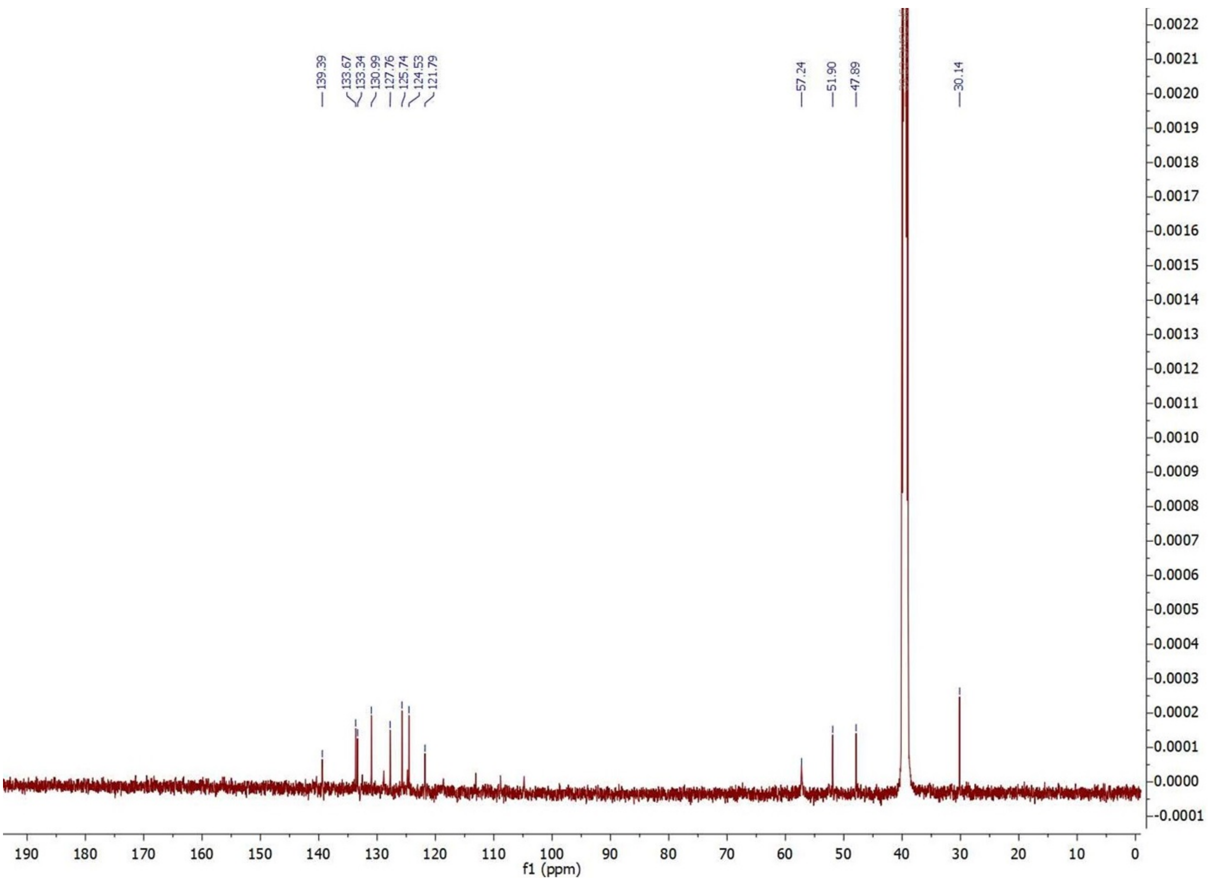

1-(3-Chlorobenzyl)-4-(piperazin-1-yl)-1H-pyrrolo[3,2-c]quinoline hydrochloride **16 (PZ-1922)**

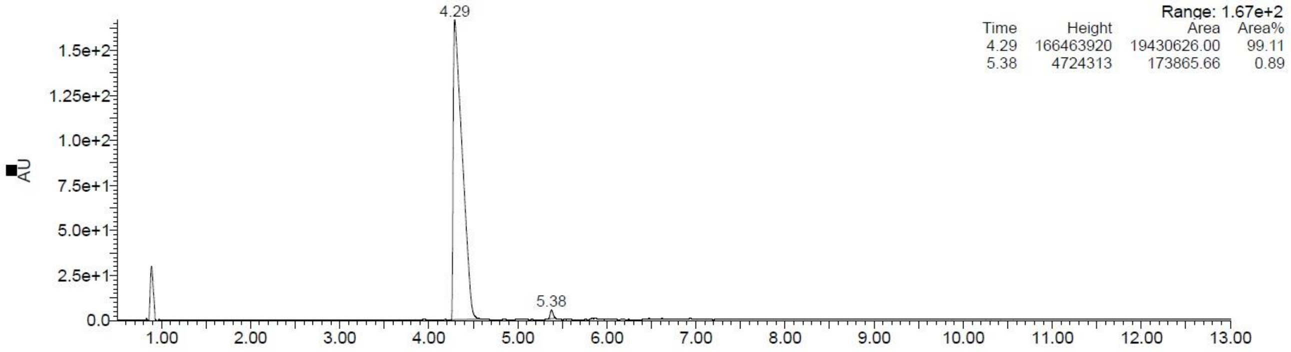

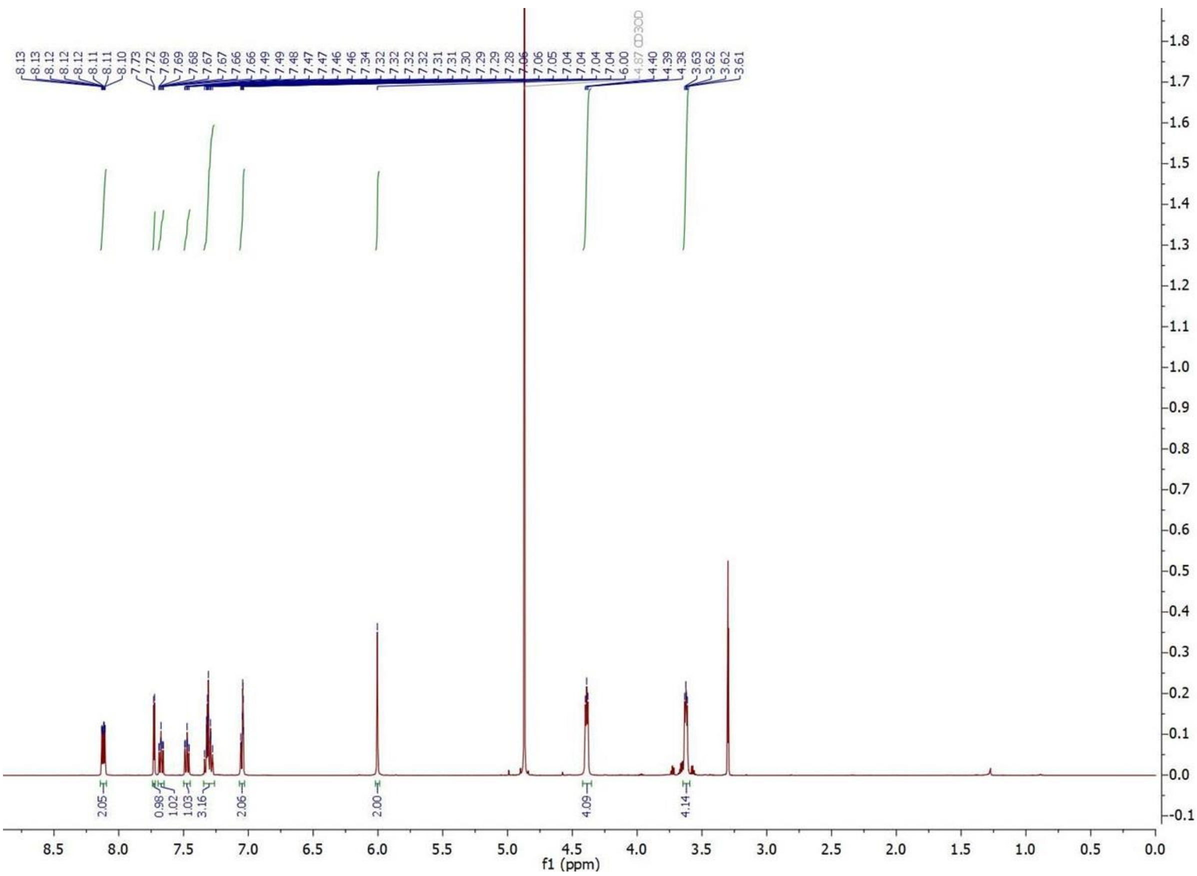
f1 (ppm)

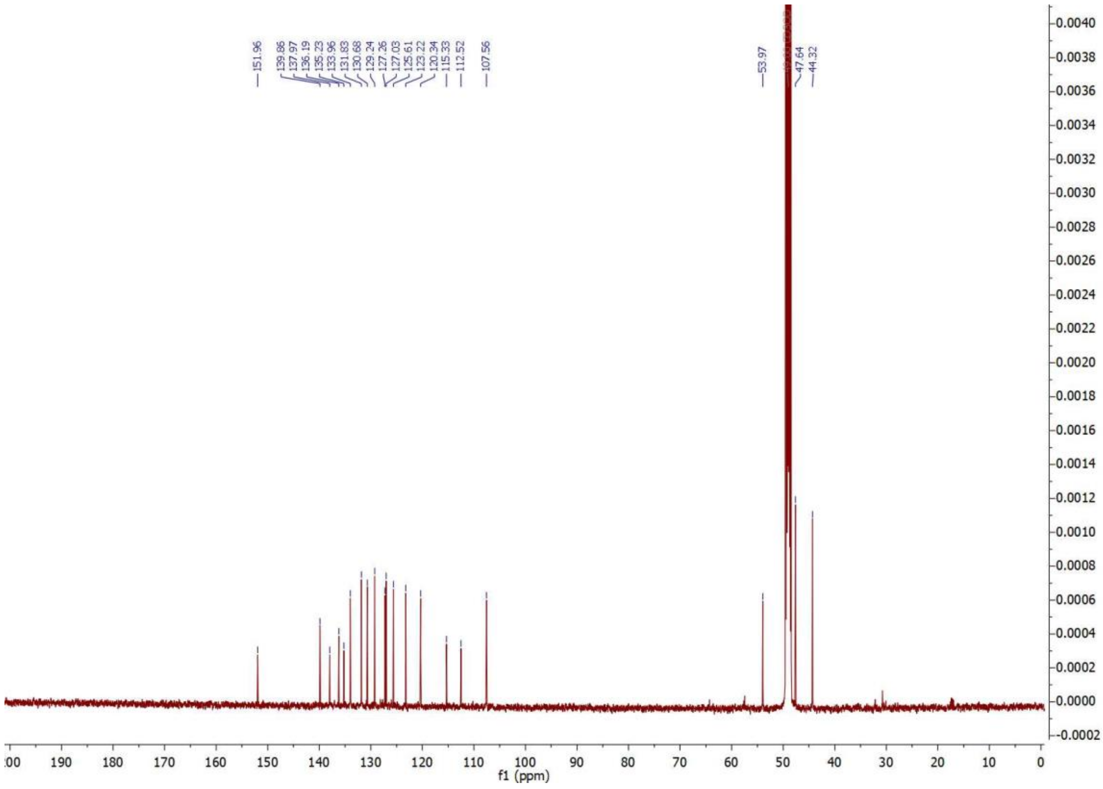
f1 (ppm)

1-(3-Chlorobenzyl)-4-(1,4-diazepan-1-yl)-1H-pyrrolo[3,2-c]quinoline hydrochloride **17**

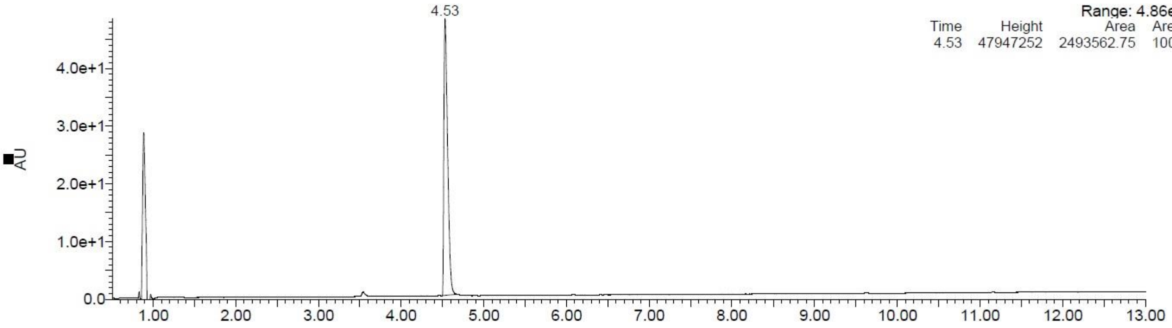

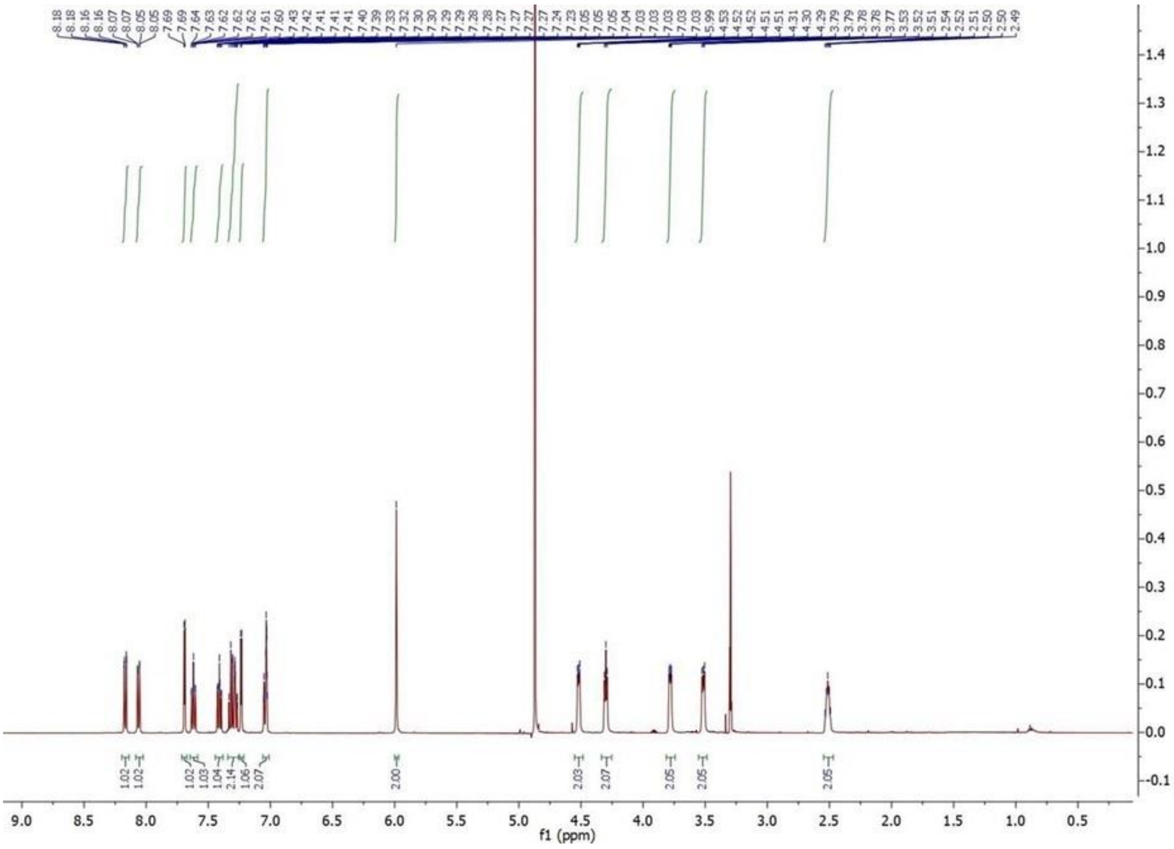

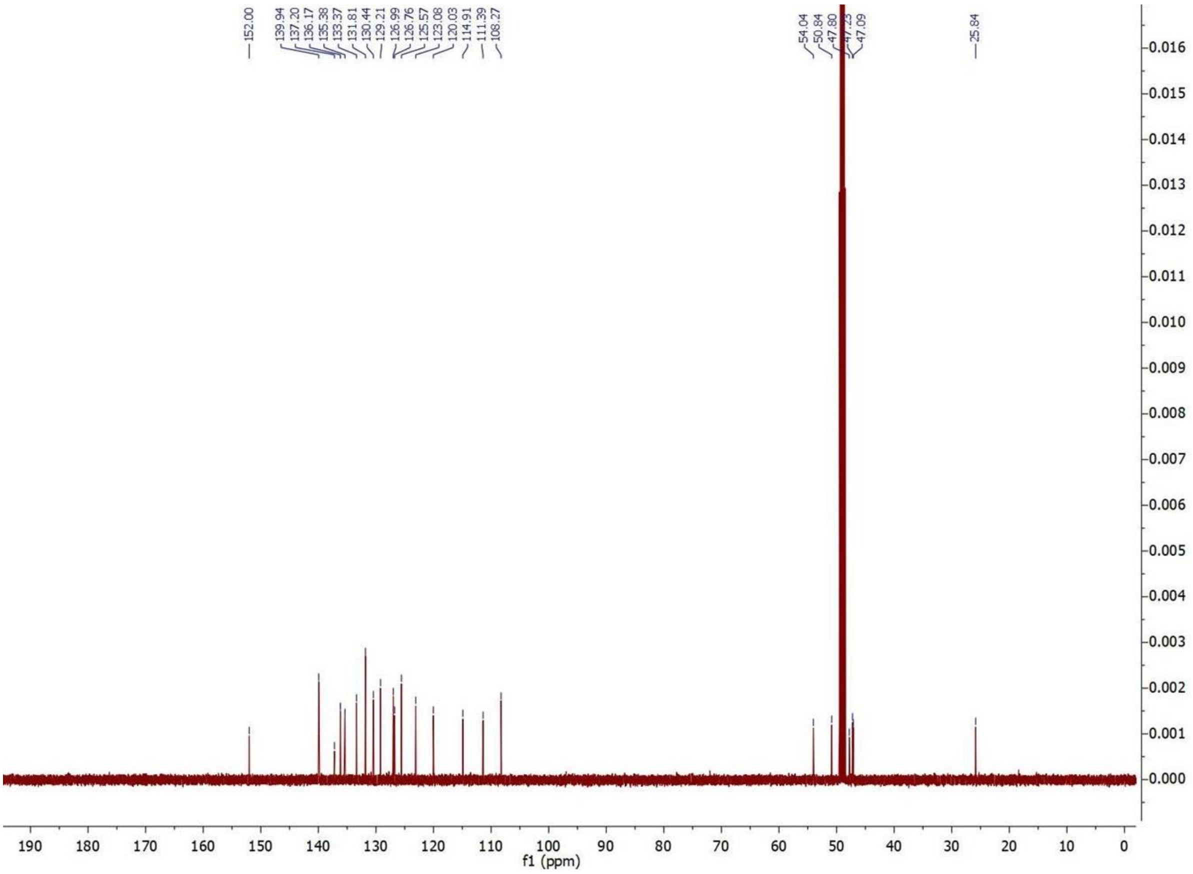

4-((1R,4R)-2,5-Diazabicyclo[2.2.1]heptan-2-yl)-1-(3-chlorobenzyl)-1H-pyrrolo[3,2-c]quinoline hydrochloride **18**

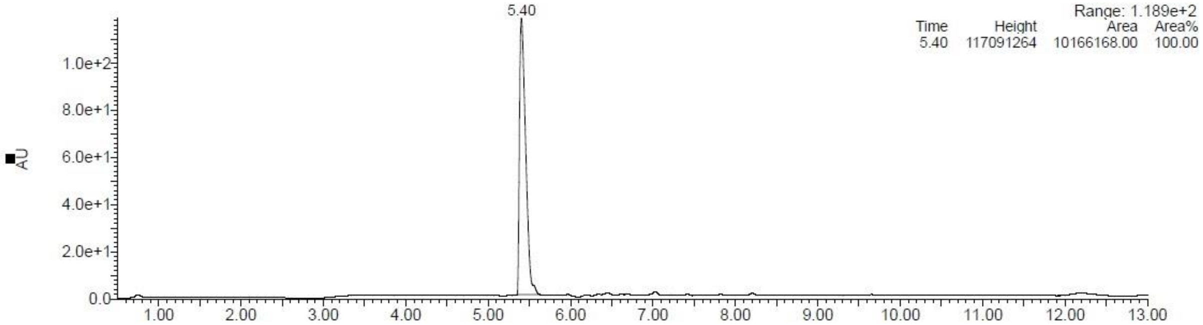

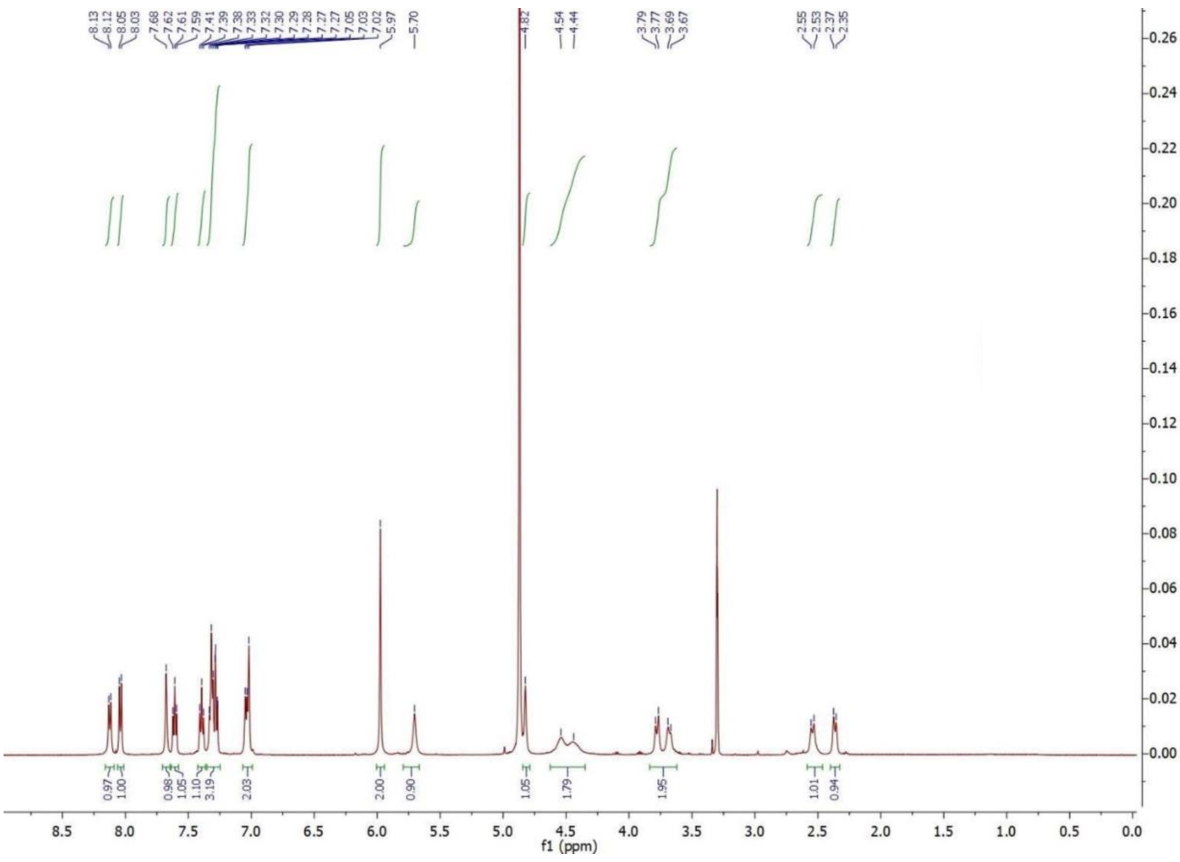

8.13 8.12 8.05 8.03 7.68 7.62 7.61 7.59 7.41 7.39 7.38 7.33 7.32 7.30 7.29 7.28 7.27 7.27 7.05 7.03 7.02 5.97 5.70
4.82
4.54 4.44
3.79 3.77 3.69 3.67
2.55 2.53 2.37 2.35
0.97 1.00 0.98 1.05 1.10 3.19 2.03 2.00 0.90 1.05 1.79 1.95 1.01 0.94
f1 (ppm)

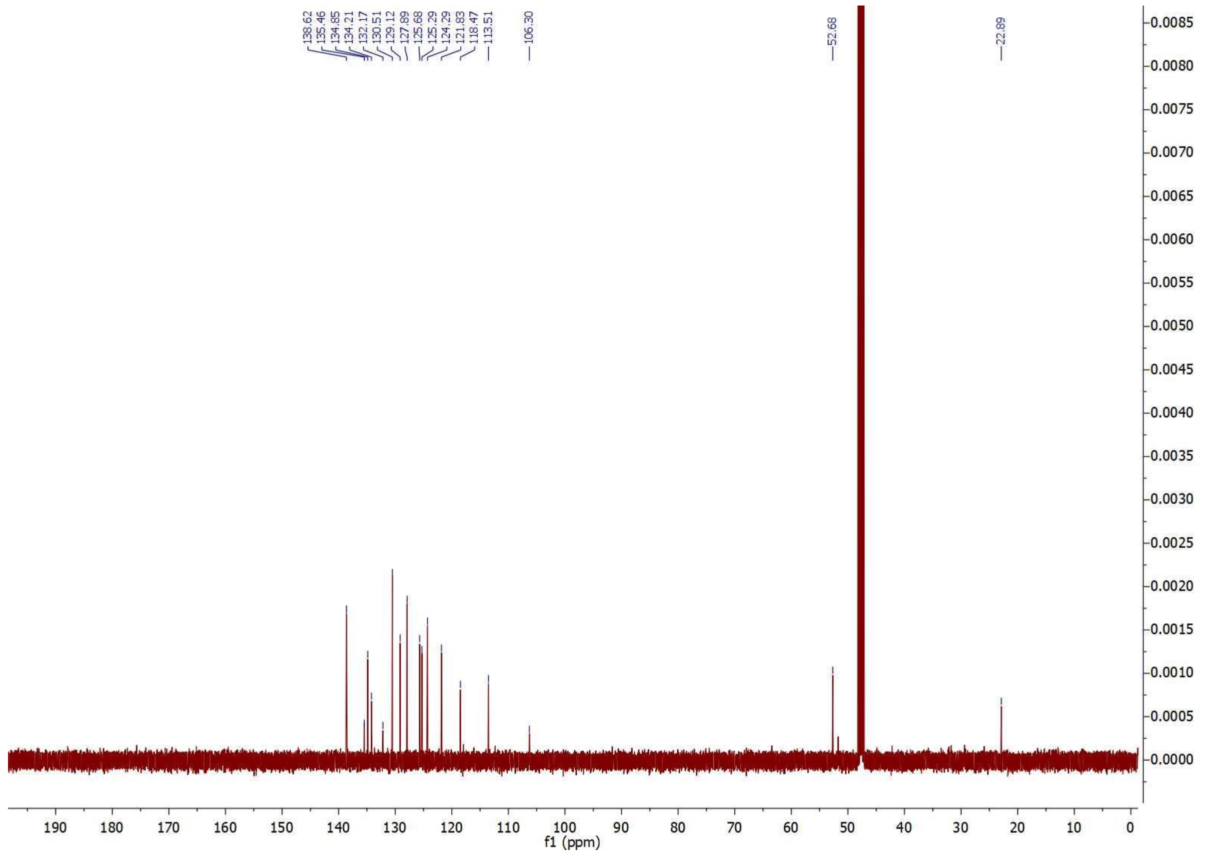

138.62 135.46 134.85 134.21 132.17 130.51 129.12 127.89 125.68 125.29 124.29 121.83 118.47 113.51
106.30
52.68
22.89
f1 (ppm)

1-(3-Chlorobenzyl)-4-(2,6-diazaspiro[3.4]octan-2-yl)-1H-pyrrolo[3,2-c]quinoline hydrochloride **20**

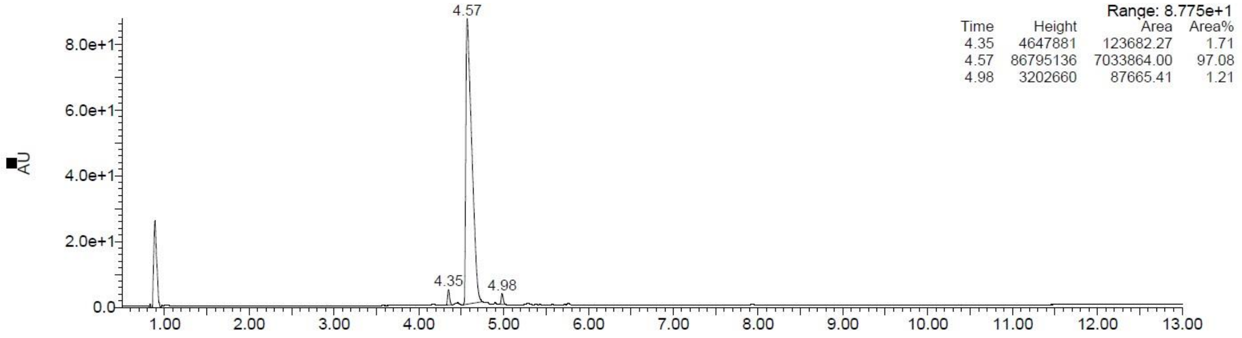

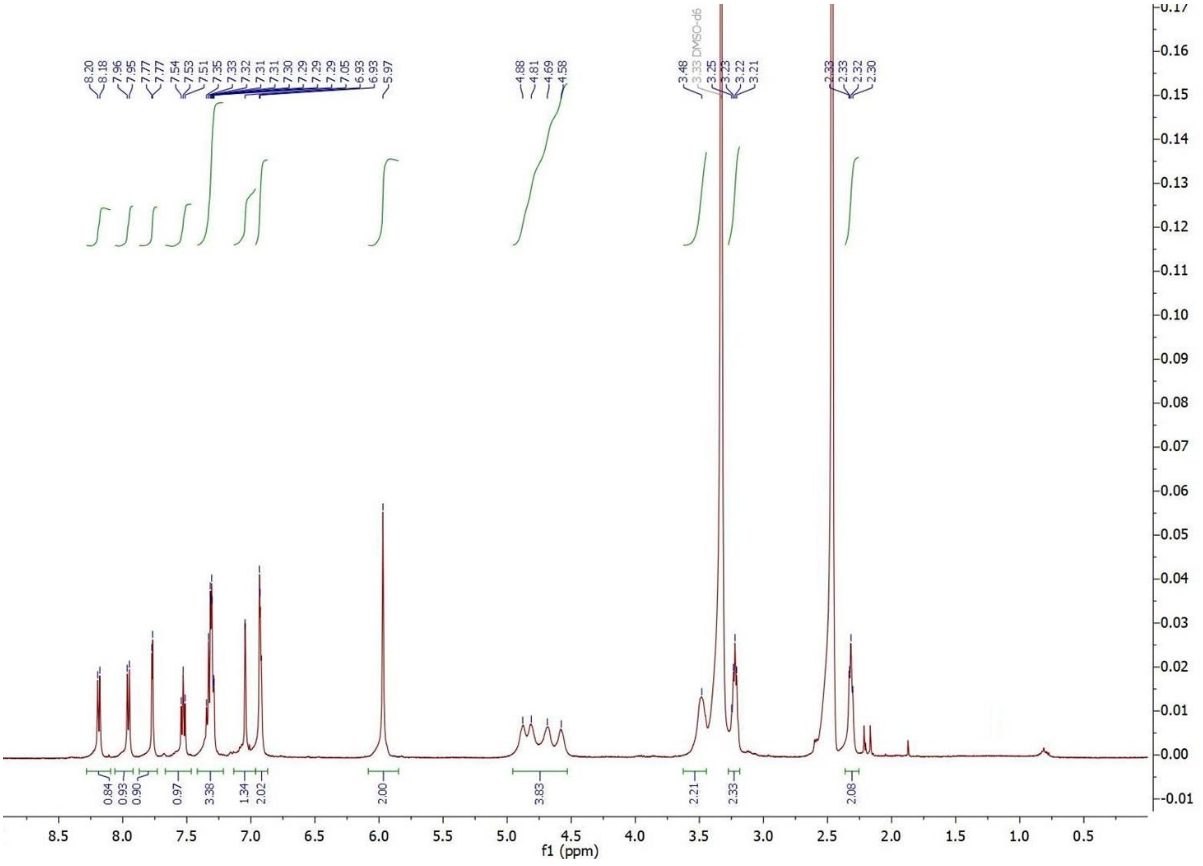

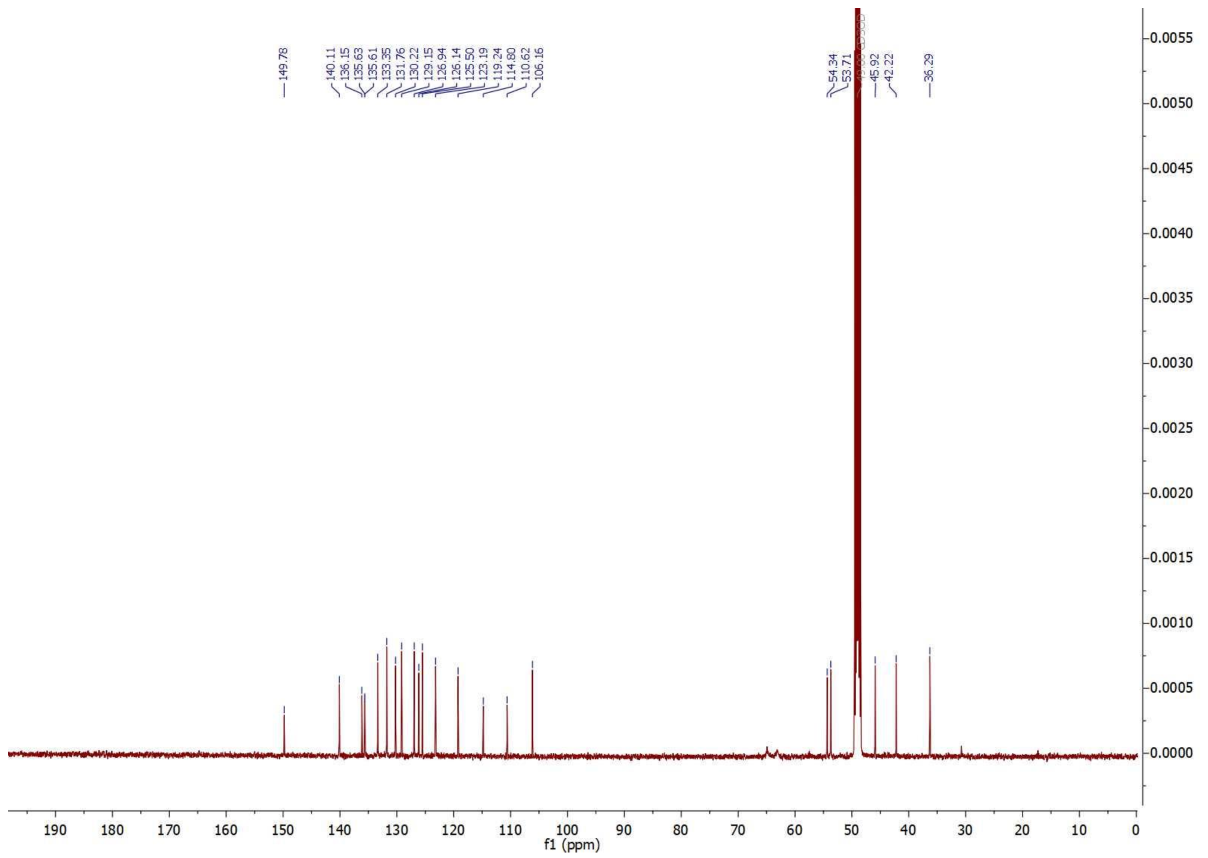

1-(3-Chlorobenzyl)-4-(piperidin-4-yl)-1H-pyrrolo[3,2-c]quinoline hydrochloride **23**

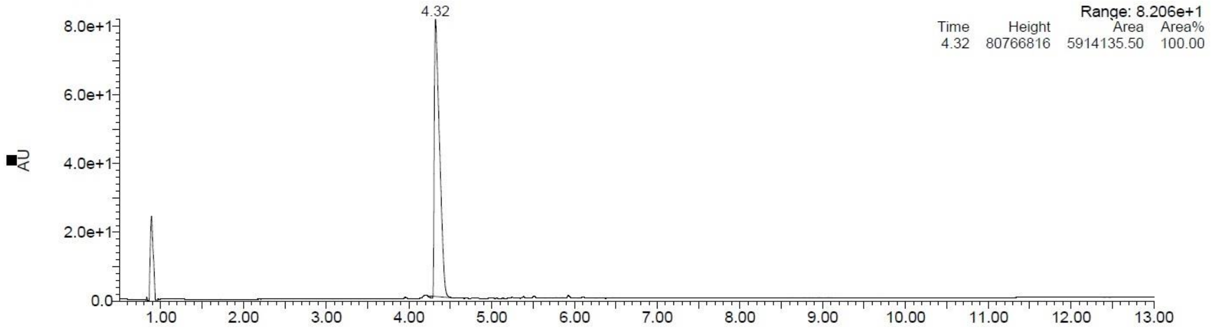

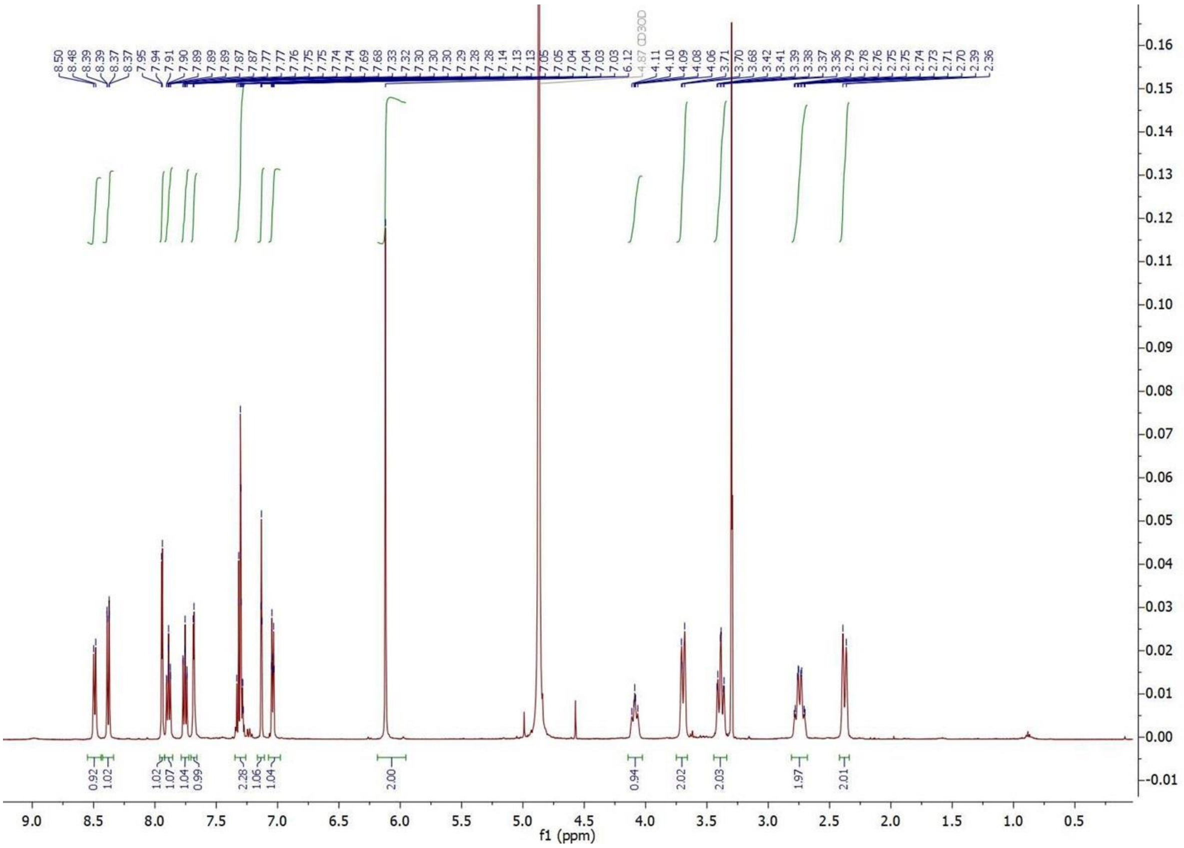
8.50 8.48 8.39 8.39 8.37 8.37 7.95 7.94 7.91 7.90 7.89 7.89 7.87 7.87 7.77 7.77 7.76 7.75 7.75 7.74 7.74 7.69 7.68 7.33 7.32 7.30 7.30 7.30 7.29 7.28 7.28 7.14 7.13 7.13 7.05 7.05 7.04 7.04 7.03 7.03 6.12
4.87 CD3OD
4.11 4.10 4.09 4.08 4.06 3.71 3.70 3.68 3.42 3.41 3.39 3.38 3.37 3.36 2.79 2.78 2.76 2.75 2.75 2.74 2.73 2.71 2.70 2.39 2.36
0.92 1.02 1.02 1.07 1.04 0.99 2.28 1.06 1.04 2.00 0.94 2.02 2.03 1.97 2.01
f1 (ppm)

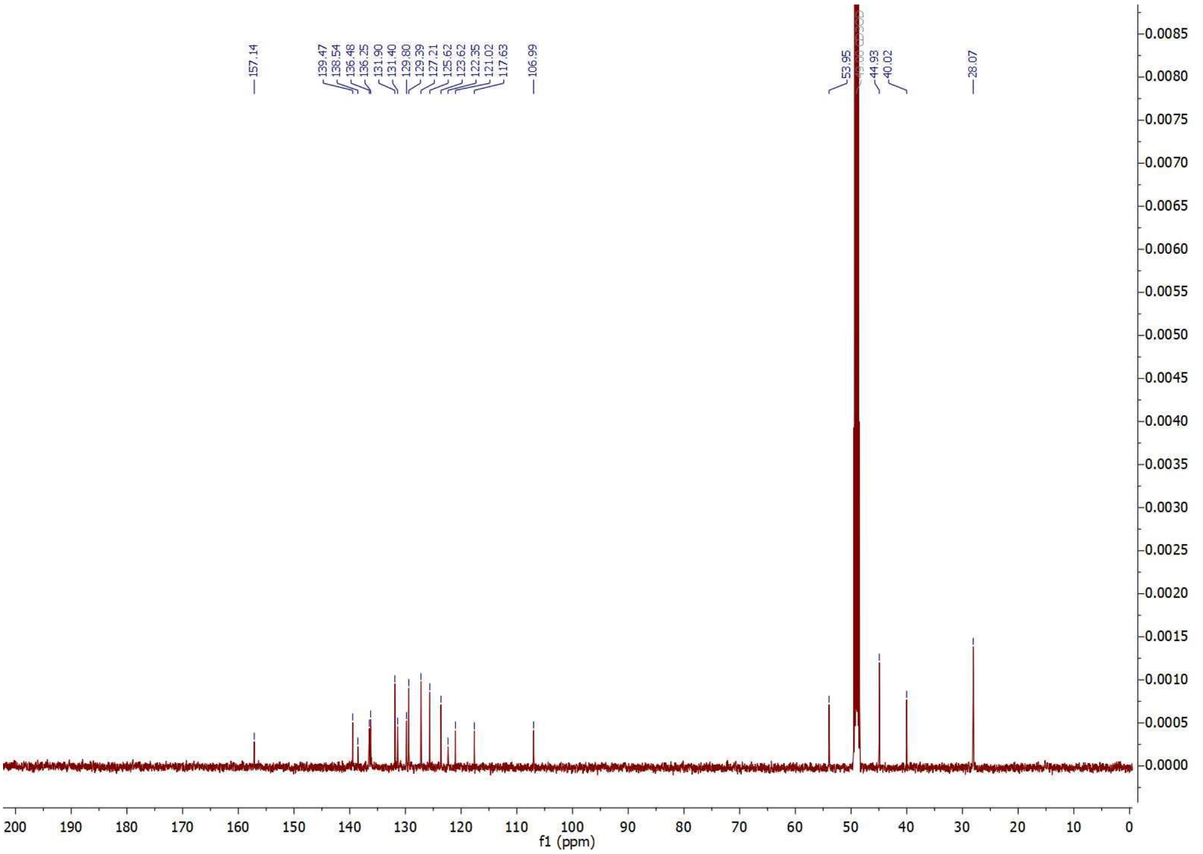
157.14
139.47 138.54 136.48 136.25 131.90 131.40 129.80 129.39 127.21 125.62 123.62 122.35 121.02 117.63
106.99
53.95
44.93
40.02
28.07
f1 (ppm)

2. Assessment of physicochemical parameters of PZ-1922

Calculated descriptors involving molecular weight of 376.89, a LogP of 4.95, a PSA of 33.09 Å2, one hydrogen bond donor, three H-bond acceptors, and three rotable bonds, confirmed the CNS druglike properties of compound **PZ-1922**. Its pK_a equaling for 8.79 indicates that compound will be partially protonated at physiological pH.

PZ-1922 is freely soluble in water (325 mg/mL). It is chemically stable upon acidic and basic hydrolysis (0.5 M HCl and 0.5 M NaOH at 60 °C) and oxidizing conditions (10% H_2O_2 at room temperature).

3. Selectivity screen of PZ-1922

Compound **PZ-1922** was tested for its selectivity over structurally related proteins and off-target receptors.

Inhibition activity for MAO-A was measured using human recombinant MAO-A (Sigma Aldrich M7316) by fluorometric method according the previously reported protocol.[1] The assay was carried out in 96-well plate. 2 mL of appropriate concentration of **PZ-1922** in DMSO were added to wells that contained 98 mL of enzyme dilution (0.53 U/mL) in phosphate buffer (50 mM, pH 7.4). After the 30 min of preincubation in room temperature 50 mL of the solution of 800 mM 10-Acetyl-3,7-dihydroxyphenoxazine (Cayman Chemical Company 10010469) and 4 U/mL horse radish peroxidase (HRP, Sigma Aldrich P6782) was added and enzymatic reaction was started by addition of 50 mL of 800 mM p-tyramine (Alfa Aesar A12220) solution. The signal was measured after 1h (excitation at 570 nm and emission at 585 nm) using EnSpire® multimode plate reader (PerkinElmer Inc.). Clorgyline (1 μM) was tested as reference compound.

Binding assays for serotonin 5-HT_{1A}R, 5-HT_7R and D_2R were performed according to the previously reported methods.[2] HEK293 cells stably expressing human 5-HT_{1A}, 5-HT_{7b} and D_{2L} receptors (prepared with the of Lipofectamine 2000) were maintained at 37 °C in a humidified atmosphere containing 5% CO_2 and grown in Dulbecco's Modified Eagle's Medium containing 10% dialyzed fetal bovine serum and 500 mg/mL G418 sulfate. For membrane preparation, cells were cultured in 150 cm^2 flasks, grown to 90% confluence, washed twice with pre-warmed to 37 °C phosphate buffered saline (PBS) and centrifuged (200 x g) in PBS containing 0.1 mM EDTA and 1 mM dithiothreitol. Prior to membrane preparation, pellets were stored at -80 °C.

Cell pellets were thawed and homogenized in 10 vol of assay buffer using an Ultra Turrax tissue homogenizer and centrifuged twice at 35 000 x g for 15 min at 4 °C, with incubation for 15 min at 37 °C between the centrifugations. The composition of the assay buffers was experimentally selected to achieve the maximum signal window. The assays for 5-HT_7R and D_2Rs were carried out in a total volume of 200 mL in 96-well plates for 1 h at 37 °C. The process of equilibration was terminated by rapid filtration through Unifilter plates with a 96-well cell harvester and radioactivity retained on the filters was quantified on a Microbeta plate reader (PerkinElmer, USA). For displacement studies the assay samples contained as radioligands (PerkinElmer, USA): 2.5 nM [^{3}H]-8-OH-DPAT (135.2 Ci/mmol) for 5-HT_{1A}R, 0.8 nM [^{3}H]-5-CT (39.2 Ci/mmol) for 5- HT_7R or 2.5 nM [^{3}H]-raclopride (76.0 Ci/mmol) for

$D_{2L}R$. Nonspecific binding was defined in the presence of 10 mM of 5-HT in 5-$HT_{1A}R$ and 5-HT_7R binding experiments, whereas 10 mM of haloperidol was used in D_{2L} assay.

Binding assays for serotonin 5-$HT_{2A}R$, 5-$HT_{2B}R$ and 5-$HT_{2C}R$, dopaminergic D_3R, adrenergic α_1R, α_2R and β_1R, muscarinic M_1R, histaminergic H_1R, serotonin transporter SERT and *h*ERG channel were performed at Eurofins, France. The results were expressed as the % inhibition of the control binding according to experimental protocols described online at www.eurofinsdiscovery.com.

For binding assays the following radioligands were used for respective receptors: [^{3}H]-ketanserine for 5-$HT_{2A}R$, [^{3}H]-mesulergine for 5-$HT_{2B}R$ and 5-$HT_{2C}Rs$, [^{3}H]-methylspiperone for D_3R, [^{3}H]-prazosine for α_1R, [^{3}H]-RX821002 for $\alpha_{2A}R$, [^{3}H]-(-)-CGP12177 for β_1R, [^{3}H]-pirenzepine for M_1R, [^{3}H]-pyrilamine for H_1R, [^{3}H]-imipramine for SERT and [^{3}H]-dofetilide for *h*ERG.

4. *In silico* evaluation of PZ-1922 in 5-HT_6R and MAO-B

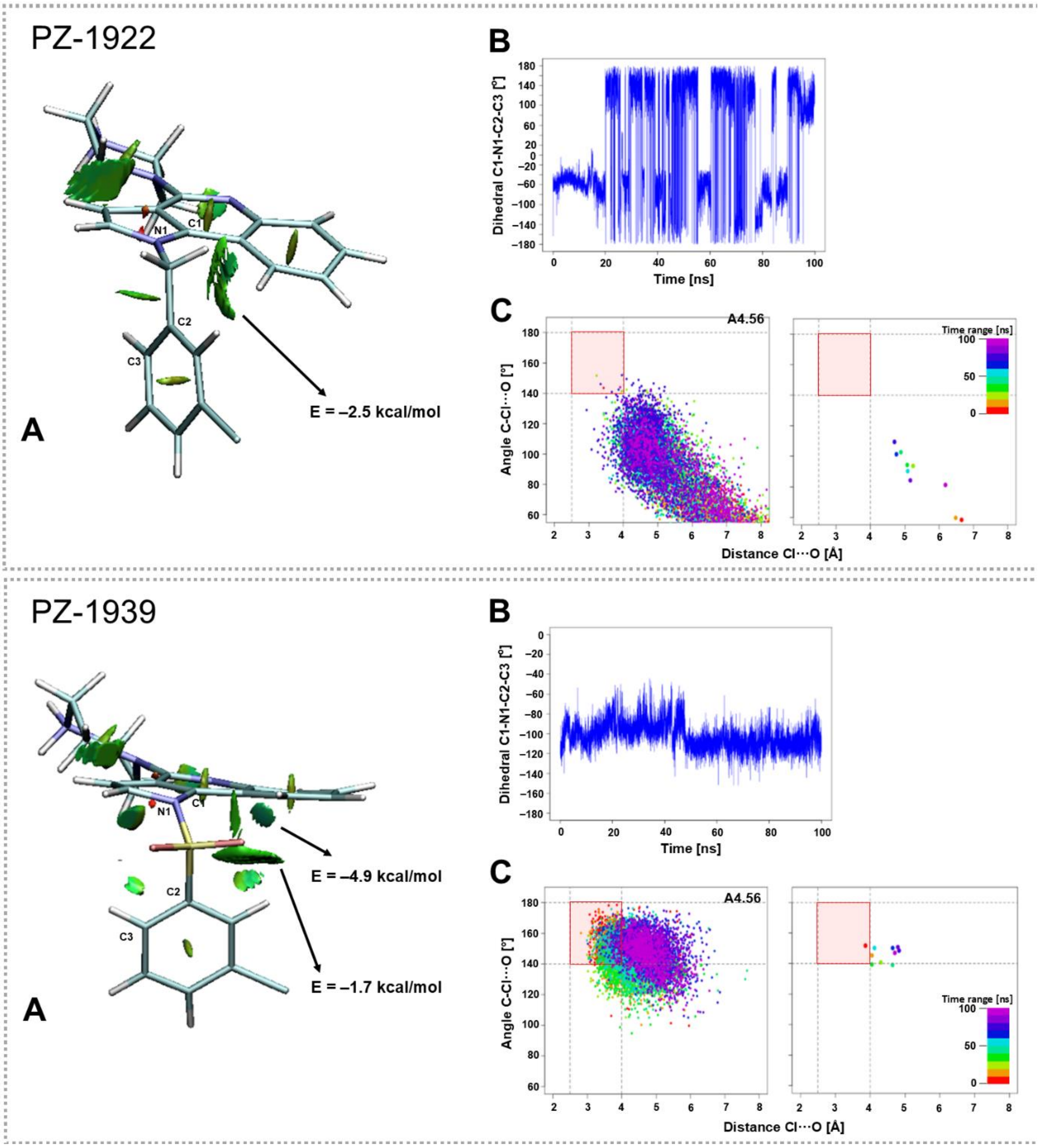

Figure S-1. The QM and MD data analysis results were obtained for **PZ-1922** and **PZ-1939** in the 5-HT_6R. (A) Illustration of the intramolecular interactions *via* the gradient isosurfaces for conformations isolated from the most populated MD cluster. The egg-shaped fields refer to delocalized electrons of aromatic rings and were visualized for gradient surface factor s = 0.1 a.u. (B) Change of the C1-N1-C2-C3 dihedral angle during the MD simulation. (C) The multivariate plot shows the dependence of the distance versus the σ-hole angle for the halogen bond with A4.57 for each trajectory frame, divided into 10 ns-long interval ranges, and the reduced form of the plot presents the medians of the geometric parameters calculated for given time ranges of the trajectory.

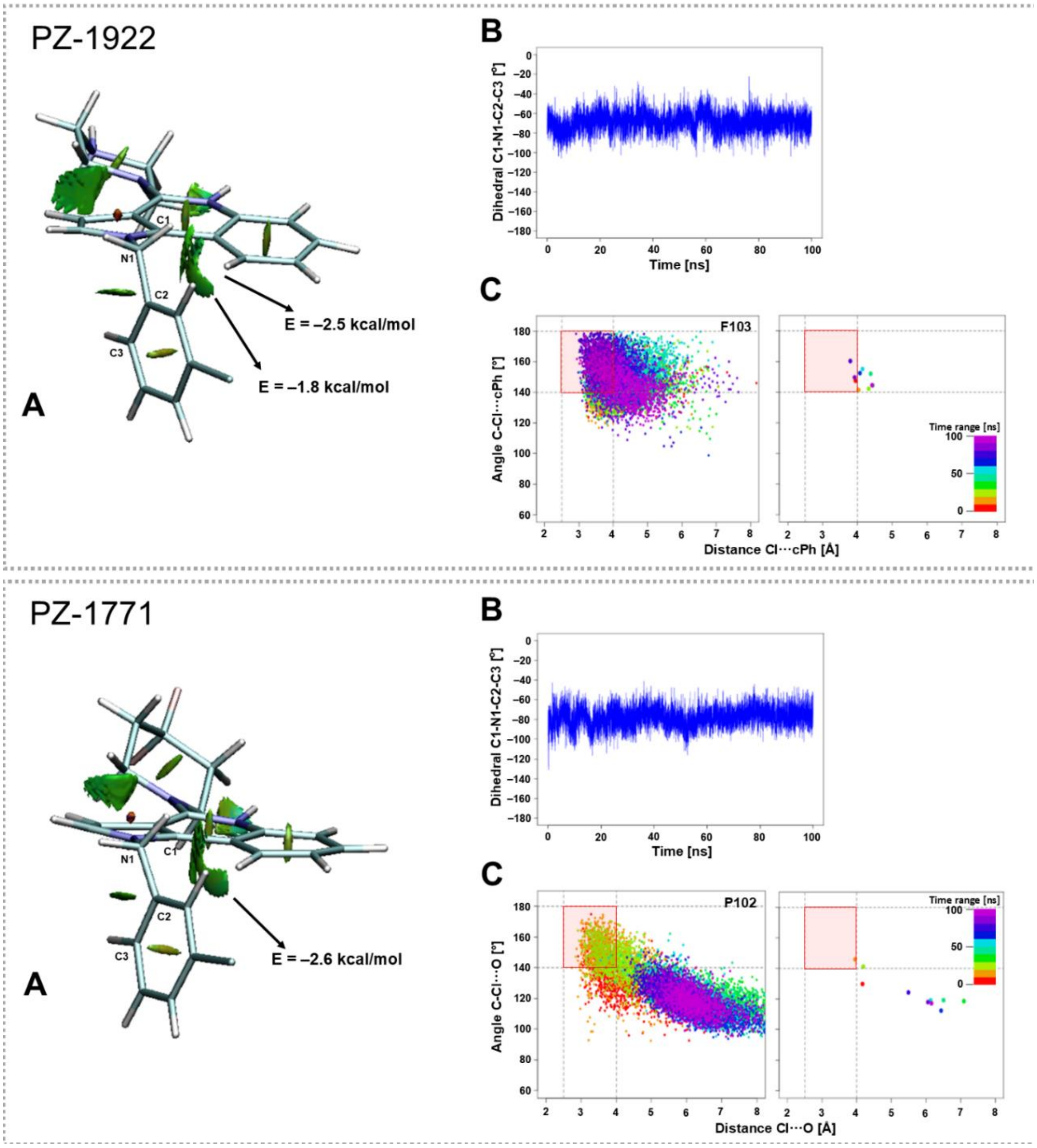

Figure S-2. The QM and MD data analysis results were obtained for **PZ-1922** and **PZ-1771** in the MAO-B enzyme. (A) Illustration of the intramolecular interactions via the gradient isosurfaces for conformations isolated from the most populated MD cluster. The egg-shaped fields refer to delocalized electrons of aromatic rings and were visualized for gradient surface factor s = 0.1 a.u. (B) Change of the C1-N1-C2-C3 dihedral angle during the MD simulation. (C) The multivariate plot shows the dependence of the distance versus the σ-hole angle for the halogen bond with ring centroid of F103 (for **PZ-1922**), and P102 (for **PZ-1771**) for each trajectory frame, divided into 10 ns-long interval ranges, and the reduced form of the plot presents the medians of the geometric parameters calculated for given time ranges of the trajectory.

5. Cryo-EM studies on PZ-1922 at 5-HT3R

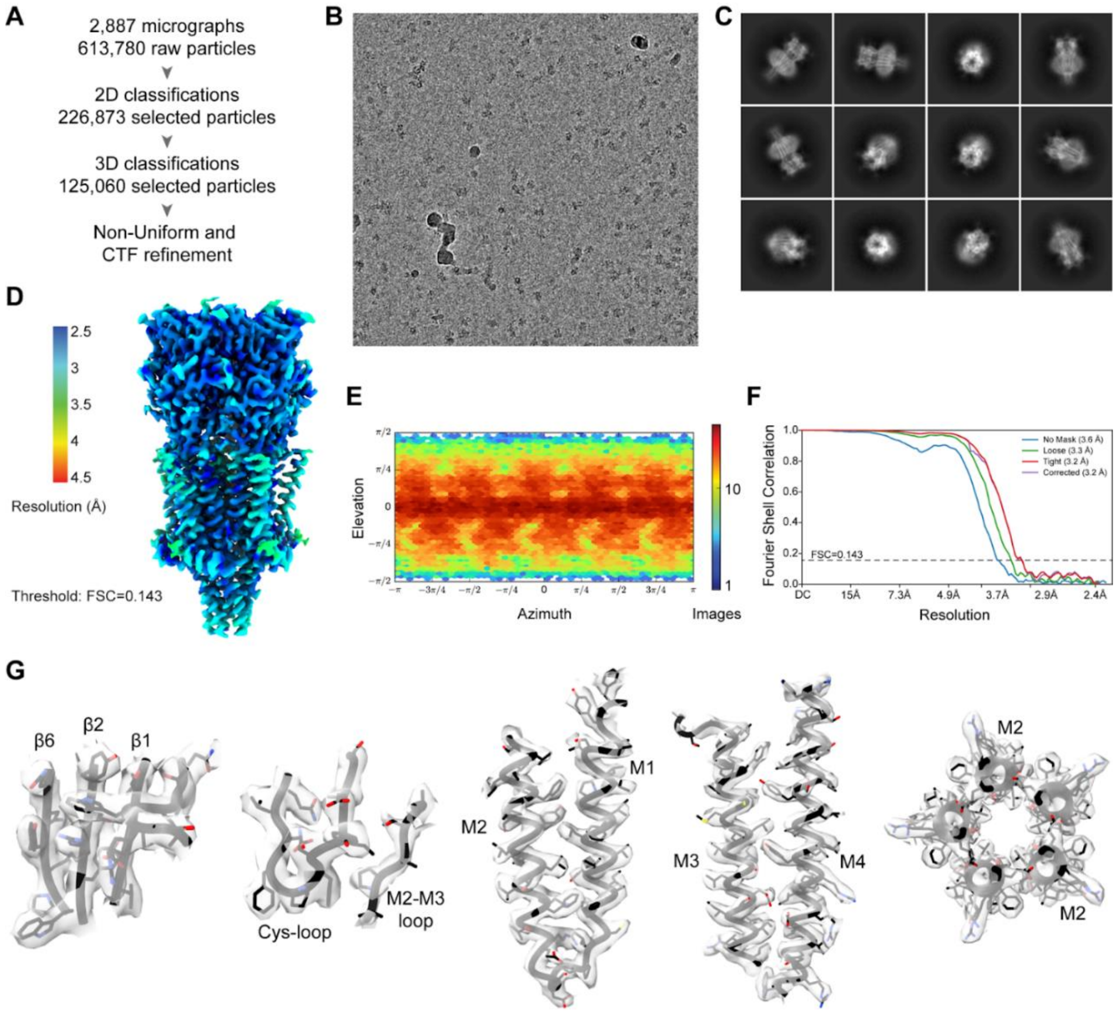

Figure S-3. Image analysis workflow and quality density maps of the m5-HT3AR in complex with **PZ-1922**. (A) Schematic of the image analysis workflow (B) A representative micrograph of the dataset. (C) Selected 2D class averages of the final particles set. (D) Side view of the final reconstruction. The sharpened 3D density map is colored according to the local resolution (FSC threshold of 0.143). (E) Heat map of the angular distribution of particle projections for the reconstruction. (F) Gold-standard FSC curves. The dotted line represents the 0.143 FSC threshold. (G) Densities of the reconstructions in surface representation overlaid with the structure. From left to right: densities of the β-sheets in the ECD, densities of the Cys-loop and the M2–M3 loop, densities of helices M1 and M2, densities of M3 and M4, densities of M2 at the level of L9′ (L260).

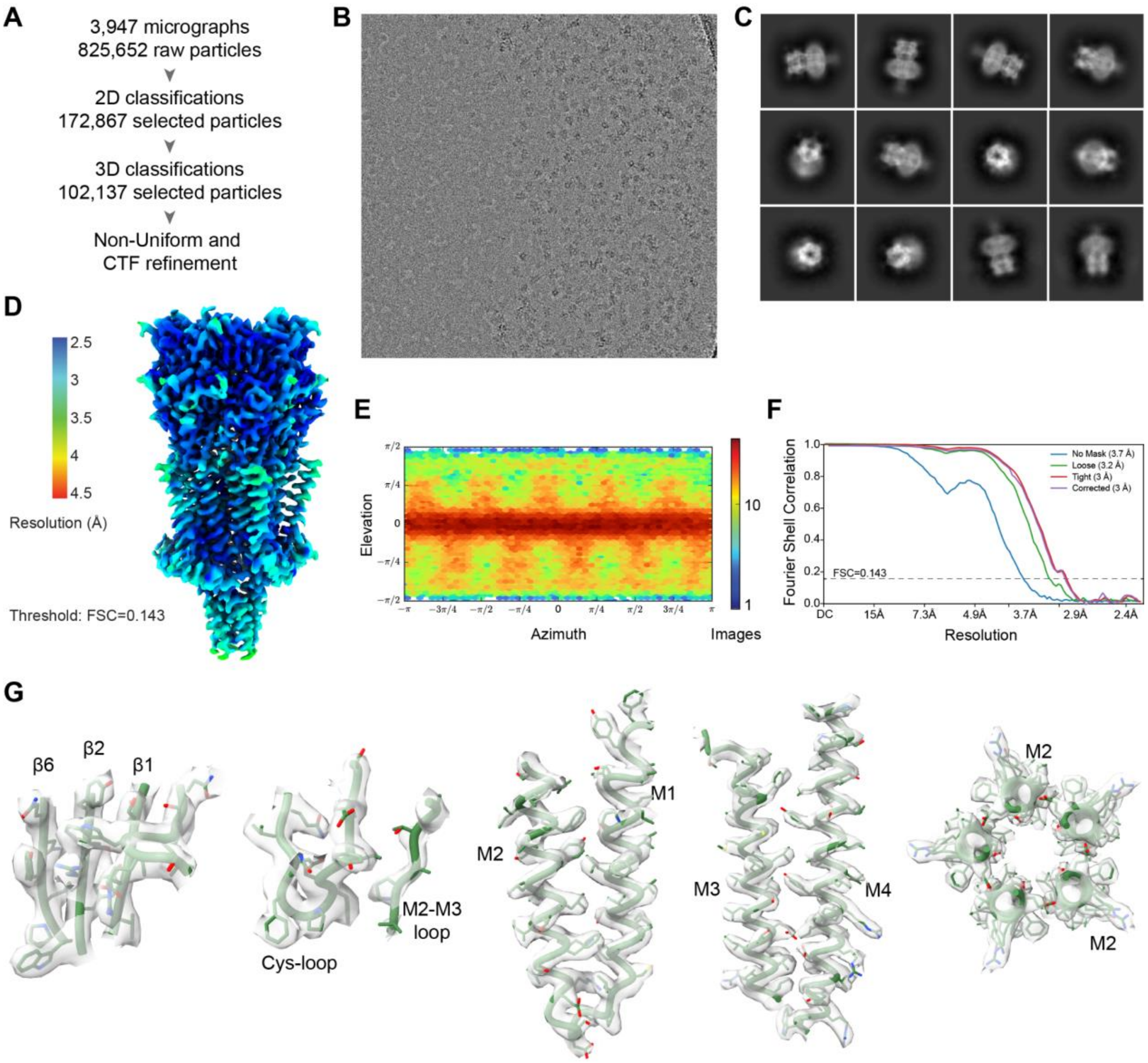

Figure S-4. Image analysis workflow and quality density maps of the m5-HT3AR in complex with **PZ-1939**. (A) Schematic of the image analysis workflow (B) A representative micrograph of the dataset. (C) Selected 2D class averages of the final particles set. (D) Side view of the final reconstruction. The sharpened 3D density map is colored according to the local resolution (FSC threshold of 0.143). (E) Heat map of the angular distribution of particle projections for the reconstruction. (F) Gold-standard FSC curves. The dotted line represents the 0.143 FSC threshold. (G) Densities of the reconstructions in surface representation overlaid with the structure. From left to right: densities of the β-sheets in the ECD, densities of the Cys-loop and the M2–M3 loop, densities of helices M1 and M2, densities of M3 and M4, densities of M2 at the level of L9′ (L260).

Table S-1. Cryo-EM data collection, refinement and validation statistics.

	**m5-HT_3A-PZ-1922**	**m5-HT_3A-PZ-1939**
EMDB	8CC6	8CC7
PDB	EMD-16555	EMD-16557
**Data collection & processing**		
Microscope	Glacios-IBS	Glacios-IBS
Magnification	36,000	36,000
Voltage (kV)	200	200
Frames	40	40
Total movies (no.)	2887	3947
Electron dose (e-/Å^2)	40	40
Defocus range (µm)	-0.7 to –2.2	-0.7 to –2.2
Collection mode	Counting	Counting
Effective pixel size (Å)	1.145	1.145
Initial particle images (no.)	613,780	825,652
Final particle images (no.)	125,060	102,137
Symmetry imposed	C5	C5
B-factor sharpening	141	100
Map resolution (Å)[a] FSC threshold 0.143	3.2	3.0
**Model refinement**		
Initial model used (PDB code)	m5-HT_3A-palonosetron (6Y1Z)	m5-HT_3A-palonosetron (6Y1Z)
**Model composition**		
Non-hydrogen atoms	16,640	16,745
Protein residues	1,950	1,950
**B factors** (min/max/mean Å^2)		
Protein		
Ligand	0/109/49	47/170/105
	14/80/50	97/148/119
**R.M.S. Deviations**		
Bond lengths (Å)		
Bond angles (°)	0.03	0.02
	0.546	0.530
**Validation**		
MolProbity score		
Clashscore	1.18	1.28
Rotamer outliers (%)	3.99	5.21
	0.11	0.66
**Ramachandran plot** (%)		
Favored		
Allowed	98.03	98.6
Outliers	1.97	1.4
	0	0
Model Resolution[b]	3.3	3.2

[a] Resolution determined by Gold-Standard FSC threshold of 0.143 for sharpened masked map.

[b] Resolution determined by FSC threshold of 0.5 for sharpened masked map.

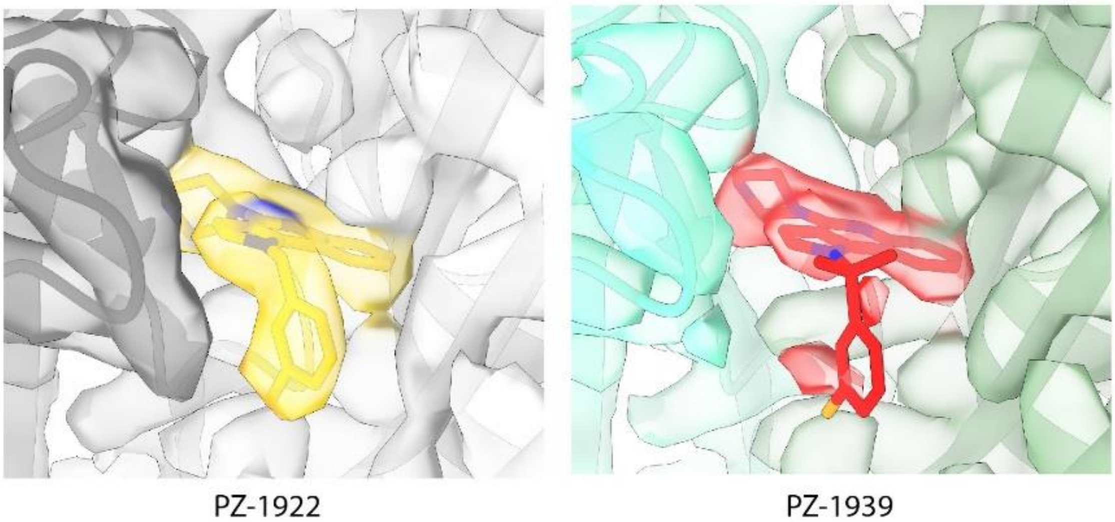

Figure S-5. Comparison of ligand densities for **PZ-1922** and **PZ-1939**. The Cryo-EM reconstructions are represented side by side **PZ-1922** (left, yellow) and **PZ-1939** (right, red) bound to the receptor. The ligand is also represented as sticks. While the densities are equivalent for the piperazine ring (in the background) and the 1*H*-pyrrolo[3,2-*c*]quinoline system, a clear difference is observed for the 3-chlorobenzyl and 3-chlorobenzenesulfonyl moieties at the entrance of the binding site. The density is less defined and broken for **PZ-1939**, indicative of the flexibility of this cycle. We hypothesize that this absence of ordering could be due to the two additional oxygen atoms that prevent a correct capping of the protein loop C that would immobilize the ligand.

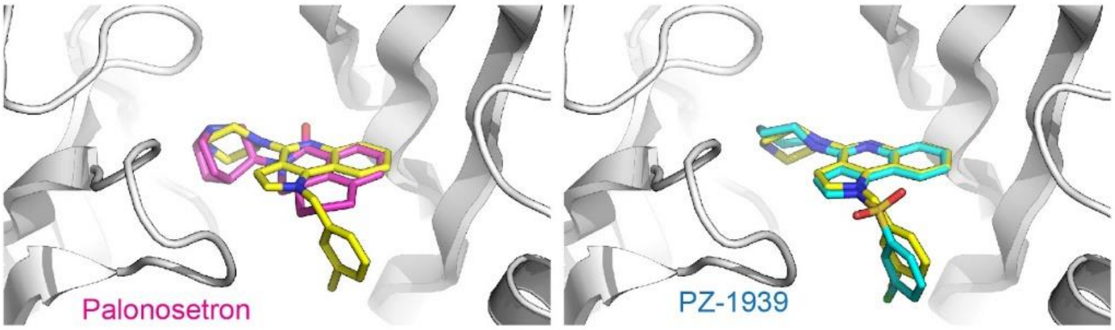

Figure S-6. Comparison of ligand poses for **PZ-1922** with palonosetron and **PZ-1939**. The panels depict the overlays of **PZ-1922** with palonosetron (yellow and pink) or **PZ-1922** with **PZ-1939** (yellow and cyan).

Interpretation: the most outer cycle of both **PZ-1922** and **PZ-1939** is probably not of the primary importance for affinity at the 5-HT_3 receptor.

6. Pharmacokinetic evaluation of PZ-1922

Table S-2. Pharmacokinetic parameters and brain uptake for **PZ-1922** after intravenous and intragastric administration to rats at a dose of 3 mg/kg.

Parameters	Intravenous		Intragastric	
	Plasma	Brain	Plasma	Brain
C_0 [ng/mL]; [ng/g]	244.3	293.6	-	-
$AUC_{0\to t}$ [ng × min/mL]; [ng × min/g]	28000	170827	13436	42186
$t_{0.5}$ [min]	755	243	362	439
MRT [min]	209	170	236	275
C_{max} [ng/mL]; [ng/g]	-	-	38.6	129.6
t_{max} [min]	-	-	120	240
V_{ss} [mL/kg]	42011	-	-	-
Cl [mL/min/kg]	39.2	-	-	-
F [%]	48			

C_0 – initial concentration; AUC - area under the curve; $t_{0.5}$ – terminal half life; C_{max} - maximum plasma concentration; t_{max} - time to reach C_{max}; V_{ss} – volume of distribution; Cl – clearance; MRT – mean residence time, F – bioavailability

Brain/Plasma ratio after *iv* administration = 6.10

Brain/Plasma ratio after *ig* administration = 3.14

Instrumentation and operating conditions

The LC/ESI-MS/MS experiments were performed on a triple quadrupole mass spectrometer (API 3200, Sciex, USA) equipped with an electrospray (ESI) ionization interface and coupled to HPLC system (Shimadzu, Japan). Data acquisition and processing were accomplished using Analyst 1.5.2. data collection and integration software.

Chromatographic conditions

The chromatographic separation was performed on XBridge C18 (2.1 µm, 2.1 x 50 mm, Waters, USA) column with the column temperature set at 30˚C. The mobile phase consisted of 0.1% formic acid in water (solvent A, 60%) and 0.1% formic acid in acetonitrile (solvent B, 40%) in isocratic elution set at a flow rate of 0.6 mL/min.

Mass spectrometry conditions

The mass spectrometer parameters were as follows: ion spray voltage: 5000 V; temperature of the heated nebulizer: 400°C and collision energy 40 kV. Mass spectra were acquired by SRM

with precursor/predominant product ion transitions for the analytes. The mass spectral Q1→Q3 transitions monitored for **PZ-1922** were m/z 377.1→334.1; 377.1→308.2 and 377.1→209.2 and for internal standard (IS) was 305.03→248.09. The peak widths of precursor and product ions were set to 0.7 full width half-height. Quantification was done *via* peak area ratio.

Pharmacokinetic study - calculation

The area under the mean plasma and brain concentration versus time curve (AUC0→t) was calculated from zero to the last concentration point using the linear trapezoidal rule as:

$$\mathrm{AUC}_{0\to t} = \sum_{i=1}^{n} \frac{C_i + C_{i+1}}{2} \cdot (t_{i+1} - t_i) \qquad (1)$$

where C_i is the concentration of the compound.

The area under the first-moment curve (AUMC0 ®t) was estimated by calculation of the total area under the first-moment curve:

$$\mathrm{AUMC}_{0\to t} = \sum_{i=1}^{n} ((t_i \cdot C_i + t_{i+1} \cdot C_{i+1})/2) \cdot (t_{i+1} - t_i) \qquad (2)$$

where t_i is the time of the last sampling.

Mean residence time (MRT) was calculated as:

$$\mathrm{MRT} = \frac{\mathrm{AUMC}_{0\to t}}{\mathrm{AUC}_{0\to t}} \qquad (3)$$

Total clearance (Cl) was calculated as:

$$\mathrm{Cl} = \frac{\mathrm{D_{iv}}}{\mathrm{AUC}_{0\to t}} \qquad (4)$$

Volume of distribution at steady-state (V_{ss}) was calculated as:

$$\mathrm{V_{ss}} = \frac{\mathrm{D_{iv}} \cdot \mathrm{AUMC}_{0\to t}}{(\mathrm{AUC}_{0\to t})^2} \qquad \text{(Eq. 5)}$$

The absolute bioavailability after intragastric administration was calculated as:

$$\mathrm{F}(\%) = \frac{\mathrm{AUC_{ig}}}{\mathrm{AUC_{iv}}} \cdot \frac{\mathrm{D_{iv}}}{\mathrm{D_{ig}}} \cdot 100 \qquad \text{(Eq. 6)}$$

where D_{iv} and D_{ig}. are *iv* and *ig* doses of **PZ-1922**, respectively.

7. Results of the biochemical analysis of the impact of PZ-1922 and intepirdine on apoptotic processes in the curative and preventive treatment

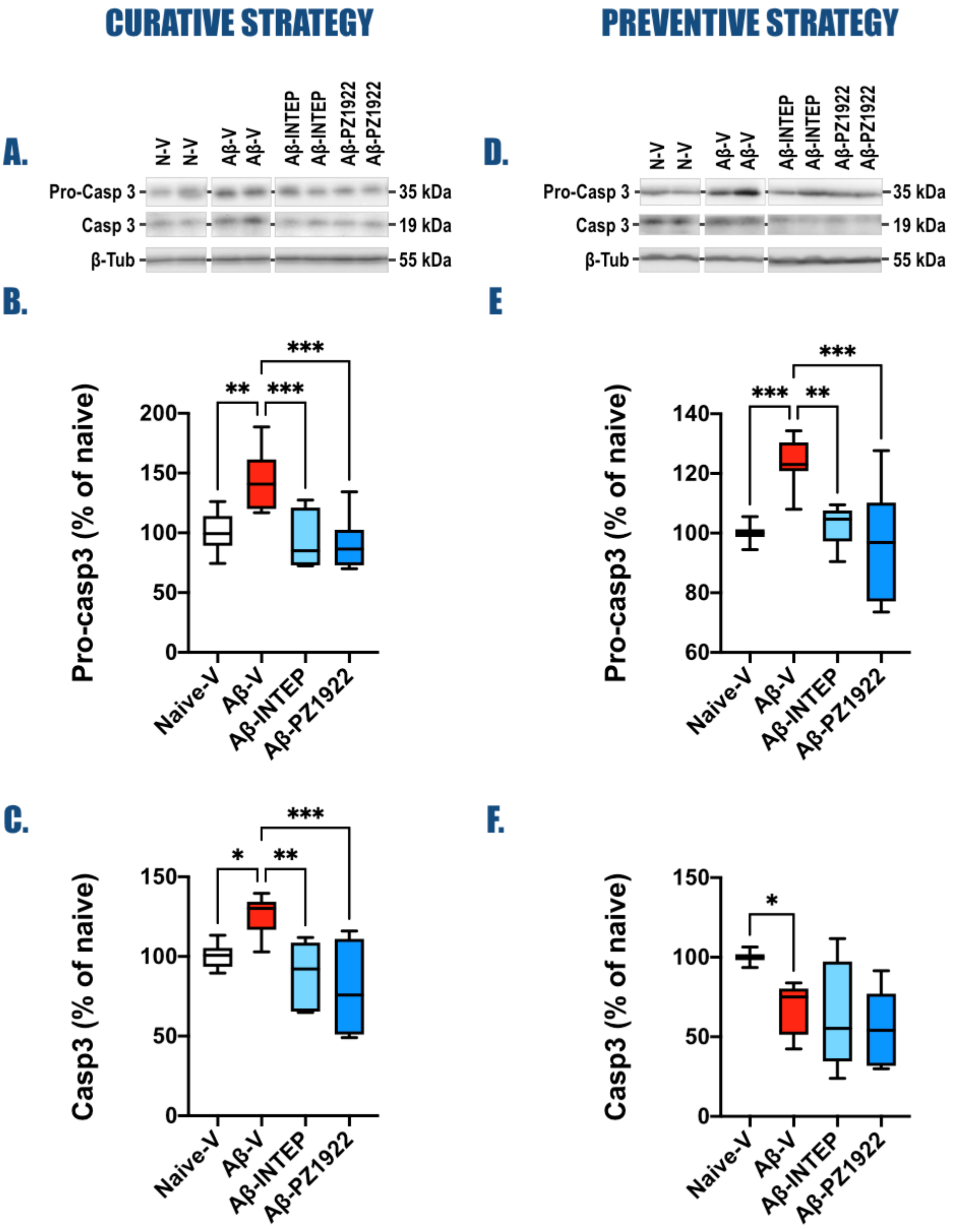

Figure S-7. The impact in the hippocampus of the curative (A-C) and preventive (D-F) strategies with intepirdine (INTEP) and **PZ-1922** on apoptotic processes induced by the *icv* injection of $A\beta_{25\text{-}35}$ were evaluated by Western blot. Variations of pro-caspase 3 (Pro-casp 3, 35 kDa) (**A,B,D,E**) and clived caspase 3 (Casp 3, 19 kDa) (**A,C,D,F**) were evaluated in each group, normalized with the variations of β-tubulin (β-tub, 55 kDa) and expressed in percent of variations obtained in non-injected rats (Naive group). For experimental protocols see Figure 6A and Figure 6G. All data are presented as box & whiskers with Min to Max and Median with n = 8 for Naive-V and Aβ-V groups; and n = 6 for Aβ-INTEP and Aβ-**PZ-1922** groups. One-way ANOVA followed by Dunnett's multiple comparison was performed (See Table S-5). * p< 0.05; *** p< 0.001 and **** p< 0.0001 *vs* $A\beta_{25\text{-}35}$ group treated with vehicle (Aβ-V).

8. Results of the biochemical analysis of curative treatment with PZ-1922 and intepirdine

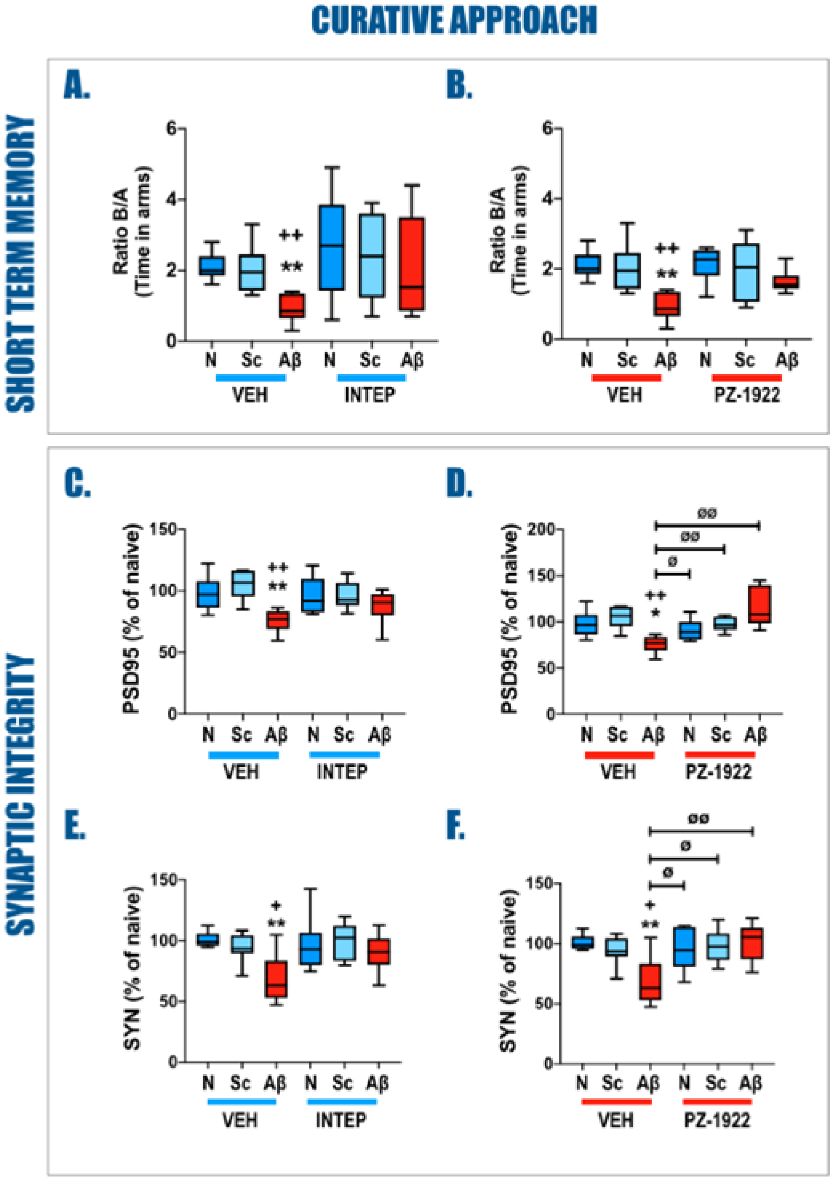

Figure S-8. Impact of curative strategy with intepirdine (INTEP) and **PZ-1922** on $A\beta_{25-35}$-induced toxicity. (**A,B**) Spatial short-term memory performance was determined in a T-maze test and was expressed as the ratio of the time spent in the initially closed arm (B) over the time spent in the previous arm (A). (**C-F**) Synaptic Integrity in the hippocampus was evaluated by Western blot. Variations of post- (PSD95, 95 kDa) (**C,D**) and pre-synaptic (SYN, 65 kDa) (**E,F**) markers in the whole hippocampus were evaluated in each group, normalized with the respective variations of β-tubulin (β-tub, 55 kDa) and expressed in percent of variations obtained in non-injected rats (Naive group). For experimental protocol see Figure 6A. All data are presented as box & whiskers with Min to Max and Median with n = 8 for Naive (N), Scrambled (Sc) and $A\beta_{25-35}$ (Aβ) rats treated with vehicle (VEH); and n = 6 for N, Sc and Aβ rats treated with intepirdine (INTEP) or **PZ-1922**. Two-way ANOVA followed by Tukey's multiple comparison was performed (See Table S-6). * $p < 0.05$ and ** $p < 0.01$ vs. respective naive (N) group; + $p < 0.05$ and ++ $p < 0.01$ vs. respective scrambled (Sc) group; ø $p < 0.05$ and øø $p < 0.01$ *vs* selected group.

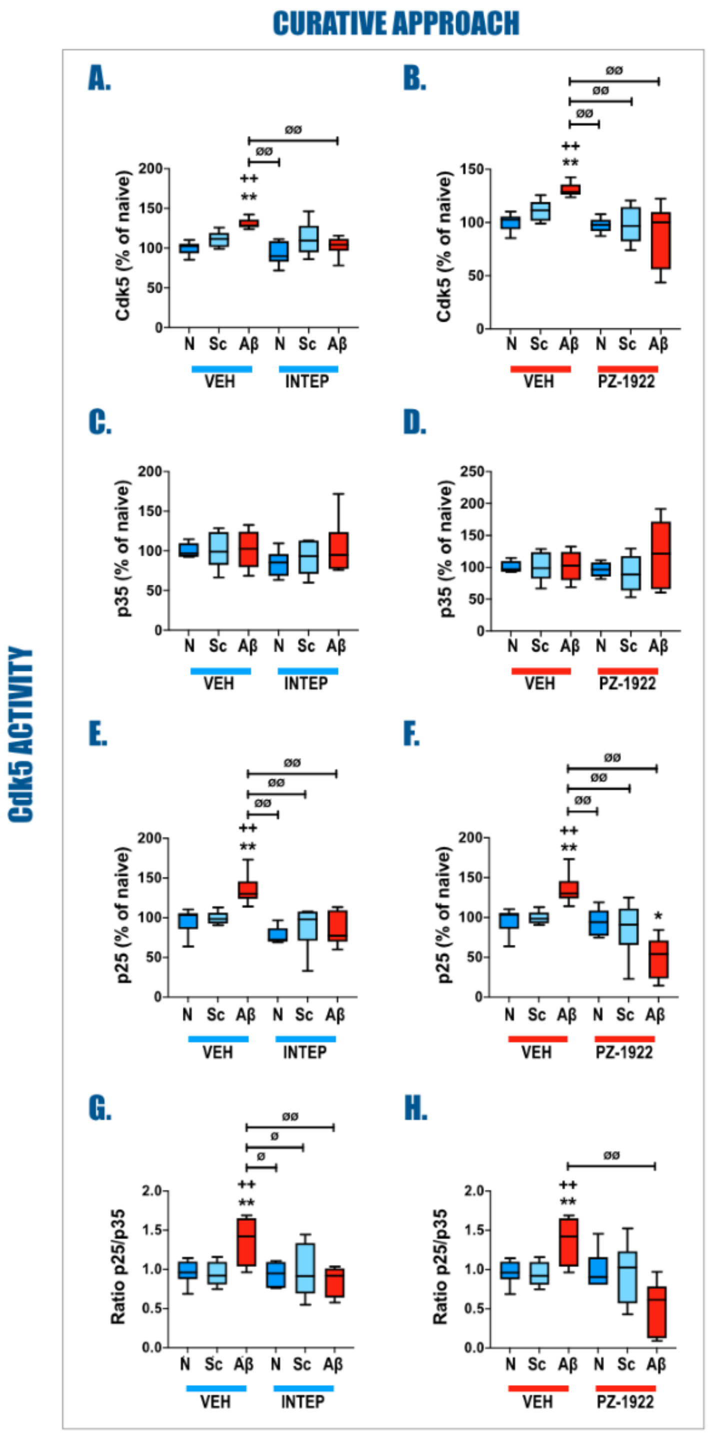

Figure S-9. The impact in the hippocampus of the curative strategy with intepirdine (INTEP) and **PZ-1922** on Cdk5 activity (A-H) induced by the *icv* injection of $A\beta_{25-35}$ were evaluated by Western blot. Levels of Cdk5 (30 kDa) (**A,B**); p35 (35 kDa) (**C,D**), p25 (25 kDa) (**E,F**) and the level ratio of p25 / p35 (**G,H**) were evaluated in each group, normalized with the variations of β-tubulin (β-tub, 55 kDa) and expressed in percent of variations obtained in non-injected rats (Naive group). For experimental protocol see Figure 6A. All data are presented as box & whiskers with Min to Max and Median with n = 8 for Naive (N), Scrambled (Sc) and $A\beta_{25-35}$ (Aβ) rats treated with vehicle (VEH); and n = 6 for N, Sc and Aβ rats treated with intepirdine (INTEP) or **PZ-1922**. Two-way ANOVA followed by Tukey's multiple comparison was performed (See Table S-6). * $p< 0.05$ and ** $p< 0.01$ vs. respective naive (N) group; + $p< 0.05$ and ++ $p< 0.01$ vs. respective scrambled (Sc) group; ø $p< 0.05$ and øø $p< 0.01$ *vs.* selected group.

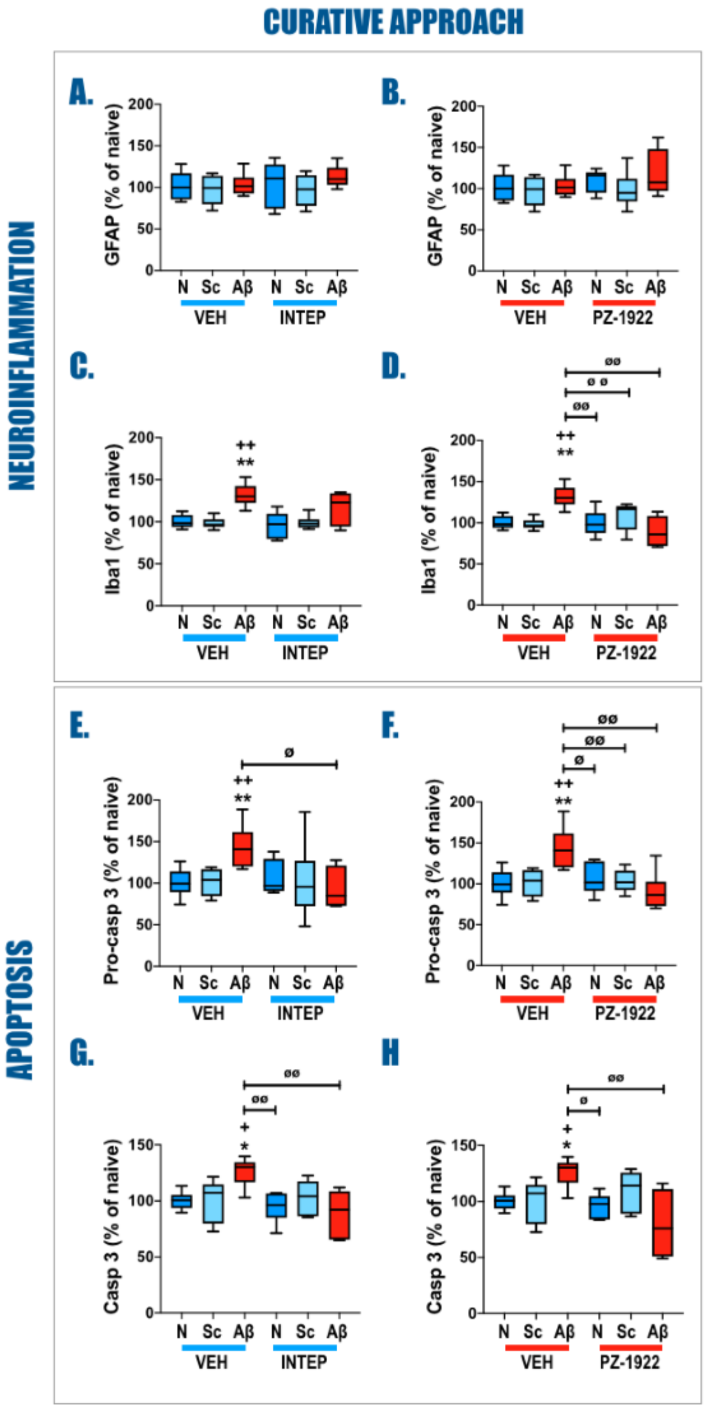

Figure S-10. The impact in the hippocampus of the curative strategy with intepirdine (INTEP) and **PZ-1922** on neuroinflammation (A-D) and apoptotic processes (E-H) induced by the *icv* injection of $A\beta_{25-35}$ were evaluated by Western blot. Levels of GFAP (50 kDa) (**A,B**); Iba1 (17 kDa) (**C,D**), Pro-casp 3 (35 kDa) (**E,F**) and Casp 3 (19 kDa) (**G,H**) were evaluated in each group, normalized with the variations of β-tubulin (β-tub, 55 kDa) and expressed in percent of variations obtained in non-injected rats (Naive group). For experimental protocol see Figure 6A. All data are presented as box & whiskers with Min to Max and Median with n = 8 for Naive (N), Scrambled (Sc) and $A\beta_{25-35}$ (Aβ) rats treated with vehicle (VEH); and n = 6 for N, Sc and Aβ rats treated with intepirdine (INTEP) or **PZ-1922**. Two-way ANOVA followed by Tukey's multiple comparison was performed (See Table S-6). * $p< 0.05$ and ** $p< 0.01$ vs. respective naive (N) group; + $p< 0.05$ and ++ $p< 0.01$ vs. respective scrambled (Sc) group; ø $p< 0.05$ and øø $p< 0.01$ *vs* selected group.

9. Results of the biochemical analysis of preventive treatment with PZ-1922 and intepirdine

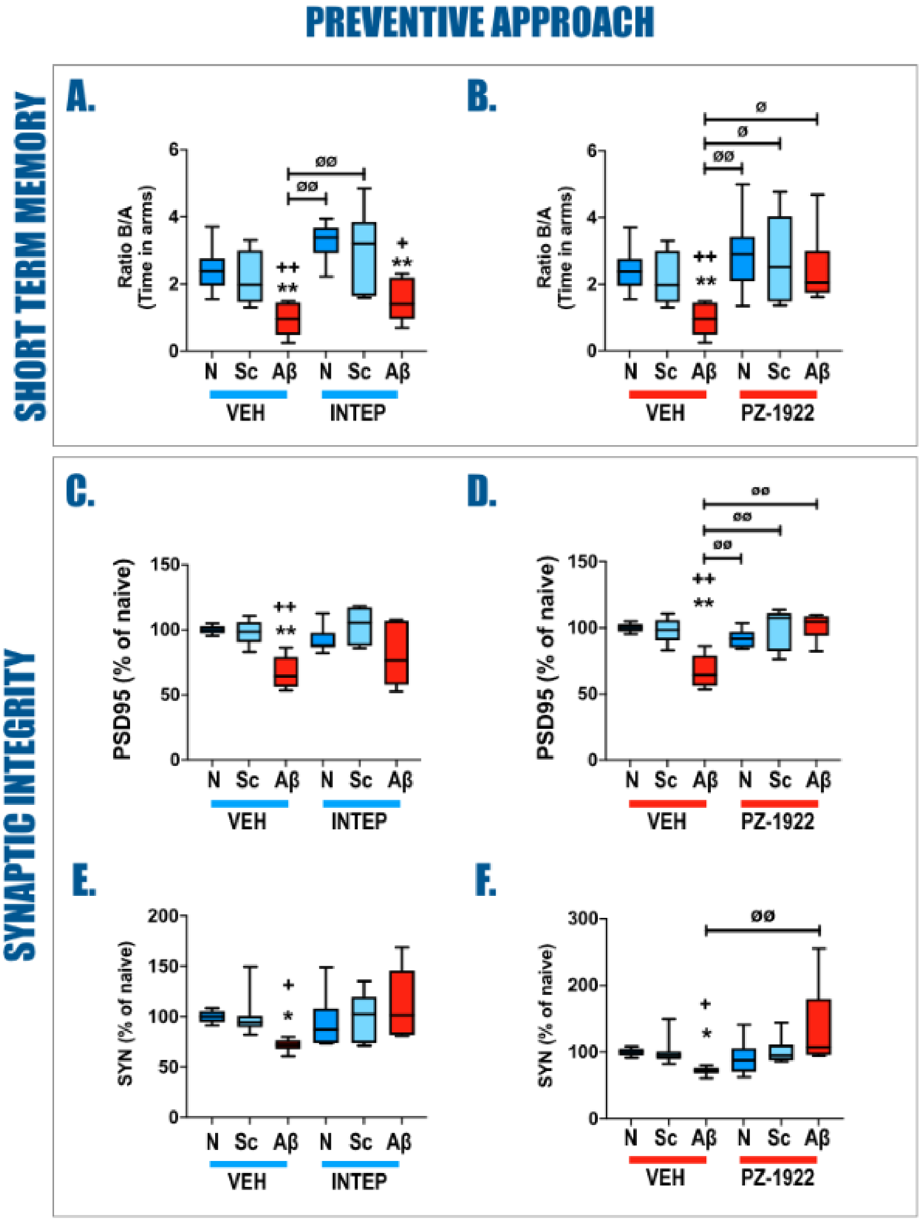

Figure S-11. Impact of preventive strategy with intepirdine (INTEP) and **PZ-1922** on $A\beta_{25\text{-}35}$-induced toxicity. (**A,B**) Spatial short-term memory performance was determined in a T-maze test and was expressed as the ratio of the time spent in the initially closed arm (B) over the time spent in the previous arm (A). (**C-F**) Synaptic Integrity in the hippocampus was evaluated by Western blot. Variations of post- (PSD95, 95 kDa) (**C,D**) and pre-synaptic (SYN, 65 kDa) (**E,F**) markers in the whole hippocampus were evaluated in each group, normalized with the respective variations of β-tubulin (β-tub, 55 kDa) and expressed in percent of variations obtained in non-injected rats (Naive group). For experimental protocol see Figure 6G. All data are presented as box & whiskers with Min to Max and Median with n = 8 for Naive (N), Scrambled (Sc) and $A\beta_{25\text{-}35}$ (Aβ) rats treated with vehicle (VEH); and n = 6 for N, Sc and Aβ rats treated with intepirdine (INTEP) or **PZ-1922**. Two-way ANOVA followed by Tukey's multiple comparison was performed (See Table S-6). * $p< 0.05$ and ** $p< 0.01$ vs. respective naive (N) group; + $p< 0.05$ and ++ $p< 0.01$ vs. respective scrambled (Sc) group; ø $p< 0.05$ and øø $p< 0.01$ *vs* selected group.

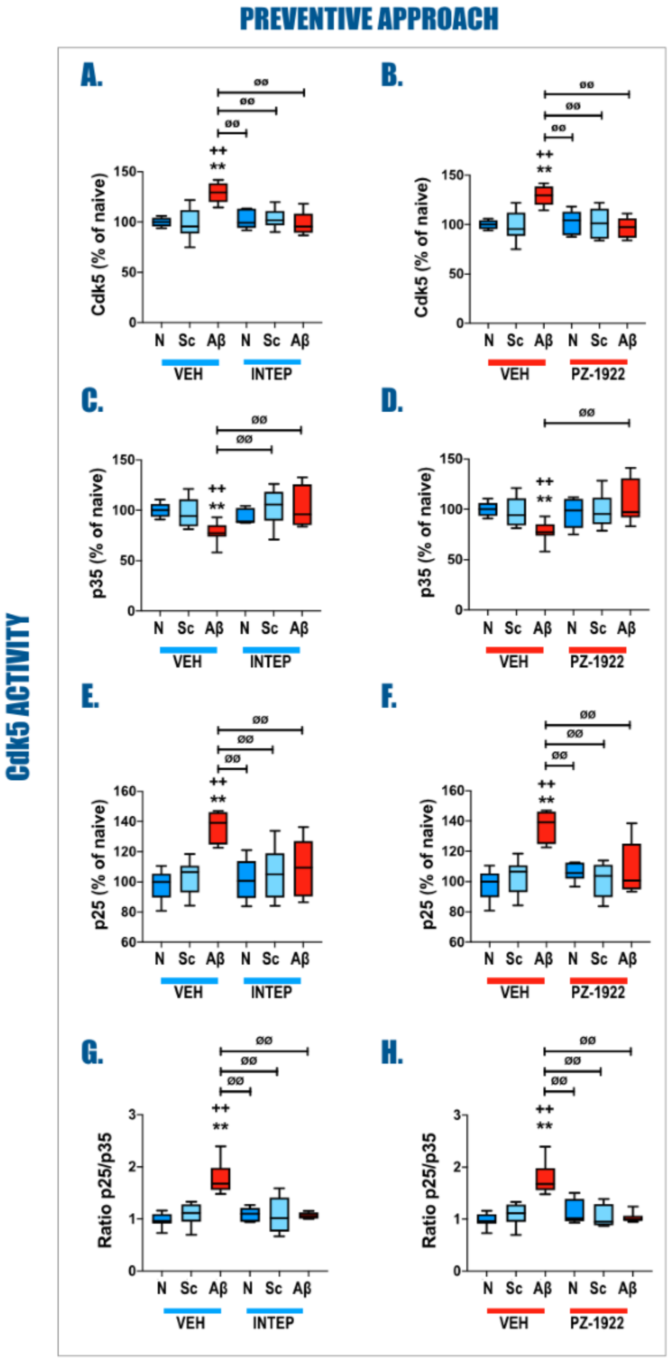

Figure S-12. The impact in the hippocampus of the preventive strategy with intepirdine (INTEP) and **PZ-1922** on Cdk5 activity (A-H) induced by the *icv* injection of $A\beta_{25\text{-}35}$ were evaluated by Western blot. Levels of Cdk5 (30 kDa) (**A,B**); p35 (35 kDa) (**C,D**), p25 (25 kDa) (**E,F**) and the level ratio of p25 / p35 (**G,H**) were evaluated in each group, normalized with the variations of β-tubulin (β-tub, 55 kDa) and expressed in percent of variations obtained in non-injected rats (Naive group). For experimental protocol see Figure 6G. All data are presented as box & whiskers with Min to Max and Median with n = 8 for Naive (N), Scrambled (Sc) and $A\beta_{25\text{-}35}$ (Aβ) rats treated with vehicle (VEH); and n = 6 for N, Sc and Aβ rats treated with intepirdine (INTEP) or **PZ-1922**. Two-way ANOVA followed by Tukey's multiple comparison was performed (See Table S-6). * $p< 0.05$ and ** $p< 0.01$ vs. respective naive (N) group; + $p< 0.05$ and ++ $p< 0.01$ *vs* respective scrambled (Sc) group; ø $p< 0.05$ and øø $p< 0.01$ *vs* selected group.

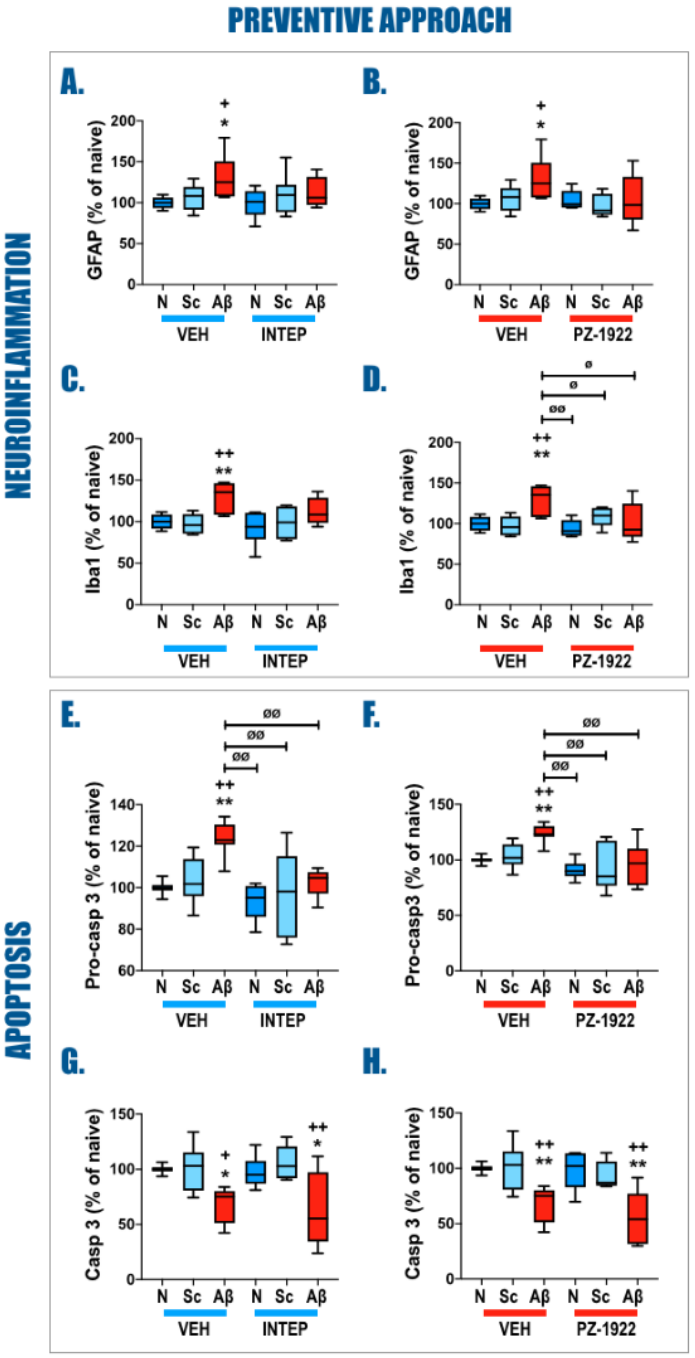

Figure S-13. The impact in the hippocampus of the preventive strategy with intepirdine (INTEP) and **PZ-1922** on neuroinflammation (A-D) and apoptotic processes (E-H) induced by the *icv* injection of $A\beta_{25-35}$ were evaluated by Western blot. Levels of GFAP (50 kDa) (**A,B**); Iba1 (17 kDa) (**C,D**), Pro-casp 3 (35 kDa) (**E,F**) and Casp 3 (19 kDa) (**G,H**) were evaluated in each group, normalized with the variations of β-tubulin (β-tub, 55 kDa) and expressed in percent of variations obtained in non-injected rats (Naive group). For experimental protocol see Figure 6G. All data are presented as box & whiskers with Min to Max and Median with n = 8 for Naive (N), Scrambled (Sc) and $A\beta_{25-35}$ (Aβ) rats treated with vehicle (VEH); and n = 6 for N, Sc and Aβ rats treated with intepirdine (INTEP) or **PZ-1922**. Two-way ANOVA followed by Tukey's multiple comparison was performed (See Table S-6). * $p< 0.05$ and ** $p< 0.01$ *vs* respective naive (N) group; + $p< 0.05$ and ++ $p< 0.01$ vs. respective scrambled (Sc) group; ø $p< 0.05$ and øø $p< 0.01$ *vs* selected group.

Table S-3. Antibodies used in Western blot experiments

Protein	Mol. weight	Host	Dilution	Ref.	Supplier
**Primary antibodies**					
Caspase 3	35/19 kDa	Rabbit	1/500	#9665	Cell Signaling/Ozyme, France
Cdk5	30 kDa	Rabbit	1/500	#2506	Cell Signaling/Ozyme, France
GFAP	55 kDa	Mouse	1/2000	G3893	Sigma-Aldrich, France
Iba1	17 kDa	Rabbit	1/750	013-19741	Wako Chem, Japan
p35/p25	35/25 kDa	Rabbit	1/500	#2680	Cell Signaling/Ozyme, France
PSD95	95 kDa	Rabbit	1/2000	#3450	Cell Signaling/Ozyme, France
SYN	65 kDa	Mouse	1/1000	MAB5200	Merck-Millipore, France
β-Tubulin	50 kDa	Mouse	1/7500	T4026	Sigma-Aldrich, France
**Secondary antibodies**					
IgG	Goat anti-rabbit IgG peroxidase conjugate		1/2000	A61-54	Sigma-Aldrich, France
IgG	Goat anti-mouse IgG peroxidase conjugate		1/2000	A67-82	Sigma-Aldrich, France

Table S-4. Statistical analysis of principal figures

	Figure	Experiment	ANOVA one-way / Tukey's or Dunnett's multiple comparisons test	P value
**CURATIVE APPROACH**	7B	T-maze (INTEP)	$F_{2,19} = 4.861$	P = 0.0197
	7C	T-maze (PZ-1922)	$F_{2,19} = 19.83$	P < 0.0001
	7E	WB - PSD95	$F_{3,24} = 8.658$	P = 0.0005
	7F	WB - SYN	$F_{3,24} = 7.436$	P = 0.0011
	8B	WB - Cdk5	$F_{3,24} = 9.161$	P = 0.0003
	8C	WB - p25/p35	$F_{3,24} = 13.05$	P < 0.0001
	8D	WB - GFAP	$F_{3,24} = 1.262$	*ns*
	8E	WB - Iba1	$F_{3,24} = 11.62$	P < 0.0001
**PREVENTIVE APPROACH**	7H	T-maze (INTEP)	$F_{2,19} = 12.52$	P = 0.0003
	7I	T-maze (PZ-1922)	$F_{2,19} = 9.964$	P = 0.0014
	7K	WB - PSD95	$F_{3,24} = 10.70$	P = 0.0001
	7L	WB - SYN	$F_{3,24} = 4.506$	P = 0.0121
	8G	WB - Cdk5	$F_{3,24} = 28.06$	P < 0.0001
	8H	WB - p25/p35	$F_{3,24} = 39.49$	P < 0.0001
	8I	WB - GFAP	$F_{3,24} = 3.029$	P = 0.049
	8J	WB - Iba1	$F_{3,24} = 4.695$	P = 0.0102

WB: western blot experiments; ns: no-significant; INTEP: intepirdine

Table S-5. Statistical analysis of Figure S-7.

	Figure	Experiment	ANOVA one-way / Dunnett's multiple comparisons test (*vs. Naive-V group*)	P value
**CURATIVE APPROACH**	S-7B	WB Pro-casp3	$F_{3,24} = 9.307$	P = 0.0003
	S-7C	WB Casp 3	$F_{3,24} = 8.521$	P = 0.0005
**PREVENTIVE APPROACH**	S-7E	WB Pro-casp3	$F_{3,24} = 10.23$	P = 0.0002
	S-7F	WB Casp 3	$F_{3,24} = 6.566$	P = 0.0021

WB: western blot experiments; ns: no-significant

Table S-6. Statistical analysis of supplementary figures.

	Figure	Experiment	Treatment	ANOVA Two-way Tukey's multiple comparisons test		P value
CURATIVE APPROACH	S-8A	T-maze	INTEP	*Interaction*	$F_{2,36} = 0.520$	ns
				Group	$F_{2,36} = 3.174$	P=0.0437
				Treatment	$F_{1,36} = 1.033$	P=0.0340
	S-8B		PZ-1922	*Interaction*	$F_{2,36} = 1.975$	ns
				Group	$F_{2,36} = 9.254$	P=0.0006
				Treatment	$F_{1,36} = 1.802$	ns
	S-8C	WB - PSD95	INTEP	*Interaction*	$F_{2,36} = 2.433$	ns
				Group	$F_{2,36} = 8.623$	P=0.0009
				Treatment	$F_{1,36} = 0.091$	P=0.9242
	S-8D		PZ-1922	*Interaction*	$F_{2,36} = 14.18$	P<0.0001
				Group	$F_{2,36} = 1.052$	ns
				Treatment	$F_{1,36} = 4.142$	P=0.0492
	S-8E	WB - SYN	INTEP	*Interaction*	$F_{2,36} = 2.143$	ns
				Group	$F_{2,36} = 5.832$	P=0.0064
				Treatment	$F_{1,36} = 2.134$	ns
	S-8F		PZ-1922	*Interaction*	$F_{2,36} = 6.138$	P=0.0051
				Group	$F_{2,36} = 2.873$	ns
				Treatment	$F_{1,36} = 4.716$	P=0.0365
	S-9A	WB - Cdk5	INTEP	*Interaction*	$F_{2,36} = 4.896$	P=0.0132
				Group	$F_{2,36} = 9.781$	P=0.0004
				Treatment	$F_{1,36} = 9.024$	P=0.0048
	S-9B		PZ-1922	*Interaction*	$F_{2,36} = 6.391$	P=0.0042
				Group	$F_{2,36} = 1.818$	ns
				Treatment	$F_{1,36} = 17.73$	P=0.0002
	S-9C	WB - p35	INTEP	*Interaction*	$F_{2,36} = 0.486$	ns
				Group	$F_{2,36} = 0.835$	ns
				Treatment	$F_{1,36} = 1.406$	ns
	S-9D		PZ-1922	*Interaction*	$F_{2,36} = 1.074$	ns
				Group	$F_{2,36} = 1.473$	ns
				Treatment	$F_{1,36} = 0.019$	ns
	S-9E	WB - p25	INTEP	*Interaction*	$F_{2,36} = 4.762$	P=0.0146
				Group	$F_{2,36} = 6.347$	P=0.0044
				Treatment	$F_{1,36} = 23.66$	P<0.0001
	S-9F		PZ-1922	*Interaction*	$F_{2,36} = 16.03$	P<0.0001
				Group	$F_{2,36} = 0.052$	ns
				Treatment	$F_{1,36} = 26.44$	P<0.0001
	S-9G	WB - p25/p35	INTEP	*Interaction*	$F_{2,36} = 6.316$	P=0.0045
				Group	$F_{2,36} = 2.278$	ns
				Treatment	$F_{1,36} = 5.825$	P=0.0210
	S-9H		PZ-1922	*Interaction*	$F_{2,36} = 11.87$	P=0.0001
				Group	$F_{2,36} = 0.036$	ns
				Treatment	$F_{1,36} = 9.937$	P=0.0033
	S-10A	WB - GFAP	INTEP	*Interaction*	$F_{2,36} = 0.278$	ns
				Group	$F_{2,36} = 1.421$	ns
				Treatment	$F_{1,36} = 0.462$	ns

	S-10B		PZ-1922	*Interaction*	$F_{2,36}$ = 0.450	ns
				Group	$F_{2,36}$ = 1.770	ns
				Treatment	$F_{1,36}$ = 1.992	ns
	S-10C	WB - Iba1	INTEP	*Interaction*	$F_{2,36}$ = 1.454	ns
				Group	$F_{2,36}$ = 20.46	P<0.0001
				Treatment	$F_{1,36}$ = 2.564	ns
	S-10D		PZ-1922	*Interaction*	$F_{2,36}$ = 15.39	P<0.0001
				Group	$F_{2,36}$ = 2.196	ns
				Treatment	$F_{1,36}$ = 7.168	P=0.0111
	S-10E	WB – Pro-casp3	INTEP	*Interaction*	$F_{2,36}$ = 4.953	P=0.0126
				Group	$F_{2,36}$ = 1.794	ns
				Treatment	$F_{1,36}$ = 3.342	ns
	S-10F		PZ-1922	*Interaction*	$F_{2,36}$ = 9.808	P=0.0004
				Group	$F_{2,36}$ = 2.461	ns
				Treatment	$F_{1,36}$ = 6.476	P=0.0154
	S-10G	WB – Casp3	INTEP	*Interaction*	$F_{2,36}$ = 6.521	P=0.0038
				Group	$F_{2,36}$ = 1.565	ns
				Treatment	$F_{1,36}$ = 8.511	P=0.0060
	S-10H		PZ-1922	*Interaction*	$F_{2,36}$ = 9.589	P=0.0005
				Group	$F_{2,36}$ = 0.6165	ns
				Treatment	$F_{1,36}$ = 6.766	P=0.0134
**PREVENTIVE APPROACH**	S-11A	T-maze	INTEP	*Interaction*	$F_{2,36}$ = 0.178	ns
				Group	$F_{2,36}$ = 18.34	P<0.0001
				Treatment	$F_{1,36}$ = 10.16	P=0.0030
	S-11B		PZ-1922	*Interaction*	$F_{2,36}$ = 1.217	ns
				Group	$F_{2,36}$ = 4.039	P=0.0261
				Treatment	$F_{1,36}$ = 1.802	P=0.0062
	S-11C	WB - PSD95	INTEP	*Interaction*	$F_{2,36}$ = 2.143	ns
				Group	$F_{2,36}$ = 16.70	P<0.0001
				Treatment	$F_{1,36}$ = 0.563	ns
	S-11D		PZ-1922	*Interaction*	$F_{2,36}$ = 15.93	P<0.0001
				Group	$F_{2,36}$ = 7.969	P=0.0014
				Treatment	$F_{1,36}$ = 8.353	P=0.0065
	S-11E	WB - SYN	INTEP	*Interaction*	$F_{2,36}$ = 4.701	P=0.0153
				Group	$F_{2,36}$ = 0.501	ns
				Treatment	$F_{1,36}$ = 2.923	ns
	S-11F		PZ-1922	*Interaction*	$F_{2,36}$ = 6.629	P=0.0035
				Group	$F_{2,36}$ = 0.2879	ns
				Treatment	$F_{1,36}$ = 4.487	P=0.0411
	S-12A	WB - Cdk5	INTEP	*Interaction*	$F_{2,36}$ = 13.25	P<0.0001
				Group	$F_{2,36}$ = 7.601	P=0.0018
				Treatment	$F_{1,36}$ = 9.568	P=0.0038
	S-12B		PZ-1922	*Interaction*	$F_{2,36}$ = 11.11	P=0.0002
				Group	$F_{2,36}$ = 5.681	P=0.0072
				Treatment	$F_{1,36}$ = 9.224	P=0.0044
	S-12C	WB - p35	INTEP	*Interaction*	$F_{2,36}$ = 5.978	P=0.0057
				Group	$F_{2,36}$ = 1.887	ns
				Treatment	$F_{1,36}$ = 4.443	P=0.0421
	S-12D		PZ-1922	*Interaction*	$F_{2,36}$ = 5.879	P=0.0062
				Group	$F_{2,36}$ = 1.019	ns
				Treatment	$F_{1,36}$ = 4.707	P=0.0367

	S-12E	WB - p25	INTEP	*Interaction*	$F_{2,36}$ = 6.236	P=0.0047
				Group	$F_{2,36}$ = 13.03	P<0.0001
				Treatment	$F_{1,36}$ = 5.307	P=0.0271
	S-12F		PZ-1922	*Interaction*	$F_{2,36}$ = 11.97	P=0.0001
				Group	$F_{2,36}$ = 18.10	P<0.0001
				Treatment	$F_{1,36}$ = 8.998	P=0.0049
	S-12G	WB - p35/p25	INTEP	*Interaction*	$F_{2,36}$ = 13.41	P<0.0001
				Group	$F_{2,36}$ = 12.19	P<0.0001
				Treatment	$F_{1,36}$ = 7.650	P=0.0089
	S-12H		PZ-1922	*Interaction*	$F_{2,36}$ = 17.74	P<0.0001
				Group	$F_{2,36}$ = 11.50	P=0.0001
				Treatment	$F_{1,36}$ = 9.467	P=0.0040
	S-13A	WB - GFAP	INTEP	*Interaction*	$F_{2,36}$ = 1.279	ns
				Group	$F_{2,36}$ = 4.599	P=0.0167
				Treatment	$F_{1,36}$ = 0.8220	ns
	S-13B		PZ-1922	*Interaction*	$F_{2,36}$ = 2.217	ns
				Group	$F_{2,36}$ = 3.266	P=0.0441
				Treatment	$F_{1,36}$ = 3.037	ns
	S-13C	WB - Iba1	INTEP	*Interaction*	$F_{2,36}$ = 1.213	ns
				Group	$F_{2,36}$ = 9.952	P=0.0004
				Treatment	$F_{1,36}$ = 2.188	ns
	S-13D		PZ-1922	*Interaction*	$F_{2,36}$ = 6.029	P=0.0055
				Group	$F_{2,36}$ = 5.428	P=0.0087
				Treatment	$F_{1,36}$ = 2.407	ns
	S-13E	WB – Pro-casp3	INTEP	*Interaction*	$F_{2,36}$ = 2.026	ns
				Group	$F_{2,36}$ = 8.480	P=0.0010
				Treatment	$F_{1,36}$ = 10.71	P=0.0024
	S-13F		PZ-1922	*Interaction*	$F_{2,36}$ = 2,120	ns
				Group	$F_{2,36}$ = 5.083	P=0.0114
				Treatment	$F_{1,36}$ = 15.74	P=0.0003
	S-13G	WB – Casp3	INTEP	*Interaction*	$F_{2,36}$ = 0.2356	ns
				Group	$F_{2,36}$ = 17.33	P<0.0001
				Treatment	$F_{1,36}$ = 0.034	ns
	S-13H		PZ-1922	*Interaction*	$F_{2,36}$ = 0.3311	ns
				Group	$F_{2,36}$ = 22.50	P<0.0001
				Treatment	$F_{1,36}$ = 2.073	ns

WB: western blot experiments; INTEP: intepiridine; ns: no-significant

References

(1) Grychowska, K.; Olejarz-Maciej, A.; Blicharz, K.; Pietruś, W.; Karcz, T.; Kurczab, R.; Koczurkiewicz, P.; Doroz-Płonka, A.; Latacz, G.; Keeri, A. R.; Piska, K.; Satała, G.; Pęgiel, J.; Trybała, W.; Jastrzębska-Więsek, M.; Bojarski, A. J.; Lamaty, F.; Partyka, A.; Walczak, M.; Krawczyk, M.; Malikowska-Racia, N.; Popik, P.; Zajdel, P. Overcoming undesirable *h*ERG affinity by incorporating fluorine atoms: A case of MAO-B inhibitors derived from 1*H*-pyrrolo-[3,2-*c*]quinolines. *Eur. J. Med. Chem.* **2022**, *236*, 114329.

(2) Canale, V.; Grychowska, K.; Kurczab, R.; Ryng, M.; Keeri, A. R.; Satała, G.; Olejarz-Maciej, A.; Koczurkiewicz, P.; Drop, M.; Blicharz, K.; Piska, K.; Pękala, E.; Janiszewska, P.; Krawczyk, M.; Walczak, M.; Chaumont-Dubel, S.; Bojarski, A. J.; Marin, P.; Popik, P.; Zajdel, P. A dual-acting 5-HT_6 receptor inverse agonist/MAO-B inhibitor displays glioprotective and pro-cognitive properties. *Eur. J. Med. Chem.* **2020**, *208*, 112765.
